# Systematic Association Mapping Identifies *NELL1* as a Novel IBD Disease Gene

**DOI:** 10.1371/journal.pone.0000691

**Published:** 2007-08-08

**Authors:** Andre Franke, Jochen Hampe, Philip Rosenstiel, Christian Becker, Florian Wagner, Robert Häsler, Randall D. Little, Klaus Huse, Andreas Ruether, Tobias Balschun, Michael Wittig, Abdou ElSharawy, Gabriele Mayr, Mario Albrecht, Natalie J. Prescott, Clive M. Onnie, Hélène Fournier, Tim Keith, Uwe Radelof, Matthias Platzer, Christopher G. Mathew, Monika Stoll, Michael Krawczak, Peter Nürnberg, Stefan Schreiber

**Affiliations:** 1 Institute for Clinical Molecular Biology, Christian-Albrechts University Kiel, Kiel, Germany; 2 First Department of Medicine, University Hospital Schleswig-Holstein, Kiel, Germany; 3 Cologne Center for Genomics, University of Cologne, Köln, Germany; 4 RZPD German Resource Center for Genome Research, Berlin, Germany; 5 Genizon BioSciences5, Québec, Canada; 6 Genome Analysis Group, Leibniz Institute for Age Research, Jena, Germany; 7 PopGen Biobank, Christian-Albrechts University Kiel, Kiel, Germany; 8 Max-Planck Institute for Informatics, Saarbrücken, Germany; 9 Department of Medical and Molecular Genetics, King's College London School of Medicine, London, United Kingdom; 10 Leibniz-Institute for Arteriosclerosis Research, University Münster, Münster, Germany; 11 Institute of Medical Statistics and Informatics, Christian-Albrechts University Kiel, Kiel, Germany; 12 Center for Molecular Medicine Cologne, University of Cologne, Köln, Germany; North Carolina State University, United States of America

## Abstract

Crohn disease (CD), a sub-entity of inflammatory bowel disease (IBD), is a complex polygenic disorder. Although recent studies have successfully identified CD-associated genetic variants, these susceptibility loci explain only a fraction of the heritability of the disease. Here, we report on a multi-stage genome-wide scan of 393 German CD cases and 399 controls. Among the 116,161 single-nucleotide polymorphisms tested, an association with the known CD susceptibility gene *NOD2*, the *5q31* haplotype, and the recently reported CD locus at *5p13.1* was confirmed. In addition, SNP rs1793004 in the gene encoding nel-like 1 precursor (*NELL1,* chromosome *11p15.1*) showed a consistent disease-association in independent German population- and family-based samples (942 cases, 1082 controls, 375 trios). Subsequent fine mapping and replication in an independent sample of 454 French/Canadian CD trios supported the authenticity of the *NELL1* association. Further confirmation in a large German ulcerative colitis (UC) sample indicated that *NELL1* is a ubiquitous IBD susceptibility locus (combined p<10^−6^; OR = 1.66, 95% CI: 1.30–2.11). The novel *5p13.1* locus was also replicated in the French/Canadian sample and in an independent UK CD patient panel (453 cases, 521 controls, combined p<10^−6^ for SNP rs1992660). Several associations were replicated in at least one independent sample, point to an involvement of *ITGB6* (upstream), *GRM8* (downstream), *OR5V1* (downstream), *PPP3R2* (downstream), *NM_152575* (upstream) and *HNF4G* (intron).

## Introduction

An estimated 1.4 million individuals in the United States and 2.2 million individuals in Europe suffer from inflammatory bowel disease [1] (IBD, MIM 601458, 266600, 191390), a life-long disease that occurs in the form of one of two major sub-phenotypes, Crohn disease (CD) or ulcerative colitis (UC). The pathophysiology of IBD is characterized by a highly activated state of the mucosal immune system and excessive mucosal destruction. The enteric flora appears to play a key role as a stimulating agent [2]. Familial clustering [3], [4] and an increased concordance rate of IBD among monozygotic twins [5], [6] are hallmarks of the genetic aetiology of IBD, a notion that is further supported by the discovery of several disease genes. This includes *NOD2* [7]–[9] (*IBD1*), a risk haplotype in the *5q31* (*IBD5*) locus [10], [11], *DLG5* [12], [13], *TNFSF15* [14], *ATG16L1* [15], *CARD4* [16], and recently *IL23R* [17].

High-density SNP arrays have enabled genome-wide association scans (GWS) to be performed at reasonable costs. Yamazaki and colleagues reported the first GWS for CD, which resulted in the identification of associated polymorphisms in the *TNFSF15* gene [14]. Recently, two other GWS reported the novel CD susceptibility loci *IL23R* [17] and *5p13.1* [18]. We recently performed a genome-wide candidate gene analysis, using 19,779 non-synonymous SNPs, which led to the identification of a common variant (T300A) in the *ATG16L1* gene as predisposing to CD [15], a finding that was later replicated by four other groups [18]–[21].

Here, we report upon the identification of additional risk loci for CD through a multi-stage genome-wide association scan (Figure S1) in 393 German cases and 399 German population-representative controls, using the Affymetrix GeneChip® Human Mapping 100K Set [22]. In order to enrich our samples with risk alleles [23] and to reduce phenotypic heterogeneity, CD patients in the GWS were selected for a “severe” phenotype, including a positive IBD family history, age of onset≤25 years, and no change in diagnosis over the last five years. The SNPs representing the top 200 association leads were re-genotyped in both, a large independent German case-control sample and a family-based sample comprising 375 nuclear families. In addition to replicating *NOD2*, *IBD5*, and *5p13.1*, a novel susceptibility locus was identified on chromosome *11p15.1*, namely the nel-like 1 precursor-encoding gene (*NELL1*).

## Results

### Genome-wide association scan

A total of 116,161 SNPs were genotyped in case-control panel A (Table 1). Of these, 92,387 SNPs had a call rate≥90%, were polymorphic in panel A, and showed no significant departure from Hardy-Weinberg equilibrium (p_HWE_≤0.01 in controls) (Table S1A). At an unadjusted per-test significance level of 5%, the experiment had 80% power to detect an odds ratio of 1.6, and 33% power to detect an odds ratio of 1.3, assuming that 20% of the controls were carriers of the risk factor (Figure S2). The GWS results were not corrected for potential population substructure because (i) very low (<10^−3^) F_ST_ values have previously been reported for different geographic regions of Germany [24], (ii) patients of panel A were all selected from the Northern part of Germany, and were therefore geographically matched to the population-representative controls from the POPGEN biobank [25], (iii) quantile-quantile plots, which can help to identify spurious association results [26], revealed no inflation of the χ^2^ statistics (Figure S3), and (iv) replication criteria included confirmation by family-based association tests (transmission disequilibrium test, TDT), which are robust against population stratification [27].

**Table 1:** Crohn disease (CD) patient, ulcerative colitis (UC) patient, and replication samples used for association analyses.

Panel	Patients	Controls	Trios
CD Germany–A	393	399	-
CD Germany-B	942	1082	375
UC Germany–C	1059		419
CD QFP (French/Canadian)-D	466	358	454
CD UK–E	453	521	-

The patient samples were organized in panels that correspond to successive steps of the study. Index cases from trios were also used in the case-control analyses so that, for example, a total of 942 cases (567+375) were available for the case-control comparison in panel B.

### Replication

The 200 most significant SNPs in the GWS were next genotyped in two independent German samples (panel B, Table S2). “Replication” was considered to have been achieved if the p-values of both, the case-control analysis and the family-based TDT were <5% (Table 2). Replication in two independent samples also rendered test-wise Bonferroni correction superfluous, which would have been overly conservative in a replication setting anyway [28].

**Table 2:** Markers showing a significant CD association in the case-control and the family-based analyses in panel B.

SNP Information					Screening (Panel A)					Replication (Panel B)					
Rank	dbSNP ID	Chr.	Position	Locus	MAF_co_	MAF_ca_	p_CCA_	p_CCG_	OR (95% CI)	MAF_co_	MAF_ca_	p_CCA_	p_CCG_	OR (95% CI)	p_TDT_
**1**	**rs2076756**	**16**	**49,314,382**	***CARD15, intron***	**0.26**	**0.43**	**1.93E-13**	**2.04E-12**	**2.1 (1.58-2.80)**	**0.27**	**0.41**	**6.80E-20**	**1.39E-21**	**1.71 (1.42-2.05)**	**5.90E-08**
70	rs1992662	5	40,429,609	*PTGER4, upstream*	0.33	0.25	0.00047	0.0018	0.60 (0.45–0.80)	0.32	0.26	7.59E-05	0.00017	0.76 (0.63–0.91)	0.0013
75	rs1992660	5	40,450,824	*PTGER4, upstream*	0.40	0.31	0.00050	0.0020	0.64 (0.48–0.86)	0.38	0.31	4.53E-05	0.00021	0.72 (0.60–0.86)	0.00050
83	rs1793004	11	20,655,505	*NELL1, intron*	0.27	0.20	0.00053	0.0020	0.59 (0.44–0.80)	0.28	0.24	0.025	0.052	0.85 (0.71–1.00)	0.045
**159**	**rs10521209**	**16**	**49,313,210**	***CARD15, intron***	**0.41**	**0.33**	**0.0013**	**0.0031**	**0.70 (0.52–0.93)**	**0.41**	**0.35**	**8.65E-05**	**8.68E-05**	**0.67 (0.56–0.80)**	**0.0083**
**163**	**rs2631372**	**5**	**131,731,477**	***SLC22A4, downstream***	**0.31**	**0.28**	**0.26**	**0.0013**	**1.06 (0.80–1.41)**	**0.33**	**0.29**	**0.0080**	**0.022**	**0.78 (0.65–0.93)**	**0.030**

P-values obtained in an allele- (p_CCA_) or genotype-based (p_CCG_) case-control comparison in panel A are shown. Also included are p_CCA_, p_CCG_, and the TDT results (p_TDT_) for replication panel B. Nucleotide positions refer to NCBI build 35. Known susceptibility loci for CD are highlighted by bold type. A complete list of all 200 genotyped SNPs is given in Table S2. MAF_co_ and MAF_ca_ denote the minor allele frequency in controls and cases, respectively. Rank: rank of SNP according to the p-value obtained in screening panel A. Odds Ratios (OR) and corresponding 95% confidence intervals (95% CI) are given for carriership of the rare allele of each SNP.

In addition to rs2631372 (#163, Table S2), which localizes to the *5q31* haplotype [10], an association with CD was confirmed for rs2076756 (#1, p_CCA_<10^−12^) and rs10521209 (#159) in *NOD2* [7]–[9]. The recently reported *5p13.1* locus [18] was also replicated (rs1992660/#70, rs1992662/#75), and a novel susceptibility gene, *NELL1*, was identified (rs1793004/#83). While only these six SNPs were found to be nominally significant in both, the TDT and the case-control analysis, and therefore fulfilled the formal replication criteria, 47 SNPs were significant in only one test, including two more SNPs in the *5p13.1* region (#79, #105), one in *NELL1* (#116), and one in the *IBD5* region (#191). In view of the low power of the TDT, it appears worth mentioning that use of p_CCA_ or p_CCG_ ≤10^−2^ as the sole replication criterion would have led to the additional acceptance of rs2925757 (*ITGB6*, upstream), rs6947579 (*GRM8*, downstream), rs10484545 (*OR5V1*, downstream), rs4743484 (*PPP3R2*, downstream), rs7868736 (NM_152575, upstream), and rs830772 (*HNF4G*, intron) as confirmed associations (see Table 3).

**Table 3:** Makers that were significant only in the case-control analysis (p≤10^−2^), but not in the TDT. For column header descriptions see Table 2.

SNP Information					Screening (Panel A)					Replication (Panel B)					
Rank	dbSNP ID	Chr.	Position	Locus	MAF_co_	MAF_ca_	p_CCA_	p_CCG_	OR (95% CI)	MAF_co_	MAF_ca_	p_CCA_	p_CCG_	OR (95% CI)	p_TDT_
35	rs2925757	2	161,303,713	*ITGB6, upstream*	0.15	0.22	0.00021	0.0014	1.68 (1.24–2.26)	0.16	0.19	0.0035	0.016	1.30 (1.08–1.58)	1.00
72	rs6947579	7	125,087,495	*GRM8, downstream*	0.26	0.34	0.00048	0.0022	1.51 (1.14–2.00)	0.29	0.33	0.0065	0.013	1.18 (0.99–1.41)	1.00
79	rs1553575	5	40,548,433	*PTGER4, upstream*	0.38	0.29	0.00051	0.0021	0.65 (0.49–0.87)	0.35	0.28	1.68E-06	6.37E-06	0.65 (0.55–0.78)	0.051
86	rs10484545	6	29,342,503	*OR5V1, downstream*	0.11	0.06	0.00054	0.0017	0.50 (0.34–0.73)	0.07	0.10	0.00030	2.84E-07	1.28 (1.00–1.63)	0.16
125	rs4743484	9	99,859,318	*PPP3R2, intron*	0.26	0.19	0.00083	0.0025	0.61 (0.46–0.81)	0.25	0.21	0.0016	0.0069	0.78 (0.65–0.93)	0.90
171	rs7868736	9	111,904,228	*NM_152575, upstream*	0.24	0.31	0.0014	0.0016	1.39 (1.04–1.84)	0.25	0.29	0.0025	0.0065	1.24 (1.04–1.48)	0.64
**191**	**rs272867**	**5**	**131,757,273**	***SLC22A4, downstream***	**0.40**	**0.37**	**0.15**	**0.0018**	**1.05 (0.78–1.40)**	**0.43**	**0.38**	**0.0034**	**0.0085**	**0.74 (0.62–0.90)**	**0.26**
192	rs830772	8	76,402,540	*HNF4G, intron*	0.15	0.19	0.012	0.0019	1.26 (0.93–1.71)	0.15	0.17	0.23	0.0099	1.21 (1.00–1.47)	0.14

We did not detect our previously reported CD associations of *ATG16L1* [15], and *DLG5* [12] in this screening and did not see any association with and *IL23R* [16]. As detailed in the legend to Table 4, SNP coverage around these genes was very low. In order to benchmark our experiments, relevant SNPs in these genes were therefore genotyped in panels A and B, using TaqMan technology, and a disease association was observed for SNPs in all three genes. Interestingly, haplotype A-tagging SNP e26 in the *DLG5* gene was replicated (over-transmission of common allele T, T∶U = 275∶219, p = 0.012), while the associations of non-synonymous SNPs Arg30Gln and Pro1371Gln did not reach statistical significance.

**Table 4:** Summary of association statistics for *NELL1* and *5p13.1* SNPs as obtained in individual panels. In addition, results are shown for the two common *NELL1* nsSNPs rs8176785 and rs8176786.

SNP Information				Screening panel A (German CD)							Replication panel B (German CD)										Panel C (German UC)									
Locus	dbSNP ID	A1	A2	CR	p_HWE_	MAF_co_	MAF_ca_	p_CCA_	p_CCG_	OR (95% CI)	CR	p_HWE_	MAF_co_	MAF_ca_	p_CCA_	p_CCG_	OR (95% CI)	T∶U	OA	p_TDT_	CR	p_HWE_	MAF_co_	MAF_ca_	p_CCA_	p_CCG_	OR (95% CI)	T∶U	OA	p_TDT_
*5p13.1*	rs1992660	G	A	0.99	0.62	0.40	0.31	**0.00050**	**0.0020**	0.64 (0.48–0.86)	1.00	0.77	0.38	0.31	**4.53E-05**	**0.00021**	0.72 (0.60–0.86)	194∶131	A	**0.00050**	0.99	0.77	0.38	0.35	0.055	0.16	0.86 (0.73–1.03)	199∶184	A	0.44
*5p13.1*	rs1992662	C	T	0.97	0.86	0.33	0.25	**0.00047**	**0.0018**	0.60 (0.45–0.80)	1.00	0.43	0.32	0.26	**7.59E-05**	**0.00017**	0.76 (0.63–0.91)	173∶118	T	**0.0013**	0.99	0.43	0.32	0.30	0.22	0.35	0.94 (0.79–1.11)	178∶170	T	0.67
*NELL1*	rs1793004	G	C	0.96	0.97	0.27	0.20	**0.00053**	**0.0020**	0.59 (0.44–0.80)	0.99	0.96	0.28	0.24	**0.025**	0.052	0.85 (0.71–1.00)	135∶104	C	**0.045**	0.99	0.96	0.28	0.23	**0.0017**	**0.0072**	0.77 (0.65–0.92)	167∶156	C	0.54
*NELL1*	rs8176785	G	A	0.91	0.65	0.24	0.26	0.41	0.61	1.16 (0.86–1.57)	0.99	0.02	0.27	0.25	0.12	**0.039**	0.81 (0.68–0.96)	140∶126	A	0.39	0.99	0.02	0.27	0.25	**0.049**	**0.013**	0.79 (0.67–0.94)	149∶142	A	0.68
*NELL1*	rs8176786	T	C	0.92	0.20	0.05	0.05	0.95	0.88	1.01 (0.60–1.70)	1.00	0.72	0.05	0.05	0.63	0.27	0.98 (0.73–1.33)	39∶31	T	0.34	0.99	0.72	0.05	0.05	0.71	0.93	1.06 (0.79–1.41)	52∶51	T	0.92

SNP Information				Panel D (French/Canadian CD)										Panel E (UK CD)						
Locus	dbSNP ID	A1	A2	CR	p_HWE_	MAF_co_	MAF_ca_	p_CCA_	p_CCG_	OR (95% CI)	T∶U	OA	p_TDT_	CR	p_HWE_	MAF_co_	MAF_ca_	p_CCA_	p_CCG_	OR (95% CI)
*5p13.1*	rs1992660	G	A	0.98	0.55	0.43	0.37	**0.0081**	**0.015**	0.65 (0.49–0.87)	178∶163	A	0.42	0.97	0.55	0.40	0.35	**0.036**	0.089	0.82 (0.63–1.06)
*5p13.1*	rs1992662	C	T	0.99	0.37	0.33	0.30	0.16	0.39	0.83 (0.63–1.09)	155∶143	T	0.49	1.00	0.56	0.30	0.37	**0.0011**	**0.00041**	1.23 (0.96–1.59)
*NELL1*	rs1793004	G	C	0.98	0.86	0.24	0.25	0.71	0.86	1.03 (0.78–1.36)	140∶107	C	**0.036**	0.98	0.51	0.26	0.24	0.35	0.63	0.89 (0.69–1.14)
*NELL1*	rs8176785	G	A	1.00	0.60	0.26	0.23	0.12	0.29	0.81 (0.61–1.07)	136∶109	A	0.085	0.97	0.12	0.24	0.22	0.46	0.76	0.91 (0.70–1.18)
*NELL1*	rs8176786	T	C	1.00	0.82	0.06	0.05	0.53	0.48	0.89 (0.57–1.38)	37∶30	T	0.39	0.98	0.03	0.05	0.06	0.43	**0.048**	1.28 (0.86–1.92)

Significant association results (p<0.05) are highlighted by bold type. The SNP callrate (CR) is shown for the combined case/control sample, while the p-value of Hardy-Weinberg proportions (p_HWE_) is shown for controls only. A1 denotes the rare and A2 the more common allele. T∶U is times transmitted vs. times non-transmitted and OA denotes the over-transmitted allele. For further column header descriptions see Table 2.

To corroborate our main association findings, we examined the significantly associated *NELL1* and *5p13.1* SNPs in two additional, independent Caucasian CD samples: Panel D, which comprised population-based Falk-Rubinstein trios from the Québec founder population (QFP), and panel E, a case-control sample from the UK. The *NELL1* association was replicated in the QFP (over-transmission of the common C allele, T∶U = 140∶107, p = 0.036) sample. In addition, the association of *5p13.1* SNP 1992660 was replicated in the QFP case-control sample (p = 0.0081) and the combined p-value for panels B, D, and E was 1.24 × 10^−7^ in an allele-based test. The odds ratio for homozygosity of the common A allele was 1.36 (95% CI: 1.36–2.04). In the UK sample (panel E), the associations of SNPs rs1992660 and rs1992662 were replicated with p-values (allelic χ^2^ test) of 0.036 and 0.0011, respectively, while the *NELL1* SNP association did not achieve formal significance. An overview of the results is given in Tables 5 and 6.

**Table 5:** Summary of association analyses in combined panels. For column header descriptions see Table 4.

SNP Information		Panel A+B (German CD)					Panel A+B+C (German IBD)						Panel B+D+E (CD replication)					
Locus	dbSNP ID	MAF_co_	MAF_ca_	p_CCA_	p_CCG_	OR (95% CI)	MAF_co_	MAF_ca_	p_CCA_	p_CCG_	OR (95% CI)	p_TDT_	MAF_co_	MAF_ca_	p_CCA_	p_CCG_	OR (95% CI)	p_TDT_
*5p13.1*	rs1992660	0.39	0.31	**1.92E-08**	**1.04E-07**	0.70 (0.60–0.81)	0.38	0.33	**5.65E-08**	**3.67E-07**	0.77 (0.68–0.86)	**0.0034**	0.39	0.34	**1.24E-07**	**9.50E-07**	0.74 (0.65–0.84)	**0.0025**
*5p13.1*	rs1992662	0.32	0.26	**1.56E-07**	**8.65E-07**	0.71 (0.61–0.83)	0.32	0.28	**3.25E-06**	**1.28E-05**	0.80 (0.72–0.90)	**0.013**	0.32	0.30	0.076	0.14	0.88 (0.77–1.00)	**0.0058**
*NELL1*	rs1793004	0.27	0.23	**7.81E-05**	**0.00033**	0.77 (0.66–0.89)	0.28	0.23	**4.81E-07**	**2.91E-06**	0.77 (0.69–0.86)	0.076	0.27	0.24	**0.030**	0.074	0.89 (0.78–1.01)	**0.0037**
*NELL1*	rs8176785	0.26	0.25	0.26	0.21	0.89 (0.76–1.03)	0.27	0.25	**0.032**	**0.0048**	0.84 (0.75–0.95)	0.37	0.26	0.24	**0.016**	**0.018**	0.83 (0.73–0.95)	0.070
*NELL1*	rs8176786	0.05	0.05	0.85	0.49	0.99 (0.76–1.29)	0.05	0.05	0.70	0.63	1.02 (0.84–1.24)	0.49	0.05	0.05	0.73	0.90	1.04 (0.84–1.29)	0.20

**Table 6:** Single-point analysis of relevant SNPs in the known CD susceptibility genes *NOD2*, *DLG5*, *IL23R*, and *ATG16L1* carried out in panels A, B, and in the combined panel (A+B).

SNP Information			Screening (Panel A)				Replication (Panel B)				Full (Panel A+B)					
Locus	SNP	dbSNP ID	MAF_co_	MAF_ca_	p_CCA_	p_CCG_	MAF_co_	MAF_ca_	p_CCA_	p_CCG_	MAF_co_	MAF_ca_	p_CCA_	p_CCG_	p_TDT_	OR (95% CI)
***NOD2***	SNP 8/Arg702Trp	rs2066844	0.04	0.11	3.57E-06	5.07E-05	0.05	0.11	6.71E-14	1.63E-12	0.05	0.11	1.29E-18	9.25E-17	3.28E-07	2.48 (1.98–3.10)
***NOD2***	SNP 12/Gly908Arg	rs2066845	0.02	0.04	0.0083	0.0074	0.01	0.04	2.05E-08	1.83E-07	0.01	0.04	7.44E-10	6.08E-09	0.0013	3.04 (2.09–4.42)
***NOD2***	SNP 13/Leu1007fs	rs2066847	0.03	0.16	9.75E-17	4.68E-13	0.04	0.15	1.88E-34	1.13E-28	0.04	0.15	2.51E-49	6.52E-41	3.31E-22	4.30 (3.42–5.42)
***DLG5***	DLG5_e26	-	0.31	0.37	0.018	0.026	0.35	0.36	0.37	0.66	0.34	0.36	0.050	0.12	0.012	1.17 (1.00–1.37)
***DLG5***	Arg30Gln	rs1248696	0.10	0.11	0.59	0.85	0.11	0.10	0.51	0.80	0.11	0.10	0.77	0.94	0.72	0.98 (0.81–1.18)
***DLG5***	Pro1371Gln	rs2289310	0.04	0.04	0.66	0.44	0.03	0.04	0.54	0.72	0.04	0.04	0.77	0.96	0.34	1.04 (0.77–1.41)
***5q31***	IGR2063_b1	-	0.39	0.45	0.039	0.024	0.39	0.48	4.92E-09	1.05E-08	0.39	0.47	1.11E-09	9.21E-09	0.00070	1.49 (1.27–1.76)
***IL23R***	Arg381Gln	rs11209026	0.07	0.03	0.0027	0.0020	0.06	0.03	2.01E-05	9.37E-05	0.06	0.03	2.17E-07	1.04E-06	0.0012	0.49 (0.37–0.65)
***ATG16L1***	Thr300Ala	rs2241880	0.47	0.41	0.014	0.043	0.47	0.41	2.01E-05	9.27E-05	0.47	0.41	8.68E-07	4.02E-06	1.36E-05	0.68 (0.58–0.81)

The SNPs were genotyped in addition to the 100k set to benchmark the results against previously published findings. The SNP coverage of *NOD2*, *DLG5*, *IL23R*, and *ATG16L1* on the 100k array was scarse with 2, 4, 4, and 0 SNPs located in these genes, respectively. The detailed SNP coverage on the array and their relative position to the SNPs retyped here is presented in supplementary Table 7. For a description of column headers, see Table 2.

### Evaluation in Ulcerative Colitis (UC)

The three SNPs rs1793004 (*NELL1*), rs1992660, and rs1992662 (both *5p13.1*) with a confirmed CD association were also analysed in a German UC panel (panel C, 1059 single patients and 419 trios). The *NELL1* SNP rs1793004 also showed a disease association in the UC case-control panel (p = 0.0017 in the allele-based χ^2^ test) and the odds ratio for homozygosity of the common C allele was 1.54 (95% CI: 1.08–2.20 ). Given the similar odds ratio in UC and CD (1.76; 95% CI: 1.27–2.45), *NELL1* appears to be a ubiquitous IBD susceptibility gene (combined p<10^−6^; OR = 1.66, 95% CI: 1.30–2.11). No association to UC was detected for the *5p13.1* locus.

### Fine mapping around NELL1

**Figure 1:**
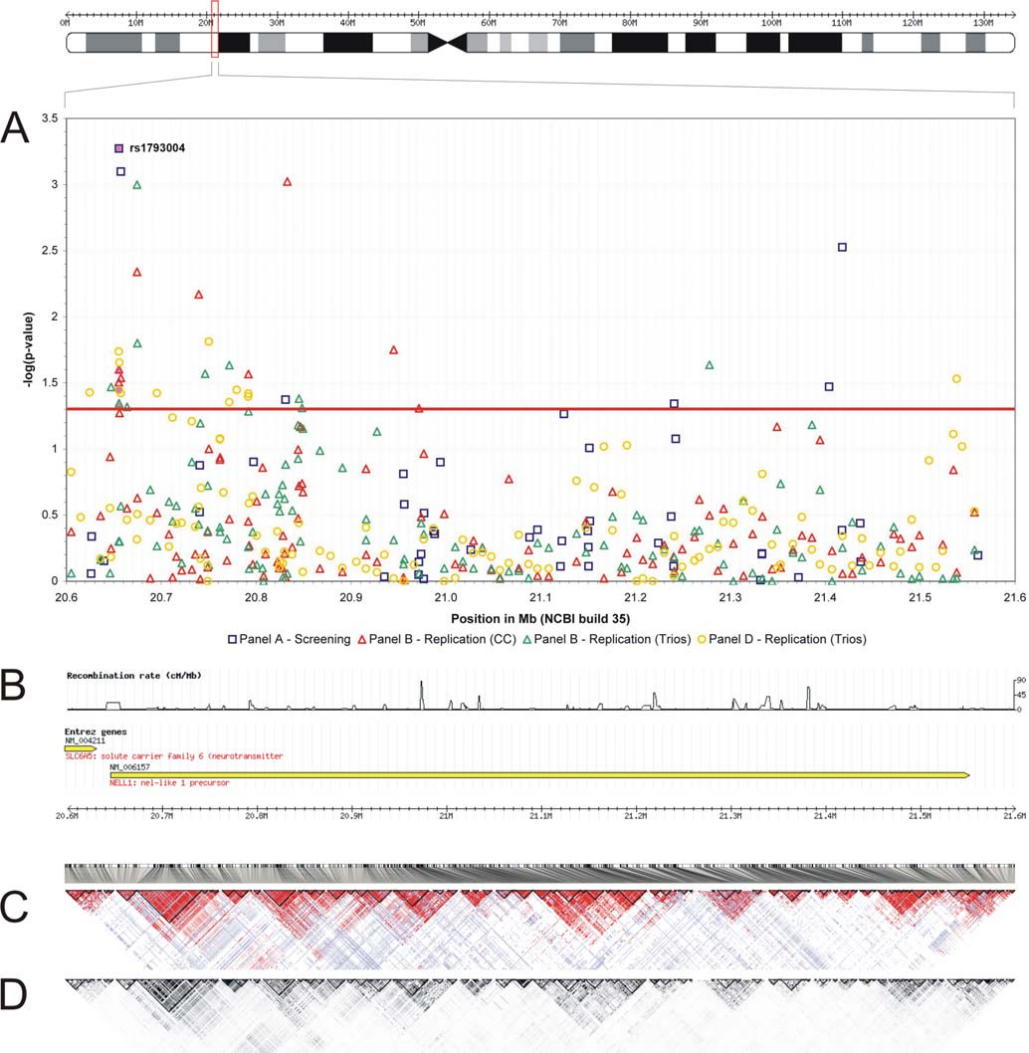
Overview of the CD association findings for the *NELL1* gene. (A) Plot of the negative natural logarithm of the p-values obtained in the different stages of the study across a 1 Mb region. The red line shows the significance threshold (i.e. p = 0.05). Results for SNP rs1793004 are highlighted in pink. The main replication signal localizes to the 5′ region of the *NELL1* gene. (B) Plot of the recombination rate (in cM/Mb), showing that the peak replication signal is delineated by two regions of increased recombination. (C, D) Linkage-disequilibrium (LD) plots from HapMap, using metrics D′ (section C) and r^2^ (section D). Genotypes of trios with Northern- and Western-European ancestry were retrieved from HapMap for 1261 SNPs (CR≥90%, MAF≥1%, p_HWE_≥0.01, Mendel errors≤3). Positions are from NCBI build 35.

Fine mapping around the *NELL1* gene was carried out in replication panels B and D using HapMap tagging SNPs at a density of 8 kb (Figure 1, Table S3). Twenty-one SNPs in the *NELL1* gene yielded a p-value<0.05 in the single-point analyses of panel B (12 in panel D), of which several markers were significant in both, the TDT and case-control test. Results were not corrected for multiple testing because the association between CD and the *NELL1* locus was regarded as established through the previous analyses of panels A and B.

*NELL1* comprises several regions of increased recombination (Figure 1B), scattered over a total of 906 kb. Disease associations were found with various small linkage disequilibrium (LD) blocks, suggesting the existence of more than one causal variant in the gene. In a logistic regression analysis of the combined panels A+B, the best model fit was achieved with SNPs rs1793004, rs951199, rs8176785, rs10500885, rs1158547, and rs1945404. The main association peak was located 5′ of the gene, although a few significant associations were also found towards the 3′ end. The signal sharply declines 5′ of the *NELL1* gene, thereby excluding an involvement of the proximate *SLC6A5* gene. A gender-stratified analysis (data not shown) of the 117 SNPs in panel B confirmed a disease association in both genders.

### Detection of Additional DNA Sequence Variants

Since rs1793004 clearly localizes to *NELL1*, a systematic search for additional, potentially disease-associated variants in the gene was carried out by re-sequencing all exons, splice sites, and the promoter region in 47 CD patients (Table S4; for primer sequences see Table S5). Apart from verifying 26 already annotated variants, five new polymorphisms were identified, two of which represented novel non-synonymous SNPs (nsSNPs): NELL01_02 (R136S) and NELL01_03 (A153T). Both nsSNPs were located in exon 4 and mapped to the thrombospondin N-terminal domain (TSPN) of the NELL1 protein. Two known, common nsSNPs were verified among the 26 annotated SNPs, namely rs8176785 (Q82R) in exon 3 and rs8176786 (R354W) in exon 10. Variant Q82R is located in the TSPN domain, while R354W resides in a von Willebrand factor type C (VWC) domain. *In-silico* analysis, including multiple sequence alignment of NELL homologues and structural modeling of the TSPN domain, revealed a strong conservation of the variant positions (see Text S1 and Figure S4).

The novel nsSNPs were too rare to warrant formal statistical analysis (total occurrence of heterozygotes in panel B (CD/controls): 2/0 for R136S and 10/9 for A153T). While common nsSNP rs8176786 showed a disease association in panel E (p = 0.048), the second common nsSNP, rs8176785, was significantly associated with CD in panel B (p = 0.039), and with UC in panel C (p = 0.013). The combined p-value in the full German IBD sample (A+B+C) was 0.0048 in a genotype-based χ^2^ test (2 degrees of freedom).

### Expression and Localization of NELL1 within the Intestinal Mucosa

**Figure 2:**
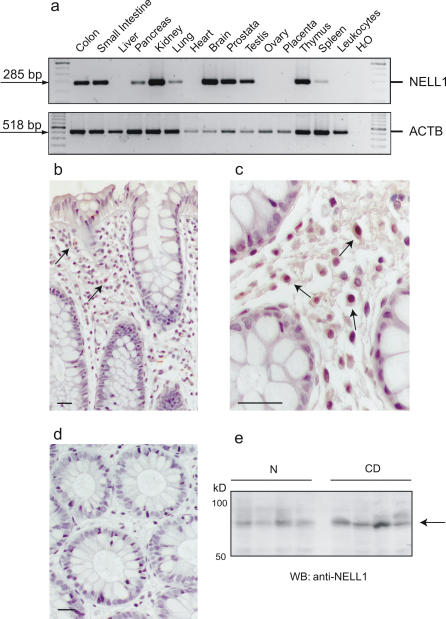
Expression and localization of NELL1. (A) Transcript levels of *NELL1* in a set of different tissues were quantified by RT-PCR. Parallel amplification of β-actin (*ACTB*) is shown. Expression and localization of the NELL1 protein in healthy colonic tissue is demonstrated in sections B (20×) and C (40×; bar = 10 µm) by immunohistochemistry. Immunoreactivity is confined to mononuclear/lymphocytic cells in the lamina propria (brown DAB reaction product, arrows). A control without the primary antibody is shown in section D. No major expression differences between colonic specimen from normal controls (N) and Crohn disease (CD) were detected in the Western blot (section E) with the same antibody as applied in sections B and C. The single detected band (90 kDa) corresponds to the predicted molecular weight of the isoform encoded by GenBank AK127805 (UniProt accession number Q92832).

When *NELL1* transcript levels were investigated by RT-PCR in a tissue panel, high expression became apparent in small intestine, kidney, prostate, and brain, whereas moderate expression was seen in colonic mucosa and in immune-relevant organs/cells such as thymus and spleen (Figure 2A). The localization of NELL1 in the colonic mucosa was investigated by immunohistochemistry (Figures 2B and 2C). Immunoreactivity was confined to large mononuclear cells in the lamina propria. In Western blot experiments using colonic biopsy specimen from normal controls and CD patients (Figure 2E), the antibody recognized a single 90 kDa band corresponding to the correct size of the annotated NELL1 transcript (AK127805). Real-time quantitative PCR revealed no significant difference between normal and patient tissue (data not shown).

### Fine Mapping of 5p13.1

The 650 kb susceptibility region on *5p13.1*, upstream of the *PTGER4* gene, was subjected to fine mapping in panels B (1 SNP/24 kb) and D (1 SNP/3 kb). Several SNPs showed a consistent disease association in both panels (Table S6, Figure S5). The strongest effect in the combined case-control panel (A+B) was seen for SNP rs1553575 (odds ratio for homozygotes of the common G allele: 1.78; 95% CI: 1.32–2.40).

Interestingly, the gender-stratified analysis of *5p13.1* SNPs showed that the association signal was stronger in females than in males (Table S7), suggesting that females carrying the predisposing allele(s) of this locus are at higher risk to develop Crohn disease than their male counterparts. To have comparable power, the same number of male and female individuals were randomly drawn from the combined panel (378 controls, 343 cases).

### Interaction with Known Disease Loci

Logistic regression analysis and a Breslow-Day test for odds ratio heterogeneity were used to analyse the full German case-control panel (A+B) for potential epistatic effects. No statistically significant interactions were observed, neither between polymorphisms within the *NELL1* gene (rs1793004) or the *5p13.1* region (rs1992660 and rs1992662), nor between these loci and any of the known disease-associated variants in *IL23R* (rs11209026/Arg381Gln), *NOD2* (rs2066844/Arg702Trp, rs2066845/Gly908Arg, rs2066847/Leu1007fs), *ATG16L1* (rs2241880/T300A), *DLG5* (rs1248696/Arg30Gln), or in the *IBD5* region (tagging SNP IGR2063_b1 [11], [29]).

## Discussion

We have identified *NELL1* as a novel disease gene for Crohn disease (CD), a result that was obtained in a genome-wide case-control association scan with 116,161 SNPs and by extensive replication in three independent samples from three distinct ethnicities. In a recently published GWAS from the UK population [30], [31] (1,748 CD patients and 2,938 controls genotyped), the *NELL1* region was covered with 263 SNPs (see http://www.wtccc.org.uk/). Of these, 23 SNPs were significantly associated with CD (p<0.05 under an additive or general model) and six SNPs had a p<0.01: rs7122630, rs4475916, rs7115151, rs11025862, rs2063913, rs11026037. The *NELL1* region was not subjected to replication in the UK scan, since none of the 23 SNPs fell below the chosen cut-off (p<010^−5^).

In addition to identifying *NELL1* as a CD risk factor, we also replicated the disease association recently described for the *5p13.1* region [18]. The genome-wide scan also confirmed two of the previously known CD loci, *NOD2* and *5q31*, but it should be pointed out that the marker set only covered 31% of the genome [32], [33]. As has been detailed in Table 4, the previously established disease association of neither *IL23R* [17], nor *DLG5* [12], nor *ATG16L1* [15], were detected. However, not a single SNP, for example, in the *ATG16L1* gene was present on the Affymetrix GeneChip® Human Mapping 100K Set (Table S8) and coverage of all these genes was low. Targeted *post-hoc* genotyping of the relevant SNPs in the German screening and replication panel was therefore carried out and confirmed the CD associations of *ATG16L1*, *IL23R* and *DLG5* in our study sample (Table 4). We replicated the association of a haplotype A-tagging SNP in *DLG5* which is supported by several other replications of the association between *DLG5* and CD association [34]–[42]. Results for the two nonsynonymous SNPs in *DLG5* were not reaching significance. However, as it has been speculated in our original description [12], we do not consider these SNPs as causative at this point. They are either part of a larger number of putatively causative SNPs not yet discovered or mere additional markers for unknown causative variants. We expect further relevant, hitherto unknown and rare variants in *DLG5* that may only be detectable by large-scale sequencing of the gene [43]. It should also be noted that the *DLG5* association has not been replicated in each and every population analysed so far [44]–[46]. Recent studies have proposed gender- and/or age at onset-related associations of *DLG5* [13], [34], [40]–[42] that would require exact matching of the study groups to become detectable. Our sample used in this study contained mainly CD patients with disease onset during early adulthood (average age at onset>22 years). This may have contributed to a replication that was weaker than the original description in statistical terms (for review see [47]).

The targeted replication of the CD association of *ATG16L1*, *IL23R* and *DLG5* also serves to illustrate the highly conservative criteria employed in our study, which may have resulted in an under-appreciation of most initial association findings. Using these criteria, *ATG16L1*/Thr300Ala and *IL23R*/Arg381Gln would not have been included in the follow-up because the p-values of the two variants (0.014 for Thr300Ala and 0.0027 for Arg381Gln) both exceeded the cut-off of 0.0021 (attained by rs3790889 as number 200 of the ranked SNP list). Therefore, future efforts to replicate the major findings of our study should also include those SNPs that yielded a significant p-value in only one of the replication panels.

**Figure 3:**
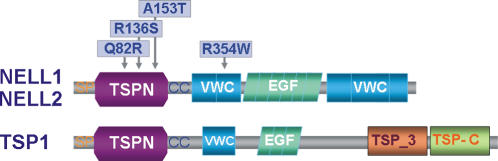
* In silico* protein analysis. Domain architectures of NELL1/NELL2 and TSP1. The positions of variant amino acids are annotated. Abbreviations are as follows: SP: signal peptide; TSPN: thrombospondin N-terminal domain; CC: coiled-coil region; VWC: von Willebrand factor, type C domain; EGF: EGF domain; TSP-3: thrombospondin-3 repeat; TSP_C: thrombospondin C-terminal domain.

The *neural epidermal growth-factor-like (nel)* gene was first detected in neural tissue from an embryonic chicken cDNA library, and its human orthologue *NELL1* was later discovered in B-cells [48]–[50]. The arrangement of the functional domains of the 810 aa protein bears resemblance to thrombospondin-1 (TSP-1) and consists of a thrombospondin N-terminal domain (TSPN) and several von Willebrand factor, type C (VWC), and epidermal growth-factor (EGF) domains [51]. As NELL1 binds to, and is phosphorylated by, PKC-β1 via the EGF domains [52], it has been suggested that this protein belongs to a novel class of cell-signalling ligand molecules critical for growth and development. Re-sequencing and fine mapping revealed several non-synonymous SNPs of which the known Q82R variant and the novel R136S and A153T variants affect the TSPN domain, while R354W is located in a VWC domain (Figure 3) [51]. A153T is close to two highly conserved C-terminal cysteines forming a disulfide bond in the TSPN domain structure of TSP-1 [53] and may cause local conformational changes due to its buried position in the molecule. Generally, the TSPN domain has been shown to serve as a protein-protein interaction module, which binds membrane proteins and proteoglycans and exhibits versatile cell-specific effects on adhesion, migration, and proliferation [54], [55]. Since VWC domains occur in numerous proteins of diverse functions and are generally assumed to be involved in protein oligomerization [56], R354W may interfere with NELL1 trimerization [51].

Bone development is severely disturbed in transgenic mice, where over-expression of *NELL1* leads to craniosynostis [57] and *NELL1* deficiency manifests in skeletal defects due to reduced chondro- and osteogenesis [58]. Interestingly, osteopenia and osteoporosis are leading co-morbidities in IBD patients, even without the use of glucocorticoids [59]–[61]. *PTGER4*, which has been suggested as the causative gene in the *5p13.1* locus, is among the key genes that are down-regulated in *NELL1*-deficient mice [58]. However, no statistical interaction was seen in our study between the *NELL1* and *5p13.1* SNPs.

The replication criteria used in our study were particularly strict and required a significant p-value in both, the family-based and the case-control association test in two different populations. Other studies used less stringent criteria for the replication of genome-wide association findings, and based their conclusions upon a single independent case control sample only [14], [17]. With such criteria, several additional SNPs would have been considered replicated in our study, some of which point towards genes putatively involved in the pathophysiology of IBD. Integrin beta 6 (*ITGB6*), for example, regulates activation of TGF-β [62], a cytokine that has been established as an anti-inflammatory regulator in TNF-related CD pathopysiology [63], [64]. Two hits point towards the glutamate pathway, namely glutamate receptor type 8 (*GRM8*) and glutamate receptor, ionotropic, N-methyl-D-aspartate 3A (*GRIN3A*, formerly *PPP3R2*). Normal glutamate metabolism has been found to be important for the maintenance of intestinal function. Finally, SNP rs7868736 is located approximately 100 kb upstream of the *ZNF618* gene encoding a zinc finger protein clearly expressed in human colon.

In summary, we have successfully performed a systematic genome-wide association scan in Crohn disease that led to the identification of the *NELL1* gene on chromosome *11p15.1* as a novel susceptibility gene for IBD. We confirmed *5p13.1* as a CD-associated locus relating to *PTGER4* that is probably regulated by *NELL1*.

## Methods

### Patient Recruitment

German patients and controls in panels A, B, and C partially overlap with samples that have been used in other studies before [12], [15], [65], [66]. Panels A and B almost completely overlap with the panels (also termed panel A and B) that were used in a recently published IBD association screen of non-synonymous SNPs [15]. In this non-synonymous SNP scan no coding SNPs that were evaluable were located in *NELL1*. All patients were recruited at the Charité University Hospital (Berlin, Germany) and the Department of General Internal Medicine of the Christian-Albrechts-University (Kiel, Germany), with the support of the German Crohn and Colitis Foundation (Table 1). Clinical, radiological and endoscopic (i.e. type and distribution of lesions) examinations were required to unequivocally confirm the diagnosis of Crohn disease or ulcerative colitis [67], [68], and histological findings also had to be confirmative of, or compatible with, the diagnosis. In the case of uncertainty, patients were excluded from the study. German control individuals were obtained from the POPGEN biobank [25].

The UK patients (Panel E) were recruited as described before [69]; UK controls were obtained from the 1958 British Birth Cohort (http://www.b58cgene.sgul.ac.uk).

The French/Canadian trios (Panel D) were sampled from the Québec founder population (QFP). Membership of the founder population was defined as having four grandparents with French Canadian family names who were born in the Province of Quebec, Canada, or in adjacent areas of the Provinces of New Brunswick and Ontario, or in New England or New York State (USA).

Informed written consent was obtained from all study participants. All collection protocols were approved by the institutional review committees of the participating centres.

### SNP Genotyping with the Affymetrix 100k Gene Chip Array

Genotyping of cases and controls was carried out using the Affymetrix GeneChip® Human Mapping 50K Xba and Hind Arrays (Affymetrix, Santa Clara, CA, USA). Genotypes were called by the GeneChip® DNA Analysis Software (GDAS v2.0, Affymetrix). Gender was verified by counting the heterozygous SNPs on the X chromosome. Quality checks further comprised the verification of individual sample call rates (≥90%) and, to ensure that no samples were confused, the 31 identical SNPs present on both chips were checked for identical genotypes for the same individual. SNPs that had a low genotyping success rate (<90%), were monomorphic, or deviated from Hardy-Weinberg equilibrium (p≤0.01) were eliminated from subsequent analyses. Experimental details concerning the genotyping of the 100k SNP set are provided in Matsuzaki *et al.* [22].

### Follow-up Genotyping and Sequencing

SNPlex™ (Applied Biosystems, Foster City, CA, USA) genotyping of panels A, B, and C was carried out as recently described [15]. Genotype concordance rates for SNPs rs1793004, rs1992660, and rs1992662 were checked using TaqMan (Applied Biosystems) as an independent genotyping technology on an automated platform [70] and the functionally tested assays C____392093_10, C__11472026_10, and C__11472042_10. All three concordance rates were >98%, excluding genotyping errors as a potential source of false-positive associations. The same three TaqMan assays were also used to genotype panels C and E. Genotypes for panel D were generated at Genizon BioSciences using the Illumina GoldenGate™ platform (Illumina, San Diego, CA, USA). All process data were logged into, and administered by, a database-driven LIMS [71]. TaqMan genotyping of *NOD2*/Arg702Trp, *NOD2*/Gly908Arg, *NOD2*/Leu1007fs, *DLG5*/Arg30Gln, *DLG5*/Pro1371Gln, *DLG5*/e26, and *ATG16L1*/Thr300Ala was performed using previously described assays [12], [15], [72]. *IL23R*/Arg381Gln, *NELL1*/rs8176785, and *NELL1*/rs8176786 were genotyped in panels A, B, C, and E using the functionally tested assay C___1272298_10, C___3203197_10, and C__32647553_10, respectively (Applied Biosystems). Prior to statistical analyses, the same cut-off criteria as described above for the 100k analysis (p_HWE_>0.01, MAF_controls_>0, callrate≥90%) were applied to the SNPs under study.

Sequencing of genomic DNA was performed using Applied Biosystems BigDye™ chemistry according to the supplier's recommendations (for primer sequences, see supplementary Table S1). Traces were inspected for the presence of SNPs and InDels using novoSNP [73].

### Statistical Analysis

Genome-wide data analysis was carried out using an updated version of GENOMIZER [74]. Association hits that passed the quality criteria were extracted using the “GenomizerHits” tool (http://www.ikmb.uni-kiel.de/genomizer/). Haploview 4.0 [75] was used for association analysis, transmission disequilibrium tests, and LD quantification of the replication data. Fisher's exact test was used when appropriate. The supplementary p-value plots (Figure S7) and quantile-quantile plots (Figure S3) were created using R (http://www.r-project.org/). Single-marker disease associations and possible marker-marker interactions were assessed for statistical significance by means of logistic regression analysis (forward selection), as implemented in the procedure LOGISTIC of the SAS software package (SAS Institute, Cary NC, USA). Haplotype analyses were carried out using COCAPHASE 2.403 [76] and PHASE 2.1 [77], [78].

### RT-PCR, Western Blot and Immunohistochemistry

For the assessment of tissue-specific expression patterns, a commercial tissue panel was employed (Clontech, Palo Alto, CA, USA). Primers used for amplification of *NELL1* were (NELL1_14-16_F ACCTTCCTGGGTTATATCGCTGTG and NELL1_14-16_R TCTCGCAGTGGCTTCCTGTG, expected amplicon length: 285 bp). The following conditions were applied: denaturation for 5 min at 95°C; 40 cycles of 30 sec at 95°C, 20 sec at 60°C, 45 sec at 72°C; final extension for 10 min at 72°C. To confirm the use of equal amounts of RNA in each experiment, all samples were checked in parallel for β-actin mRNA expression. All amplified DNA fragments were analyzed on 2% agarose gels and subsequently documented by a BioDoc Analyzer (Biometra, Göttingen, Germany).

Paraformaldehyde-fixed paraffin-embedded biopsies from normal controls (n = 6) and from patients with confirmed colonic CD (n = 6) were analysed. Two slides of each biopsy were stained with hematoxylin-eosin for routine histological evaluation. The other slides were subjected to a citrate-based antigen retrieval procedure, permeabilized by incubation with 0.1% Triton X-100 in 0.1M phosphate-buffered saline (PBS), washed three times in PBS and blocked with 0.75% bovine serum albumin in PBS for 20 minutes. Sections were subsequently incubated with the primary antibody (anti-NELL1, Abnova , mouse monoclonal) at a 1∶500 dilution in 0.75% BSA overnight at 4°C. After washing in PBS, tissue-bound antibody was detected using biotinylated goat-anti mouse (Vector Laboratory, Burlingame, CA) followed by HRP-conjugated avidin, both diluted at 1∶100 in PBS. Controls were included using irrelevant primary antibodies as well as omitting the primary antibodies using only secondary antibodies and/or HRP-conjugated avidin. No significant staining was observed with any of these controls (data not shown). Bound antibody was detected by standard chromogen technique (Vector Laboratory) and visualized by an Axiophot microscope (Zeiss, Jena, Germany). Pictures were captured by a digital camera system (Axiocam, Zeiss).

Western blot analysis was performed as described [79]. In brief, 20 µg of protein lysates freshly derived from colonic biopsies of four healthy controls without any obvious intestinal pathology and four CD patients with confirmed ileal and colonic inflammation were lysed, separated by SDS polyacrylamide gel electrophoresis and transferred to PVDF membrane by standard techniques. NELL1 was detected using the same monoclonal anti-NELL1 antibody also employed for immunohistochemistry.

### In silico Protein Analysis

**Figure 4:**
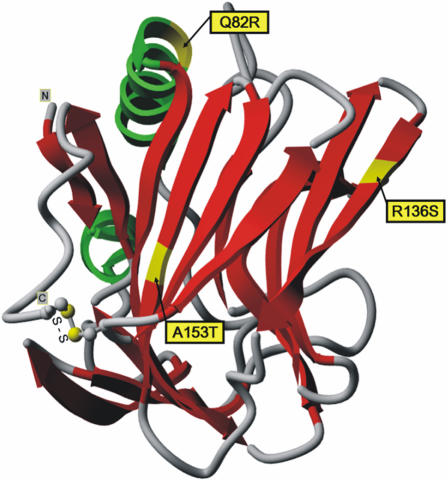
* In silico* protein analysis. Computationally derived 3D structure model of the N-terminal domain of the NELL1 protein. The model was created using the TSPN (PDB code 1z78, chain A) as a structure template for NELL1. The locations of variant amino acids as well as of two cysteines forming a disulfide bridge are annotated.

Aligned sequences (Figures S4A and S4B) were retrieved from the UniProt database (http://www.uniprot.org) and protein domain architectures taken from the NCBI conserved domain search website (http://www.ncbi.nlm.nih.gov:80/Structure/cdd/wrpsb.cgi). To predict the 3D structure of the N-terminal domain of NELL1, we explored the fold recognition results returned by the web servers GenTHREADER (http://bioinf.cs.ucl.ac.uk/psipred/) and FFAS03 (http://ffas.ljcrf.edu). Based upon the very similar server predictions, a pair-wise sequence-structure alignment of NELL1 to the crystal structure of the human thrombospondin-1 N-terminal domain (TSPN) was constructed as input for the 3D-modeling server WHATIF (http://swift.cmbi.kun.nl/WIWWWI/), which returned a structure model of the NELL1 N-terminal domain (Figure 4). Additional details are given in the supplements (Text S1).

## Supporting Information

Text S1 Supplementary methods, results, and discussion - Protein *in silico* analysis.
(0.58 MB PDF)

Figure S1 Workflow diagram summarizing the different stages of the experiment.
(0.22 MB PDF)

Figure S2 Power calculation for the present genome-wide association scan of 393 cases and 399 controls (a = 0.05). Five different allele frequencies in controls (p0) are shown: 50%, 30%, 20%, 10%, and 5%. The scan had e.g. 80% power to detect an Odds ratio of 1.6 assuming that 20% of the controls are exposed. Calculations were done using the software PS Power and Sample Size Calculations.
(0.15 MB PDF)

Figure S3 Quantile-quantile plots (A) before and (B) after exclusion of lowquality SNPs (callrate<90%, pHWE<0.01, MAF>0.99). The ranked, observed Chi2-values of the allelic test are plotted against the values expected for sampling from a Chi2 distribution with one degree of freedom (the distribution expected under the null hypothesis). The inflation of the Chi2 distribution is significantly reduced by removing low-quality SNPs and there is no visible indication of population structure. The outlier in the upper right corner of the diagram B represents genuine association of SNP rs2076756 in the NOD2 gene with CD (see Table S2, Lead #1).
(0.22 MB PDF)

Figure S4 (A) Structure-based multiple sequence alignment of the N-terminal domains of NELL1 and NELL2 homologs and the N-terminal domain of human thrombospondin-1 (TSP-1). The DSSP secondary structure assignment of the TSPN structure (PDB code 1z78, chain A) is depicted at the top of the alignment. (B) Multiple sequence alignment of NELL1 and NELL2 homologs. Domain locations are represented as colored bars at the top of the alignment (green: VWC domain; pink: EGF-like domain). Alignment columns with more than 70% physicochemically similar amino acids are highlighted in blue boxes with white letters. Text labels point to the N-terminal signal peptide and the sequence variants, which are marked yellow in all homologs. Residue numbering in the alignment is based on complete protein sequences as derived from corresponding UniProt entries.
(0.41 MB PDF)

Figure S5 Overview of the results for the 5p13.1 locus. (A) Plot of the negative common logarithm of the p-values of the different tiers across the 650 kb region. The red line shows the significance threshold of p = 0.05. Results of the two lead SNPs rs1992662 and rs1992660 are highlighted in pink color. The broad replicated peak between 40.29 Mb and 40.66 Mb localizes to a gene desert upstream of PTGER4. (B) Recombination rate in cM/Mb shows that the peak region is delineated by two sites of increased recombination. (C) Linkage-disequilibrium (LD) plot from HapMap, using the metrics D′ and (D) r2. Genotypes of trios with northern and western European ancestry for 633 SNPs (CR> = 90%, MAF> = 1%, pHWE> = 0.01, Mendel errors> = 3) were retrieved from HapMap. Positions are from NCBI build 35.
(1.36 MB PDF)

Figure S6 Secondary structure prediction for human NELL1 (UniProt accession number Q92832). The PSIPRED web server produced the depicted prediction.
(0.13 MB PDF)

Figure S7 The diagrams on the first page show the results of the whole genome association scan for Crohn disease and subsequent pages show the enlarged diagrams for each chromosome. The negative common logarithm of the p-values for the allelic test are shown. Only markers that passed the quality criteria listed in Table S1 were used for plotting (n = 92,387). “Outlier” SNP rs2076756 in the CARD15 gene (pCCA<10–12, 50.53 Mb) was omitted for illustration purposes. Marker positions are from NCBI build 34.
(11.06 MB PDF)

Table S1 Summary of the genome-wide association scan. Table (A) gives an overview of the SNP number and density per chromosome. Table (B) lists the distribution of the p-values in the genome-wide scan versus a randomized experiment, i.e. randomized affection status of individuals. CR: callrate in cases and controls, MAF: minor allele frequency in controls, pHWE: p-value for Hardy-Weinberg equilibrium in controls, QC: quality control, CCA: p-value of Chi2 test for alleles and genotypes (CCG), *excl. unmapped
(0.12 MB PDF)

Table S2 Top 200 CD-associated SNPs, ranked with respect to p-values obtained in an allele- (pCCA) or genotype-based (pCCG) case-control comparison in panel A. Also included are pCCA, pCCG, and the transmission disequilibrium test results (pTDT) for the replication panel B. Nucleotide positions refer to NCBI build 35. Markers with p< = 0.05 in either the case-control analysis or the transmission disequilibrium test (TDT) in replication panel B are highlighted in bold italics. SNPs with a significant result in both panel B tests are additionally marked by grey shading.
(0.15 MB PDF)

Table S3 Fine mapping of the CD association signal at the NELL1 locus in replication panels B and D. The p-values of the allele-based (pCCA) and genotype-based (pCCG) association analyses of the tagging SNPs are shown, pTDT is the p-value for the transmission disequilibrium test (TDT). Lead SNPs from the initial screening (see Table S2) are highlighted by grey shading, nonsynonymous SNPs in red color. Polymorphisms that are significant in either the TDT or the case-control analyses, are highlighted in bold italics and those significant in both are highlighted in blue color. Pairwise LD is listed using the metric r2 as calculated with Haploview [1] and minor allele frequencies (MAF) are listed for control individuals. Nucleotide positions refer to NCBI build 35.
(0.14 MB PDF)

Table S4 Summary of the mutation detection of NELL1. All 21 exons plus the promoter were resequenced in 47 unrelated Crohn disease patients. Five not yet annotated polymorphisms were identified, including the two nonsynonymous SNPs NELL1_02 and NELL1_03. Twenty-sex annotated SNPs were verified.
(0.14 MB PDF)

Table S5 Primer sequences used for the mutation detection of NELL1.
(0.13 MB DOC)

Table S6 Fine mapping of the CD association signal at the 5p13.1 locus in replication panels B and D. The highlighting and the column headers are the same as described in Table Legend S3.
(0.16 MB PDF)

Table S7 Results of gender-stratified analysis for 5p13.1 SNPs. Only markers that showed a significant p-value in the single-point analysis of the two subgroups are shown. 378 controls and 343 CD cases were randomly drawn from each subgroup and odds ratios (OR) for carriership of the common allele are shown.
(0.18 MB PDF)

Table S8 SNP coverage of 100k Array for known IBD susceptibility loci. Variants of Table 4 were included (highlighted by grey shading) to calculate distances. Nucleotide positions refer to NCBI build 35 and the following gene regions were used: IL23R: 67002085-67095564, ATG16L1: 234447031-234491134, DLG5: 78895154-79030951, NOD2: 50509083-50545020.
(0.16 MB PDF)
