# Supplementary material for: Systematic Association Mapping Identifies NELL1 as a Novel IBD Disease Gene
Source: PLoS One. 2007 Aug 8;2(8):e691. doi: 10.1371/journal.pone.0000691 (PMC1933598; doi:10.1371/journal.pone.0000691)
Supplement: Text S1 — Supplementary methods, results, and discussion - Protein in silico analysis. (0.58 MB PDF) [file pone.0000691.s001.pdf]

SUPPLEMENTARY METHODS - *in silico* protein analysis

We used the PSI-BLAST program (http://www.ncbi.nlm.nih.gov/BLAST/) to search the Protein Data Bank (PDB, http://www.rcsb.org/pdb/) and the NCBI non-redundant (NR) database for NELL1 homologs. Protein domain architectures were retrieved from the databases Pfam (http://www.sanger.ac.uk/Software/Pfam) and SMART (http://smart.embl.de/) and examined through the NCBI conserved domain search website (http://www.ncbi.nlm.nih.gov:80/Structure/cdd/wrpsb.cgi).

Using MUSCLE (http://phylogenomics.berkeley.edu/cgi-bin/muscle/input_muscle.py), we created a multiple sequence alignment of the following protein sequences from the UniProt database:

NELL1: human, Q92832; mouse, Q2VWQ2; rat, Q62919;

NELL2: human, Q99435; mouse, Q61220; rat, Q62918; frog, Q7ZXL5;

NEL chicken, Q90827; TSP1 human, P07996 (PDB identifier 1z78, chain A).

The secondary structure assignment of the PDB structure 1z78 was obtained from the DSSP database (http://www.cmbi.kun.nl/gv/dssp/) and added to the alignment. To predict the secondary structure of NELL1, we contacted the protein structure prediction server PSIPRED (http://bioinf.cs.ucl.ac.uk/psipred/). The cleavage site of the N-terminal signal peptide in NELL1 was predicted by the SignalP server (http://www.cbs.dtu.dk/services/SignalP/). The sequence-structure alignment figure was prepared using GeneDoc (http://www.psc.edu/biomed/genedoc/).

To predict the 3D structure of the NELL1 gene product, we used the fold recognition servers GenTHREADER (http://bioinf.cs.ucl.ac.uk/psipred/) and FFAS03 (http://ffas.ljcrf.edu). In agreement with the consistent results returned by PSI-BLAST, GenTHREADER, and FFAS03, we selected the thrombospondin-1 (TSP1) N-terminal domain (TSPN, PDB code 1z78, chain A) to model the 3D structure of NELL1. The structure of TSPN comprises the residues 28-233 (UniProt sequence P07996).

Based upon the 3D prediction results, a pairwise sequence-structure alignment of human NELL1 and TSPN was constructed and formed the input into the 3D-modeling server WHAT IF (http://swift.cmbi.kun.nl/WIWWWI/), which returned a full-atom structure model of the N-terminal domain of NELL1. The protein structure image of the model was illustrated using Yasara (http://www.yasara.org) and POV-Ray (http://www.povray.org).

SUPPLEMENTARY RESULTS AND DISCUSSION – *in silico* protein analysis

Multiple sequence alignment of NELL homologs

Based on the consistent and very reliable results returned by the alignment methods PSI-BLAST, Pfam, SMART, GenTHREADER and FFAS03, we assembled a structure-based multiple sequence alignment (Figure S4) of the N-terminal domain of NELL homologs and the thrombospondin-1 (TSP1) N-terminal domain (TSPN). The secondary structure prediction by PSIPRED agreed well with the predicted 3D structure of NELL1 (Figure S6). TSP1 is a secreted protein that binds to a variety of extracellular matrix components and proteins involved in the regulation of cell growth and inflammatory response[2,3].

The domain composition of TSP1 bears striking resemblance to NELL proteins, which suggests functional similarities between TSP1 and NELL1 and NELL2 (Kuroda BBRC_1999, PMID 10548494). Domains in common include a well-conserved N-terminal TSPN domain connected via a coiled coil region to von Willebrand factor, type C domains (VWC), which are followed by epidermal growth factor (EGF) domains (Figure 4A). The N-terminal domains of NELL1 and TSPN share 20% identical amino acids. The equivalent TSPN cysteines of the two NELL1 cysteines C167 and C220 form a disulfide bond in TSPN and stabilize a C-terminal helix[4].

Interestingly, the resulting arginine of the NELL1 variant Q82R is identical in all NELL homologs and TSP-1 except human NELL1, which has the glutamine. The original arginine

and alanine of the two variants R136S and A153T, respectively, are strictly conserved in all NELL1 orthologs, but not in NELL2 orthologs and TSPN (Figure S4A and S4B). R136 is replaced by a histidine in human, mouse, rat NELL2, TSPN, and by a glutamine in frog NELL2. The amino acid change R136S could not be observed in any homologous sequence. While A153 is replaced by a serine in NELL2 orthologs, it is a threonine in TSPN. A threonine is also found in other remotely related proteins at equivalent positions, for instance, in the laminin G-like domains of Gas6[5] and in LAMA2[6]. The variant R354W is located outside the TSPN domain within a VWC domain predicted by Pfam (identifier PF00093) and SMART (identifier SM00214). As shown in Figure S4B, the arginine R354 is identical in all NELL1 homologs, but changed to a serine in NELL2 homologs. Since VWC domains occur in numerous proteins of diverse functions and are generally assumed to be involved in protein oligomerization[7], the variant R354W may interfere with NELL1 trimerization[8].

3D structure model

We investigated the effects of the variants Q82R, R136S, and A153T on the structure of the predicted NELL1 N-terminal TSP domain *in silico*. To this end, we extracted a pairwise sequence-structure alignment of human NELL1 to the crystal structure of the human thrombospondin-1 N-terminal domain (TSPN) from the multiple sequence alignment (Figure S4A) and applied the WHAT IF server to construct a 3D model of the N-terminal domain of NELL1 using the PDB structure of TSPN (PDB code 1z78, chain A) as template (Figure 2B). The TSPN structure is a β-sandwich with 13 anti-parallel β-strands, adopting the concanavalin A-like lectins/glucanases fold[4]. Many amino acids of the hydrophobic core are conserved between TSPN and NELL1, including two cysteines forming a disulfide bond in the TSPN structure.

The variant Q82R changing a neutral glutamine to a positively charged arginine is located outside the β-sandwich in an α-helix. The respective arginine in the TSPN domain is part of a

small patch of positive charge on the surface of the protein in a hypothetical heparin-binding site[4]. The variant R136S changes a positively charged arginine to a polar serine on the surface of the protein. The corresponding position 139 in the TSPN domain is also a polar histidine. As in case of Q82R, charge changes on the surface of a domain may lead to the loss of molecular interactions.

Variant A153T is located close to the two C-terminal cysteines forming a disulfide bond in the TSPN structure. The structural role of this bond is the stabilization of an α-helix located at the C-terminus of the domain[4]. NELL homologs lack four amino acids in this helical region, and the secondary structure prediction of NELL1 indicates a loop. Based on the conserved cysteines and our close inspection of the atomic distances in this region, we propose a substitution of the TSPN helix with a loop structure, which buries roughly the same amino acids as observed in TSPN. Presumably, the variant A153T is located opposite the proposed loop and thus may be buried.

SUPPLEMENTARY REFERENCES

1. Barrett JC, Fry B, Maller J, Daly MJ (2005) Haploview: analysis and visualization of LD and haplotype maps. Bioinformatics 21: 263-265.
2. Bornstein P (1995) Diversity of function is inherent in matricellular proteins: an appraisal of thrombospondin 1. J Cell Biol 130: 503-506.
3. Adams JC, Lawler J (2004) The thrombospondins. Int J Biochem Cell Biol 36: 961-968.
4. Tan K, Duquette M, Liu JH, Zhang R, Joachimiak A, et al. (2006) The structures of the thrombospondin-1 N-terminal domain and its complex with a synthetic pentameric heparin. Structure 14: 33-42.
5. Sasaki T, Knyazev PG, Cheburkin Y, Gohring W, Tisi D, et al. (2002) Crystal structure of a C-terminal fragment of growth arrest-specific protein Gas6. Receptor tyrosine kinase activation by laminin G-like domains. J Biol Chem 277: 44164-44170.
6. Hohenester E, Tisi D, Talts JF, Timpl R (1999) The crystal structure of a laminin G-like module reveals the molecular basis of alpha-dystroglycan binding to laminins, perlecan, and agrin. Mol Cell 4: 783-792.
7. Voorberg J, Fontijn R, Calafat J, Janssen H, van Mourik JA, et al. (1991) Assembly and routing of von Willebrand factor variants: the requirements for disulfide-linked dimerization reside within the carboxy-terminal 151 amino acids. J Cell Biol 113: 195-205.
8. Kuroda S, Oyasu M, Kawakami M, Kanayama N, Tanizawa K, et al. (1999) Biochemical characterization and expression analysis of neural thrombospondin-1-like proteins NELL1 and NELL2. Biochem Biophys Res Commun 265: 79-86.
