# Supplementary figures and images for: Systematic Association Mapping Identifies NELL1 as a Novel IBD Disease Gene
Source: PLoS One. 2007 Aug 8;2(8):e691. doi: 10.1371/journal.pone.0000691 (PMC1933598; doi:10.1371/journal.pone.0000691)

**Supplementary Figure 1:** Workflow diagram summarizing the different stages of the experiment.

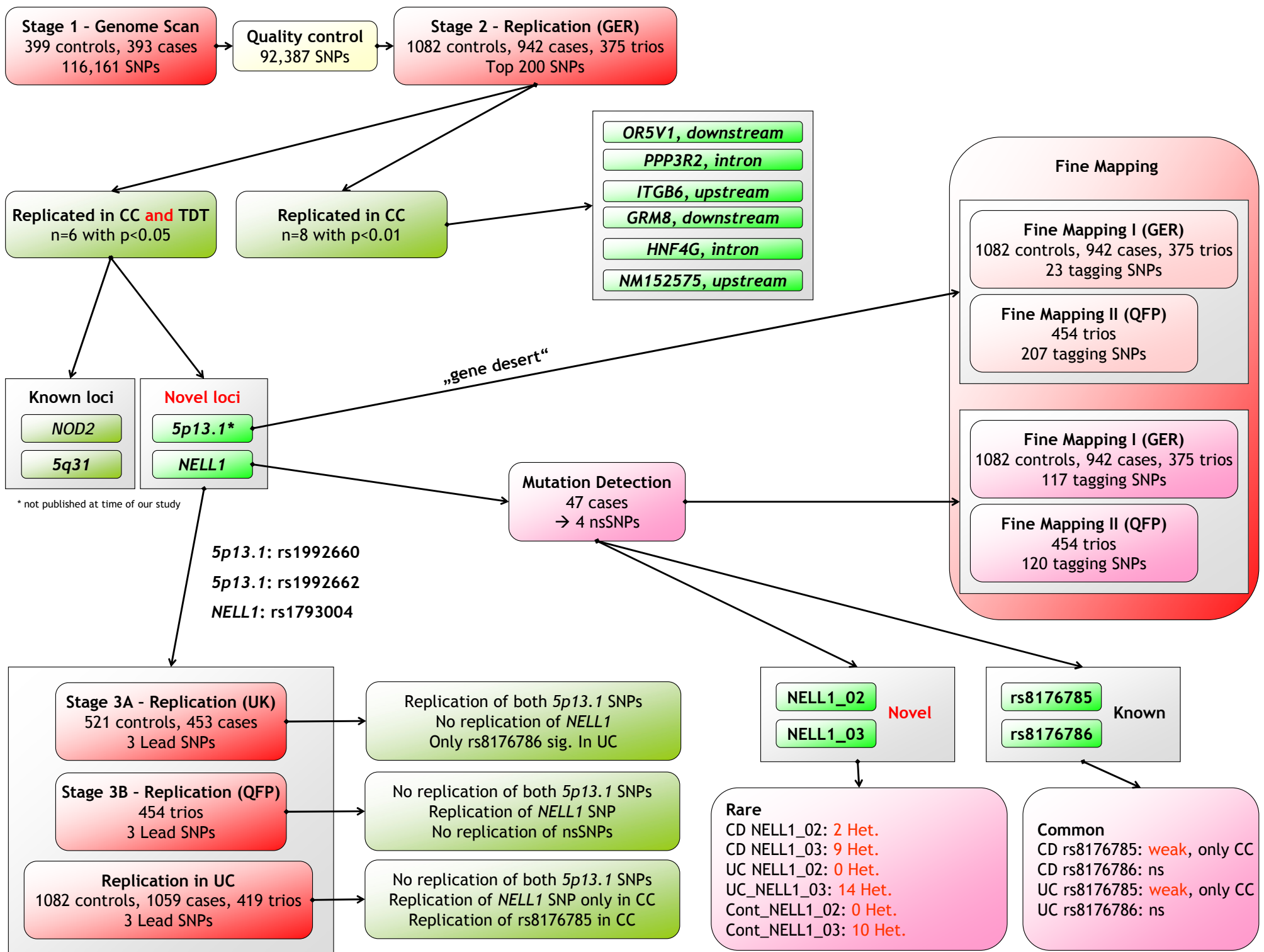

Supplement: Figure S1 — Workflow diagram summarizing the different stages of the experiment. (0.22 MB PDF) [file pone.0000691.s002.pdf]
