# Supplementary material for: Systematic Association Mapping Identifies NELL1 as a Novel IBD Disease Gene
Source: PLoS One. 2007 Aug 8;2(8):e691. doi: 10.1371/journal.pone.0000691 (PMC1933598; doi:10.1371/journal.pone.0000691)

Supplementary Figure 2: Power calculation for the present genome-wide association scan of 393 cases and 399 controls (α=0.05). Five different allele frequencies in controls (p_0) are shown: 50%, 30%, 20%, 10%, and 5%. The scan had e.g. 80% power to detect an Odds ratio of 1.6 assuming that 20% of the controls are exposed. Calculations were done using the software PS Power and Sample Size Calculations.

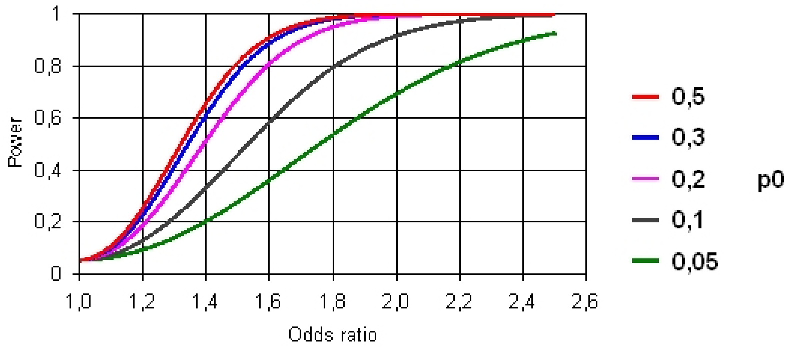

Supplement: Figure S2 — Power calculation for the present genome-wide association scan of 393 cases and 399 controls (a = 0.05). Five different allele frequencies in controls (p0) are shown: 50%, 30%, 20%, 10%, and 5%. The scan had e.g. 80% power to detect an Odds ratio of 1.6 assuming that 20% of the controls are exposed. Calculations were done using the software PS Power and Sample Size Calculations. (0.15 MB PDF) [file pone.0000691.s003.pdf]
