# Supplementary material for: Systematic Association Mapping Identifies NELL1 as a Novel IBD Disease Gene
Source: PLoS One. 2007 Aug 8;2(8):e691. doi: 10.1371/journal.pone.0000691 (PMC1933598; doi:10.1371/journal.pone.0000691)

Supplementary Figure 3: Quantile-quantile plots **(A)** before and **(B)** after exclusion of low-quality SNPs (callrate < 90%, p_{HWE} < 0.01, MAF > 0.99). The ranked, observed χ^2-values of the allelic test are plotted against the values expected for sampling from a χ^2 distribution with one degree of freedom (the distribution expected under the null hypothesis). The inflation of the χ^2 distribution is significantly reduced by removing low-quality SNPs and there is no visible indication of population structure. The outlier in the upper right corner of the diagram B represents genuine association of SNP rs2076756 in the *NOD2* gene with CD (see table S2, Lead #1).

A. n = 115,571 mapped SNPs

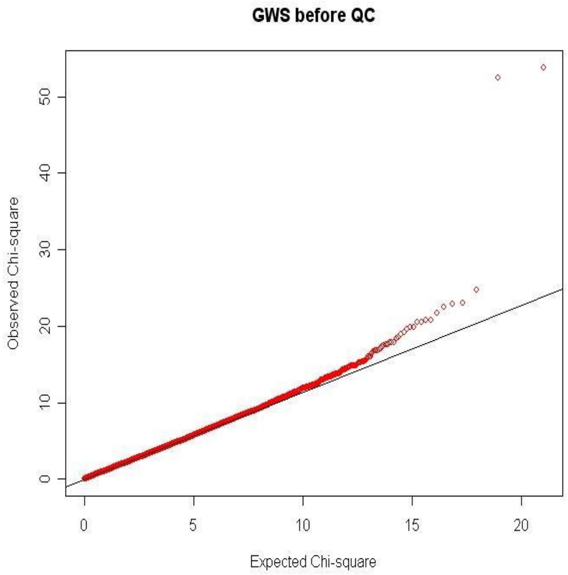

B. n = 92,387 SNPs

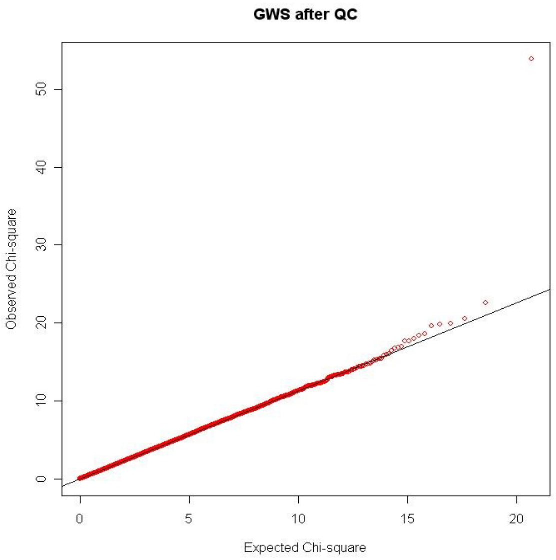

Supplement: Figure S3 — Quantile-quantile plots (A) before and (B) after exclusion of lowquality SNPs (callrate<90%, pHWE<0.01, MAF>0.99). The ranked, observed Chi2-values of the allelic test are plotted against the values expected for sampling from a Chi2 distribution with one degree of freedom (the distribution expected under the null hypothesis). The inflation of the Chi2 distribution is significantly reduced by removing low-quality SNPs and there is no visible indication of population structure. The outlier in the upper right corner of the diagram B represents genuine association of SNP rs2076756 in the NOD2 gene with CD (see Table S2, Lead #1). (0.22 MB PDF) [file pone.0000691.s004.pdf]
