# Supplementary material for: Systematic Association Mapping Identifies NELL1 as a Novel IBD Disease Gene
Source: PLoS One. 2007 Aug 8;2(8):e691. doi: 10.1371/journal.pone.0000691 (PMC1933598; doi:10.1371/journal.pone.0000691)
Supplement: Figure S4 — (A) Structure-based multiple sequence alignment of the N-terminal domains of NELL1 and NELL2 homologs and the N-terminal domain of human thrombospondin-1 (TSP-1). The DSSP secondary structure assignment of the TSPN structure (PDB code 1z78, chain A) is depicted at the top of the alignment. (B) Multiple sequence alignment of NELL1 and NELL2 homologs. Domain locations are represented as colored bars at the top of the alignment (green: VWC domain; pink: EGF-like domain). Alignment columns with more than 70% physicochemically similar amino acids are highlighted in blue boxes with white letters. Text labels point to the N-terminal signal peptide and the sequence variants, which are marked yellow in all homologs. Residue numbering in the alignment is based on complete protein sequences as derived from corresponding UniProt entries. (0.41 MB PDF) [file pone.0000691.s005.pdf]

Supplementary Figure 4: (A) Structure-based multiple sequence alignment of the N-terminal domains of NELL1 and NELL2 homologs and the N-terminal domain of human thrombospondin-1 (TSP-1). The DSSP secondary structure assignment of the TSPN structure (PDB code 1z78, chain A) is depicted at the top of the alignment. **(B)** Multiple sequence alignment of NELL1 and NELL2 homologs. Domain locations are represented as colored bars at the top of the alignment (green: VWC domain; pink: EGF-like domain). Alignment columns with more than 70% physicochemically similar amino acids are highlighted in blue boxes with white letters. Text labels point to the N-terminal signal peptide and the sequence variants, which are marked yellow in all homologs. Residue numbering in the alignment is based on complete protein sequences as derived from corresponding UniProt entries.

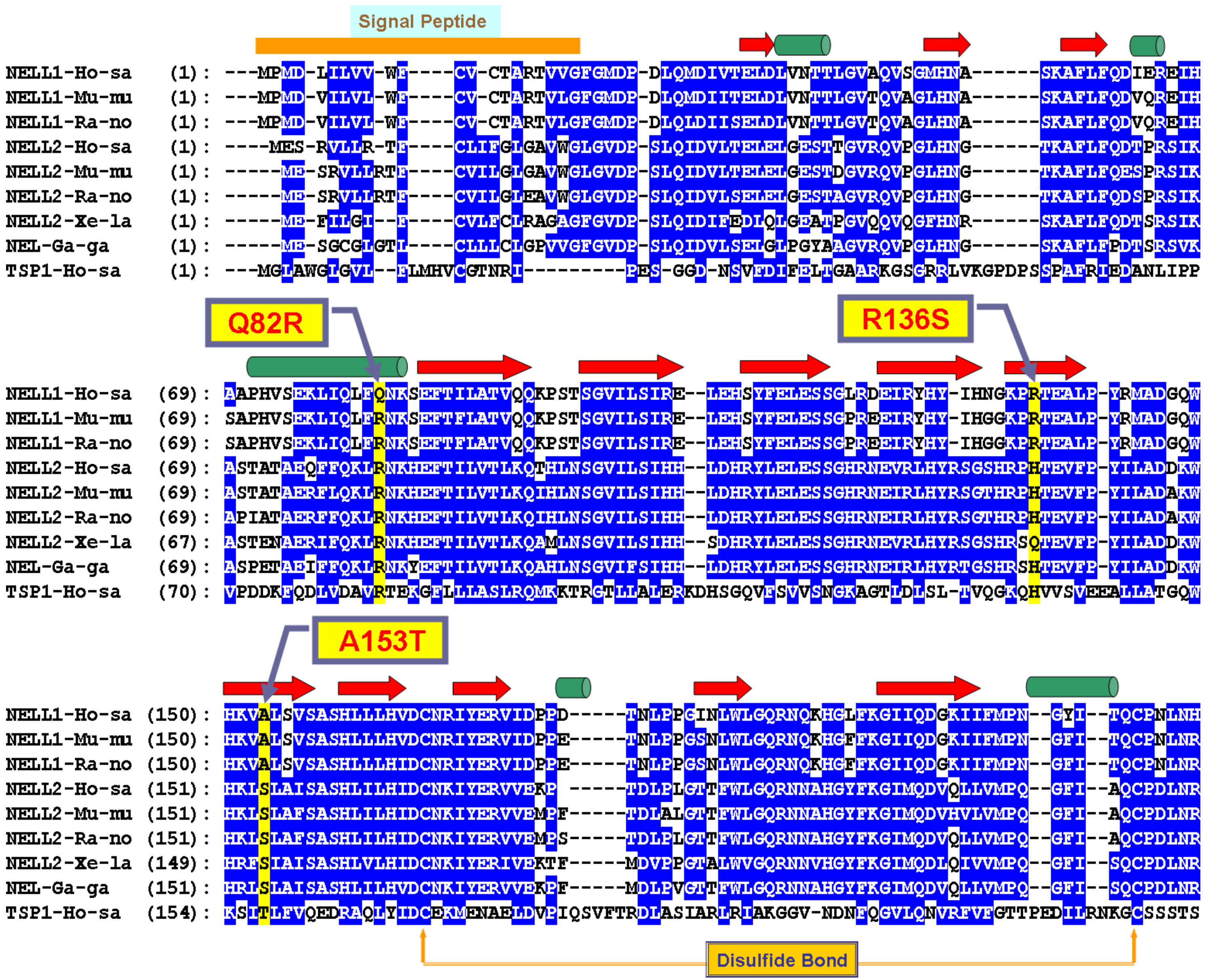
Signal Peptide
Q82R
R136S
A153T
Disulfide Bond

```
NELL1-Ho-sa  (226):TCPTCSDFLSLVQGIMDLQELLAKMTAKLNYAETRLSQLENCHCEKTCQVSGLLYRDQDSWVDGDHCRNCTCKSGAVECRRMSCP
NELL1-Mu-mu  (226):TCPTCSDFLSLVQGIMDLQELLAKMTAKLNYAETRLGQLENCHCEKTCQVSGLLYRDQDSWVDGDNCRNCTCKSGAVECRRMSCP
NELL1-Ra-no  (226):TCPTCSDFLSLVQGIMDLQELLAKMTAKLNYAETRLGQLENCHCEKTCQVSGLLYRDQDSWVDGDNCGNCTCKSGAVECRRMSCP
NELL2-Ho-sa  (227):TCPTCNDFHGLVQKIMELQDILAKTSAKLSRAEQRMNRLDQCYCERTCTMKGTTYREFESWIDG--CKNCTCLNGTIQCETLICP
NELL2-Mu-mu  (227):TCPTCNDFHGLVQKIMELQDILSKTSAKLSRAEQRMNRLDQCYCERTCTVKGTTYRESESWTDG--CKNCTCLNGTIQCETLVCP
NELL2-Ra-no  (227):TCPTCNDFHGLVQKIMELQDILSKTSAKLSRAEQRMNRLDQCYCERTCTMKGATYREFESWTDG--CKNCTCLNGTIQCETLVCP
NELL2-Xe-la  (225):TCPTCNDFHGLVQKIMELQDILAKTSAKLSRAEQRMNRLDQCYCERSCTVKGNIYRELESWMDG--CKKCTCTNGTAQCETLTCS
NEL-Ga-ga    (227):TCPTCNDFHGLVQKIMELQDILAKTSAKLSQAEQRMNKLDQCYCERTCTMKGMTYREFESWTDG--CKNCTCMNGTVQCEALICS

                                                   R354W
NELL1-Ho-sa  (311):PLNCSPDSLPVHIAGQCCKVCRPKCIYGGKVLAEGQRILTKSCR------ECRGGVLVKITEM-CPPLNCSEKDHILPENQCCRV
NELL1-Mu-mu  (311):PLNCSPDSLPVHISGQCCKVCRPKCIYGGKVLAEGQRILTKICR------ECRGGVLVKITEA-CPPLNCSEKDHILPENQCCRV
NELL1-Ra-no  (311):PLNCSPDSLPVHISGQCCKVCRPKCIYGGKVLAEGQRILTKICR------ECRGGVLVKITEA-CPPLNCSAKDHILPENQCCRV
NELL2-Ho-sa  (310):NPDCPLKSALAYVDGKCCKECKSICQFQGRTYFEGERNTVYSSSGVCVLYECKDQTMKLVESSGCPALDCPESHQITLSHSCCKV
NELL2-Mu-mu  (310):APDCPPKSAPAYVDGKCCKECKSTCQFQGRSYFEGERNTAYSSSGMCVLYECKDQTMKLVENIGCPPLDCPESHQIALSHSCCKV
NELL2-Ra-no  (310):APDCPAKSAPAYVDGKCCKECKSTCQFQGRSYFEGERSTVFSASGMCVLYECKDQTMKLVENAGCPALDCPESHQIALSHSCCKV
NELL2-Xe-la  (308):APNCLSGFSPAYVPGKCCKECQTVCVFQGQMYFEEEREAVYSSSGQCVLFQCKDNTMRRIESPECLPLNCPQSQHITLRNSCCKV
NEL-Ga-ga    (310):LSDCPPNSALSYVDGKCCKECQSVCIFEGRTYFEGQRETVYSSSGDCVLFECKDHKMQRIPKDSCATLNCPESQQIPLSHSCCKI

NELL1-Ho-sa  (389):CRGHNFCAEGPKCGENSECKNWNTKATCECKSGYISVQGDSAYCEDIDECAAKMHYCHANTVCVNLPGLYRCDCVPGYIRVDDFS
NELL1-Mu-mu  (389):CRGHNFCAEAPKCGENSECKNWNTKATCECKNGYISVQGNSAYCEDIDECAAKMHYCHANTVCVNLPGLYRCDCIPGYIRVDDFS
NELL1-Ra-no  (389):CPGHNFCAEAPKCGENSECKNWNTKATCECKNGYISVQGNSAYCEDIDECAAKMHYCHANTVCVNLPGLYRCDCVPGYIRVDDFS
NELL2-Ho-sa  (395):CKGYDFCSERHNCMENSICRNLNDRAVCSCRDGFRALREDNAYCEDIDECAEGRHYCRENTMCVNTPGSFMCICKTGYIRIDDYS
NELL2-Mu-mu  (395):CKGYDFCSEKHTCMENSVCRNLNDRVVCSCRDGFRALREDNAYCEDIDECAEGRHYCRENTMCVNTPGSFMCVCKTGYIRIDDYS
NELL2-Ra-no  (395):CKGYDFCSEKHTCMENSVCRNLNDRAVCSCRDGFRALREDNAYCEDIDECAEGRHYCRENTMCVNTPGSFLCICQTGYIRIDDYS
NELL2-Xe-la  (393):CKGHDFCSEGHNCMGYSICKNLDDKAVCICRDGFRALREDNAYCEDIDECTEGRHYCRENTVCVNTPGSFMCVCQTGYLKIDDYS
NEL-Ga-ga    (395):CKGHDFCTEGHNCMEHSVCRNLDDRAVCSCRDGFRALREDNAYCEDVDECAEGQHYCRENTMCVNTPGSFMCICKTGYIRIDDYS

NELL1-Ho-sa  (474):CTEHDECGSGQHNCDENAICTNTVQGHSCTCKPGYVGNGTICRAFCEEGCRYGGTCVAPNKCVCPSGFTGSHCEKDIDECSEGII
NELL1-Mu-mu  (474):CTEHDDCGSGQHNCDKNAICTNTVQGHSCTCQPGYVGNGTVCKAFCEEGCRYGGTCVAPNKCVCPSGFTGSHCEKDIDECAEGFV
NELL1-Ra-no  (474):CTEHDDCGSGQHNCDKNAICTNTVQGHSCTCQPGYVGNGTICKAFCEEGCRYGGTCVAPNKCVCPSGFTGSHCEKDIDECAEGFV
NELL2-Ho-sa  (480):CTEHDECITNQHNCDENALCFNTVGGHNCVCKPGYTGNGTTCKAFCKDGCRNGGACIAANVCACPQGFTGPSCETDIDECSDGFV
NELL2-Mu-mu  (480):CTEHDECLTTQHNCDENALCFNTVGGHNCVCKPGYTGNGTTCKAFCKDGCRNGGACIAANVCACPQGFTGPSCETDIDECSEGFV
NELL2-Ra-no  (480):CTEHDECLTNQHNCDENALCFNTVGGHNCVCKPGYTGNGTTCKAFCKDGCKNGGACIAANVCACPQGFTGPSCETDIDECSEGFV
NELL2-Xe-la  (478):CTEHNECATNQHSCDENAMCFNTVGGHNCVCQPGYTGNGTDCRAFCKDGCRNGGTCIAPNICACPQGFTGPSCESDIDECTEGFV
NEL-Ga-ga    (480):CTEHDECVTNQHNCDENALCFNTVGGHNCVCKLGYTGNGTVCKAFCKDGCRNGGACIASNVCACPQGFTGPSCETDIDECSDGFV

NELL1-Ho-sa  (559):ECHNHSRCVNLPGWYHCECRSGFHDDGTYSLSGESCIDIDECALRTHTCWNDSACINLAGGFDCLCPSGPSCSGDCPHEGGLKHN
NELL1-Mu-mu  (559):ECHNHSRCVNLPGWYHCECRSGFHDDGTYSLSGESCIDIDECALRTHTCWNDSACINLAGGFDCLCPSGPSCSGDCPHEGGLKHN
NELL1-Ra-no  (559):ECHNYSRCVNLPGWYHCECRSGFHDDGTYSLSGESCIDIDECALRTHTCWNDSACINLAGGFDCLCPSGPSCSGDCPHEGGLKHN
NELL2-Ho-sa  (565):QCDSRANCINLPGWYHCECRDGYHDNGMFSPSGESCEDIDECGTGRHSCANDTICFNLDGGYDCRCPHGKNCTGDCIHDGKVKHN
NELL2-Mu-mu  (565):QCDSRANCINLPGWYHCECRDGYHDNGMFAPGGESCEDIDECGTGRHSCTNDTICFNLDGGYDCRCPHGKNCTGDCVHEGKVKHT
NELL2-Ra-no  (565):QCDSRANCINLPGWYHCECRDGYHDNGMFAPGGESCEDIDECGTGRHSCANDTICFNLDGGYDCRCPHGKNCTGDCVHDGKVKHN
NELL2-Xe-la  (563):QCDSRANCINLPGWYHCECRDGYHDNGMFSLGGESCEDIDECATGRHSCSNDTVCFNLDGGFDCRCPHGKNCSGDCTHEGKIKHN
NEL-Ga-ga    (565):QCDSRANCINLPGWYHCECRDGYHDNGMFSPSGESCEDIDECATGRHSCANDTVCFNLDGGYDCRCPHGKNCTGDCIHEDKIKHN

NELL1-Ho-sa  (644):GQVWTLKEDRCSVCSCKDGKIFCRRTACDCQNPSADLFCCPECDTRVTSQCLDQNGHKLYRSGDNWTHSCQQCRCLEGEVDCWPL
NELL1-Mu-mu  (644):GQVWILREDRCSVCSCKDGKIFCRRTACDCQNPNVDLFCCPECDTRVTSQCLDQSGQKLYRSGDNWTHSCQQCRCLEGEADCWPL
NELL1-Ra-no  (644):GQVWILREDRCSVCSCKDGKIFCRRTACDCQNPNVDLFCCPECDTRVTSQCLDQSGQKLYRSGDNWTHSCQQCRCLEGEADCWPL
NELL2-Ho-sa  (650):GQIWVLENDRCSVCSCQNGFVMCRRMVCDCENPTVDLFCCPECDPRLSSQCLHQNGETLYNSGDTWVQNCQQCRCLQGEVDCWPL
NELL2-Mu-mu  (650):GQIWVLENDRCSVCSWQTGFVMCRRMVCDCENPTDDLSCCPECDPRLSSQCLHQNGETVYNSGDTWVQDCRQCRCLQGEVDCWPL
NELL2-Ra-no  (650):GQIWVLENDRCSVCSCQTGFVMCQRMVCDCENPTVDLSCCPECDPRLSSQCLHQNGETVYNSGDTWAQDCRQCRCLQEEVDCWPL
NELL2-Xe-la  (648):GQIWVLENDRCSVCSCQVGLVMCRRMVCDCENPTVDLFCCPECDPRLSSQCLHQSGELTYKSGDTWVQNCQQCRCLQGEVDCWPL
NEL-Ga-ga    (650):GQIWVLENDRCSVCSCQSGYVMCRRMVCDCENPTVDLFCCPECDPRLSSQCLHQSGELSYNSGDSWIQNCQQCRCLQGEVDCWPL

NELL1-Ho-sa  (729):TCPNLSCEYTAILEGECCPRCVSDPCLADNITYDIRKTCLDSYGVSRLSGSVWTMAGSPCTTCKCKNGRVCCSVDFECLQNN
NELL1-Mu-mu  (729):ACPSLSCEYTAIFEGECCPRCVSDPCLADNIAYDIRKTCLDSSGISRLSGAVWTMAGSPCTTCQCKNGRVCCSVDLVCLENN
NELL1-Ra-no  (729):ACPSLGCEYTAMFEGECCPRCVSDPCLAGNIAYDIRKTCLDSFGVSRLSGAVWTMAGSPCTTCKCKNGRVCCSVDLECIENN
NELL2-Ho-sa  (735):PCPDVECEFSILPENECCPRCVTDPCQADTIRNDITKTCLDEMNVVRFTGSSWIKHGTECTLCQCKNGHICCSVDPQCLQEL
NELL2-Mu-mu  (735):ACPEVECEFSVLPENECCPRCVTDPCQADTIRNDITKTCLDEMNVVRFTGSSWIKHGTECTLCQCKNGHLCCSVDPQCLQEL
NELL2-Ra-no  (735):ACPEVECEFSVLPENECCPRCVTDPCQADTIRNDITKTCLDEMNVVRFTGSSWIKHGTECTLCQCKNGHVCCSVDPQCLQEL
NELL2-Xe-la  (733):PCPAIDCEFSVVPESECCPRCVSDPCQADIIRNDITKTCVDETNVVRFTGSSWIKHGTECTLCQCKNGHMCCSVDPQCLQEL
NEL-Ga-ga    (735):PCPEVDCEFSVLPENECCPRCVTDPCQADTIRNDITKTCLDETNVVRFTGSSWIKHGTECTLCQCKNGHVCCSVDPQCLQEL
```

EGF

VWC
