# Supplementary material for: Systematic Association Mapping Identifies NELL1 as a Novel IBD Disease Gene
Source: PLoS One. 2007 Aug 8;2(8):e691. doi: 10.1371/journal.pone.0000691 (PMC1933598; doi:10.1371/journal.pone.0000691)
Supplement: Figure S5 — Overview of the results for the 5p13.1 locus. (A) Plot of the negative common logarithm of the p-values of the different tiers across the 650 kb region. The red line shows the significance threshold of p = 0.05. Results of the two lead SNPs rs1992662 and rs1992660 are highlighted in pink color. The broad replicated peak between 40.29 Mb and 40.66 Mb localizes to a gene desert upstream of PTGER4. (B) Recombination rate in cM/Mb shows that the peak region is delineated by two sites of increased recombination. (C) Linkage-disequilibrium (LD) plot from HapMap, using the metrics D′ and (D) r2. Genotypes of trios with northern and western European ancestry for 633 SNPs (CR> = 90%, MAF> = 1%, pHWE> = 0.01, Mendel errors> = 3) were retrieved from HapMap. Positions are from NCBI build 35. (1.36 MB PDF) [file pone.0000691.s006.pdf]

Supplementary Figure 5: Overview of the results for the *5p13.1* locus. **(A)** Plot of the negative common logarithm of the p-values of the different tiers across the 650 kb region. The red line shows the significance threshold of $p = 0.05$. Results of the two lead SNPs rs1992662 and rs1992660 are highlighted in pink color. The broad replicated peak between 40.29 Mb and 40.66 Mb localizes to a gene desert upstream of *PTGER4*. **(B)** Recombination rate in cM/Mb shows that the peak region is delineated by two sites of increased recombination. **(C)** Linkage-disequilibrium (LD) plot from HapMap, using the metrics D' and **(D)** r^2. Genotypes of trios with northern and western European ancestry for 633 SNPs (CR ≥ 90%, MAF ≥ 1%, $p_{HWE} \geq 0.01$, Mendel errors ≤ 3) were retrieved from HapMap. Positions are from NCBI build 35.

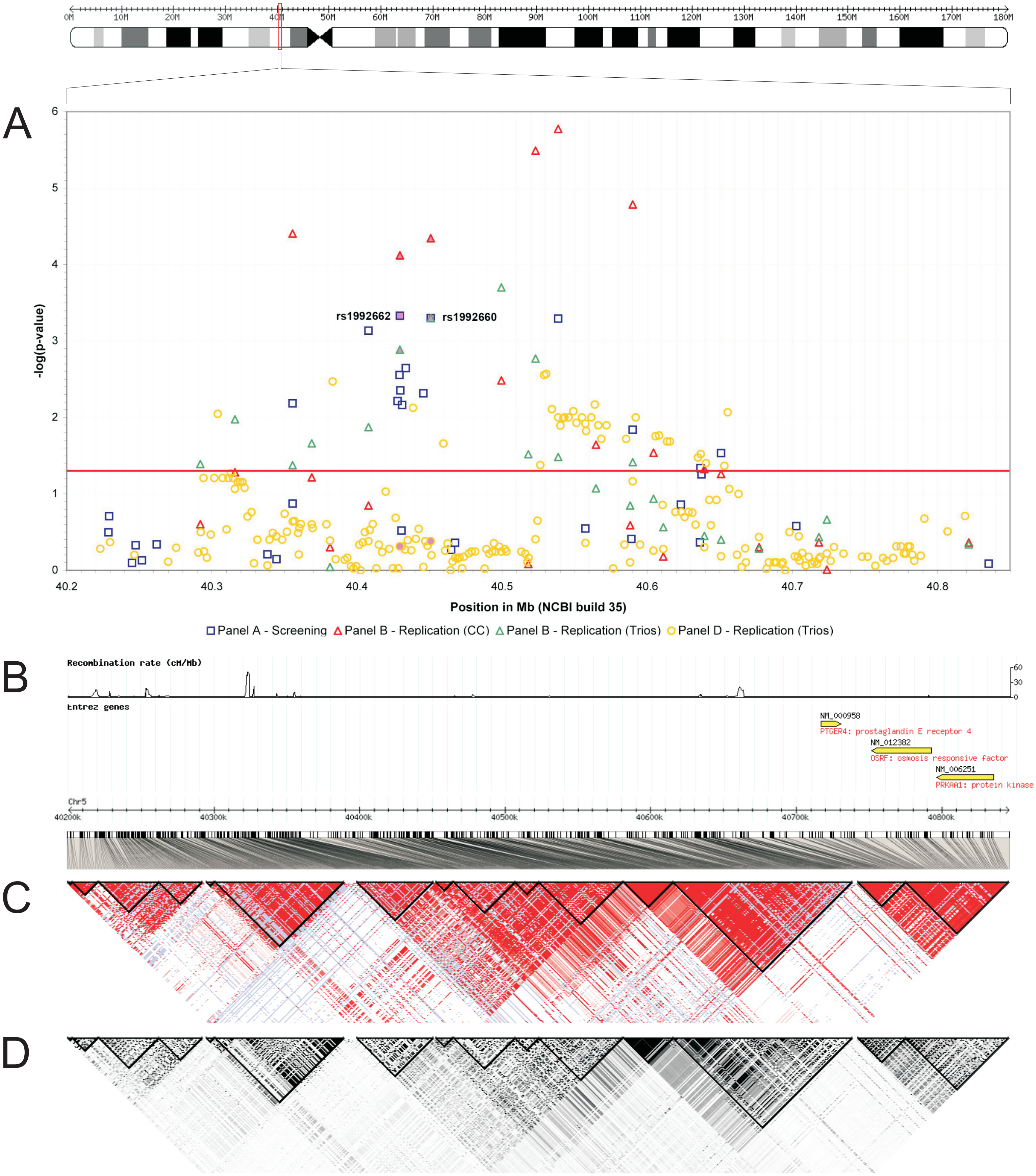

A
B
C
D
-log(p-value)
Position in Mb (NCBI build 35)
rs1992662
rs1992660
Panel A - Screening
Panel B - Replication (CC)
Panel B - Replication (Trios)
Panel D - Replication (Trios)
Recombination rate (cM/Mb)
Entrez genes
NM_000958
PTGER4: prostaglandin E receptor 4
NM_012382
OSRF: osmosis responsive factor
NM_006251
PRKAA1: protein kinase
Chr5
