# Supplementary material for: Systematic Association Mapping Identifies NELL1 as a Novel IBD Disease Gene
Source: PLoS One. 2007 Aug 8;2(8):e691. doi: 10.1371/journal.pone.0000691 (PMC1933598; doi:10.1371/journal.pone.0000691)
Supplement: Figure S6 — Secondary structure prediction for human NELL1 (UniProt accession number Q92832). The PSIPRED web server produced the depicted prediction. (0.13 MB PDF) [file pone.0000691.s007.pdf]

Supplementary Figure 6: Secondary structure prediction for human NELL1 (UniProt accession number Q92832). The PSIPRED web server produced the depicted prediction.

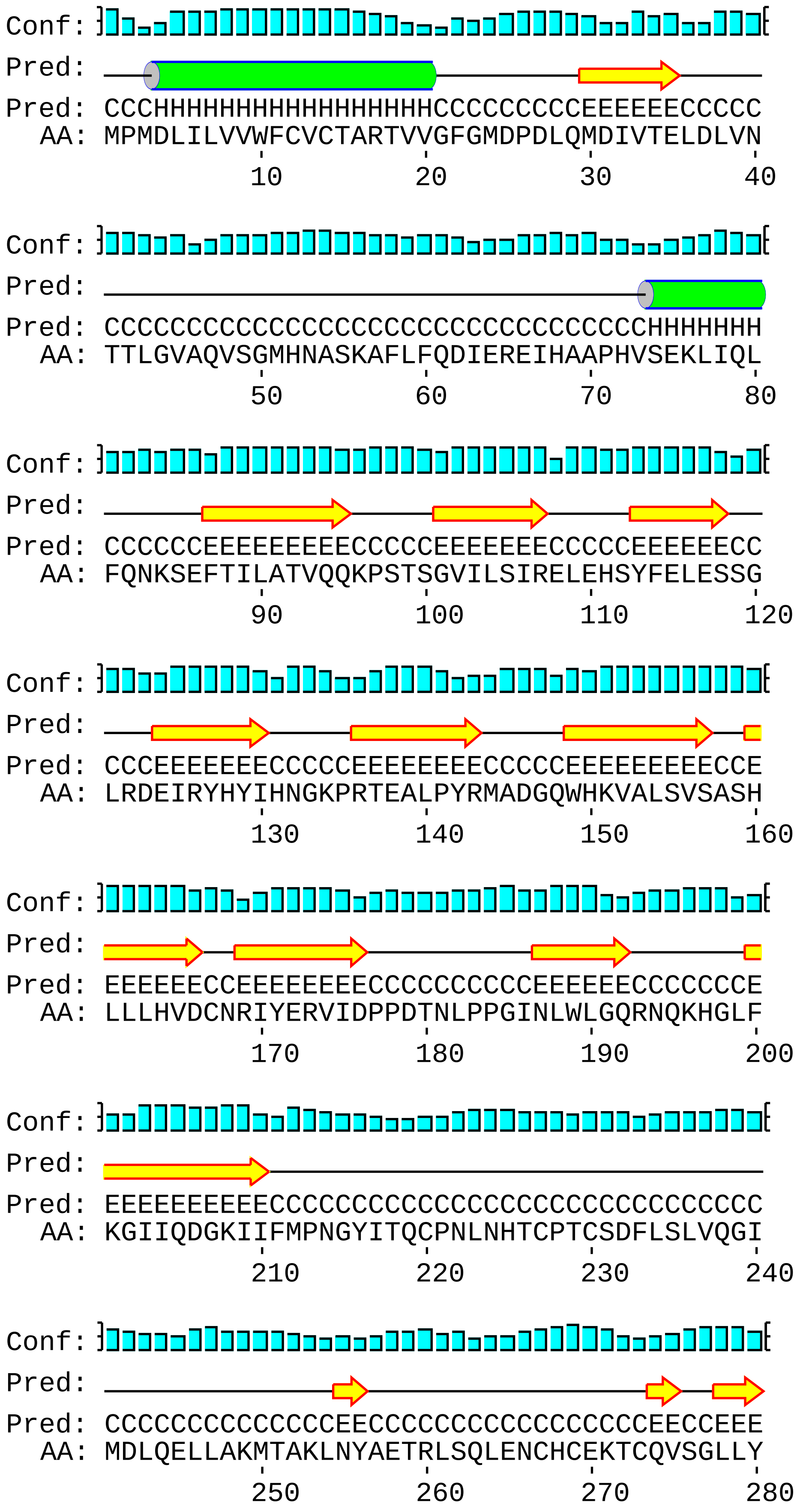
Conf:
Pred:
Pred: CCCHHHHHHHHHHHHHHHHHCCCCCCCCCEEEEEECCCCC
AA: MPMDLILVVWFCVCTARTVVGFGMDPDLQMDIVTELDLVN
10 20 30 40
Conf:
Pred:
Pred: CCCCCCCCCCCCCCCCCCCCCCCCCCCCCCCCCHHHHHHH
AA: TTLGVAQVSGMHNASKAFLFQDIEREIHAAPHVSEKLIQL
50 60 70 80
Conf:
Pred:
Pred: CCCCCCEEEEEEEEECCCCCEEEEEEECCCCCEEEEEECC
AA: FQNKSEFTILATVQQKPSTSGVILSIRELEHSYFELESSG
90 100 110 120
Conf:
Pred:
Pred: CCCEEEEEEECCCCCEEEEEEEECCCCCEEEEEEEEECCE
AA: LRDEIRYHYIHNGKPRTEALPYRMADGQWHKVALSVSASH
130 140 150 160
Conf:
Pred:
Pred: EEEEEECCEEEEEEEECCCCCCCCCCEEEEEECCCCCCCE
AA: LLLHVDCNRIYERVIDPPDTNLPPGINLWLGQRNQKHGLF
170 180 190 200
Conf:
Pred:
Pred: EEEEEEEEEECCCCCCCCCCCCCCCCCCCCCCCCCCCCCC
AA: KGIIQDGKIIFMPNGYITQCPNLNHTCPTCSDFLSLVQGI
210 220 230 240
Conf:
Pred:
Pred: CCCCCCCCCCCCCCEECCCCCCCCCCCCCCCCCEECCEEE
AA: MDLQELLAKMTAKLNYAETRLSQLENCHCEKTCQVSGLLY
250 260 270 280

```
Conf:
Pred:
Pred: CCCCCEECCCCCCCCCCCCCCEEECCCCCCCCCCCCCCCC
  AA: RDQDSWVDGDHCRNCTCKSGAVECRRMSCPPLNCSPDSLP
                290       300       310       320

Conf:
Pred:
Pred: CCCCCCEEEEEEEEECCCCEECCCCCCCCCCCCCCCCCCE
  AA: VHIAGQCCKVCRPKCIYGGKVLAEGQRILTKSCRECRGGV
                330       340       350       360

Conf:
Pred:
Pred: ECCCCCCCCCCCCCCCCCEEECCCCCCCCCCCCCCCCCCC
  AA: LVKITEMCPPLNCSEKDHILPENQCCRVCRGHNFCAEGPK
                370       380       390       400

Conf:
Pred:
Pred: CCCCCEEECCCCCCEEECCCCCCCCCCCCCCCCCCCCCCC
  AA: CGENSECKNWNTKATCECKSGYISVQGDSAYCEDIDECAA
                410       420       430       440

Conf:
Pred:
Pred: CCCCCCCCCEEEECCCCEEEEECCCCCCCCCCCCCCCCCC
  AA: KMHYCHANTVCVNLPGLYRCDCVPGYIRVDDFSCTEHDEC
                450       460       470       480

Conf:
Pred:
Pred: CCCCCCCCCCCEEEECCCCEECCCCCCEECCCCCCCCCCC
  AA: GSGQHNCDENAICTNTVQGHSCTCKPGYVGNGTICRAFCE
                490       500       510       520

Conf:
Pred:
Pred: CCCCCCCEECCCCEEECCCCCCCCCCEEEEEEECCCCCCC
  AA: EGCRYGGTCVAPNKCVCPSGFTGSHCEKDIDECSEGIIEC
                530       540       550       560
```

```
Conf:
Pred:
Pred: CCCCEEECCCCCEEECCCCCCCCCCCCCCCCCCCCCCCCC
  AA: HNHSRCVNLPGWYHCECRSGFHDDGTYSLSGESCIDIDEC
                      570       580       590       600

Conf:
Pred:
Pred: CCCCCCCCCCCCEEECCCCCCCCCCCCCCCCCCCCCCCCC
  AA: ALRTHTCWNDSACINLAGGFDCLCPSGPSCSGDCPHEGGL
                      610       620       630       640

Conf:
Pred:
Pred: CCCCCCEECCCCCCCEECCCCCCCCCCCCCCCCCCCCCCC
  AA: KHNGQVWTLKEDRCSVCSCKDGKIFCRRTACDCQNPSADL
                      650       660       670       680

Conf:
Pred:
Pred: CCCCEEECCCCCCCCCCCCCEEECCCCCCCCCCCCCEECC
  AA: FCCPECDTRVTSQCLDQNGHKLYRSGDNWTHSCQQCRCLE
                      690       700       710       720

Conf:
Pred:
Pred: CCCCCCCCCCCCCCCCCCCCCCCCCCCCCCCCCCCCCCEE
  AA: GEVDCWPLTCPNLSCEYTAILEGECCPRCVSDPCLADNIT
                      730       740       750       760

Conf:
Pred:
Pred: ECCCCCCCCCCCCCCCCCCCCCCCCCCCCCCCCCCCCEEE
  AA: YDIRKTCLDSYGVSRLSGSVWTMAGSPCTTCKCKNGRVCC
                      770       780       790       800

Conf:
Pred:
Pred: CCCCEECCCC
  AA: SVDFECLQNN
                      810
```

Legend:

= helix Conf: = confidence of prediction

- +

= strand Pred: predicted secondary structure

= coil AA: target sequence
