# Supplementary material for: Systematic Association Mapping Identifies NELL1 as a Novel IBD Disease Gene
Source: PLoS One. 2007 Aug 8;2(8):e691. doi: 10.1371/journal.pone.0000691 (PMC1933598; doi:10.1371/journal.pone.0000691)
Supplement: Figure S7 — The diagrams on the first page show the results of the whole genome association scan for Crohn disease and subsequent pages show the enlarged diagrams for each chromosome. The negative common logarithm of the p-values for the allelic test are shown. Only markers that passed the quality criteria listed in Table S1 were used for plotting (n = 92,387). “Outlier” SNP rs2076756 in the CARD15 gene (pCCA<10–12, 50.53 Mb) was omitted for illustration purposes. Marker positions are from NCBI build 34. (11.06 MB PDF) [file pone.0000691.s008.pdf]

Supplementary Plots: The diagrams on the first page show the results of the whole genome association scan for Crohn disease and subsequent pages show the enlarged diagrams for each chromosome. The negative common logarithm of the p-values for the allelic test are shown. Only markers that passed the quality criteria listed in table S1 were used for plotting (n = 92,387). "Outlier" SNP rs2076756 in the *CARD15* gene ($p_{CCA} < 10^{-12}$, 50.53 Mb) was omitted for illustration purposes. Marker positions are from NCBI build 34.

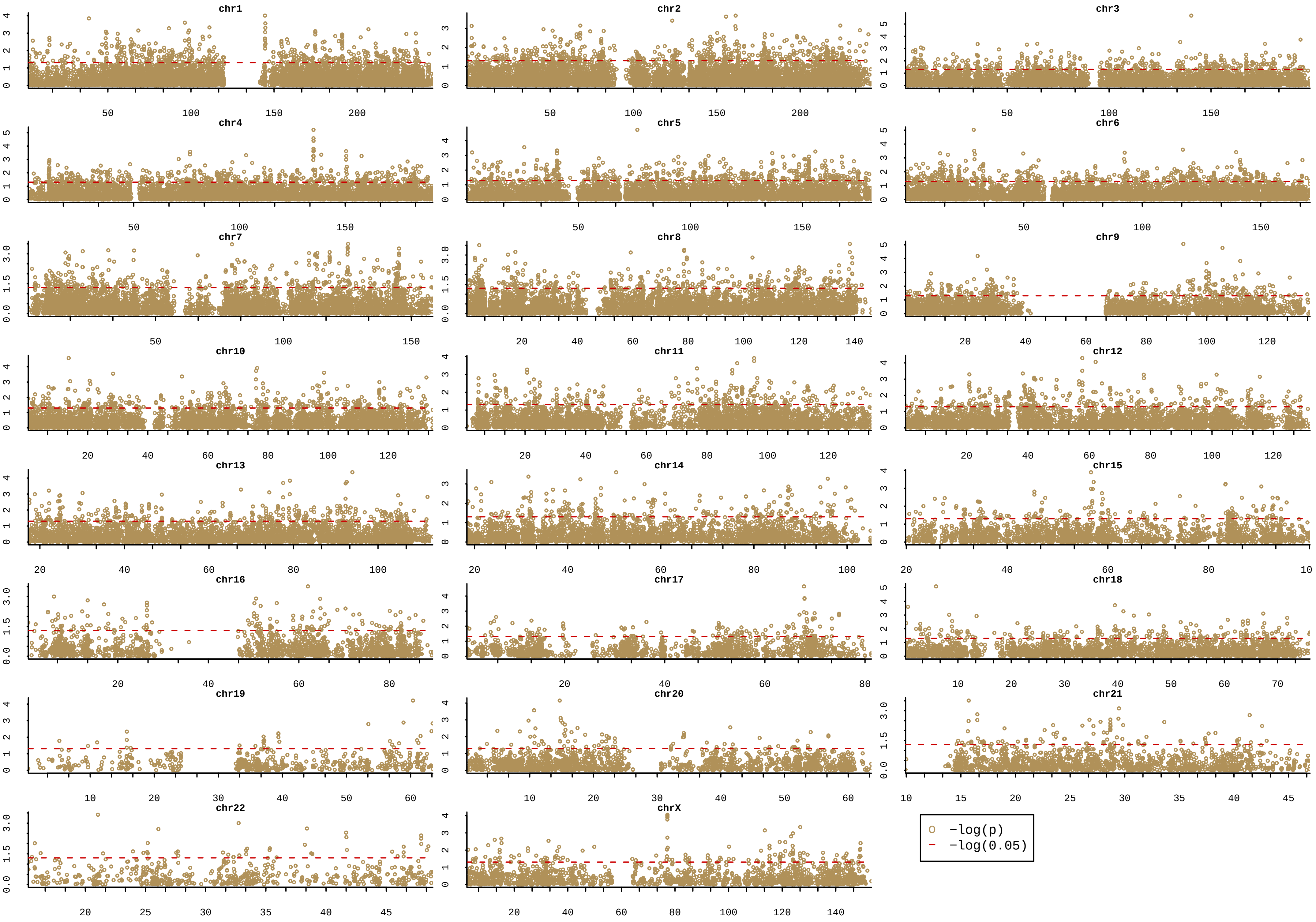
chr1
chr2
chr3
chr4
chr5
chr6
chr7
chr8
chr9
chr10
chr11
chr12
chr13
chr14
chr15
chr16
chr17
chr18
chr19
chr20
chr21
chr22
chrX
-log(p)
-log(0.05)

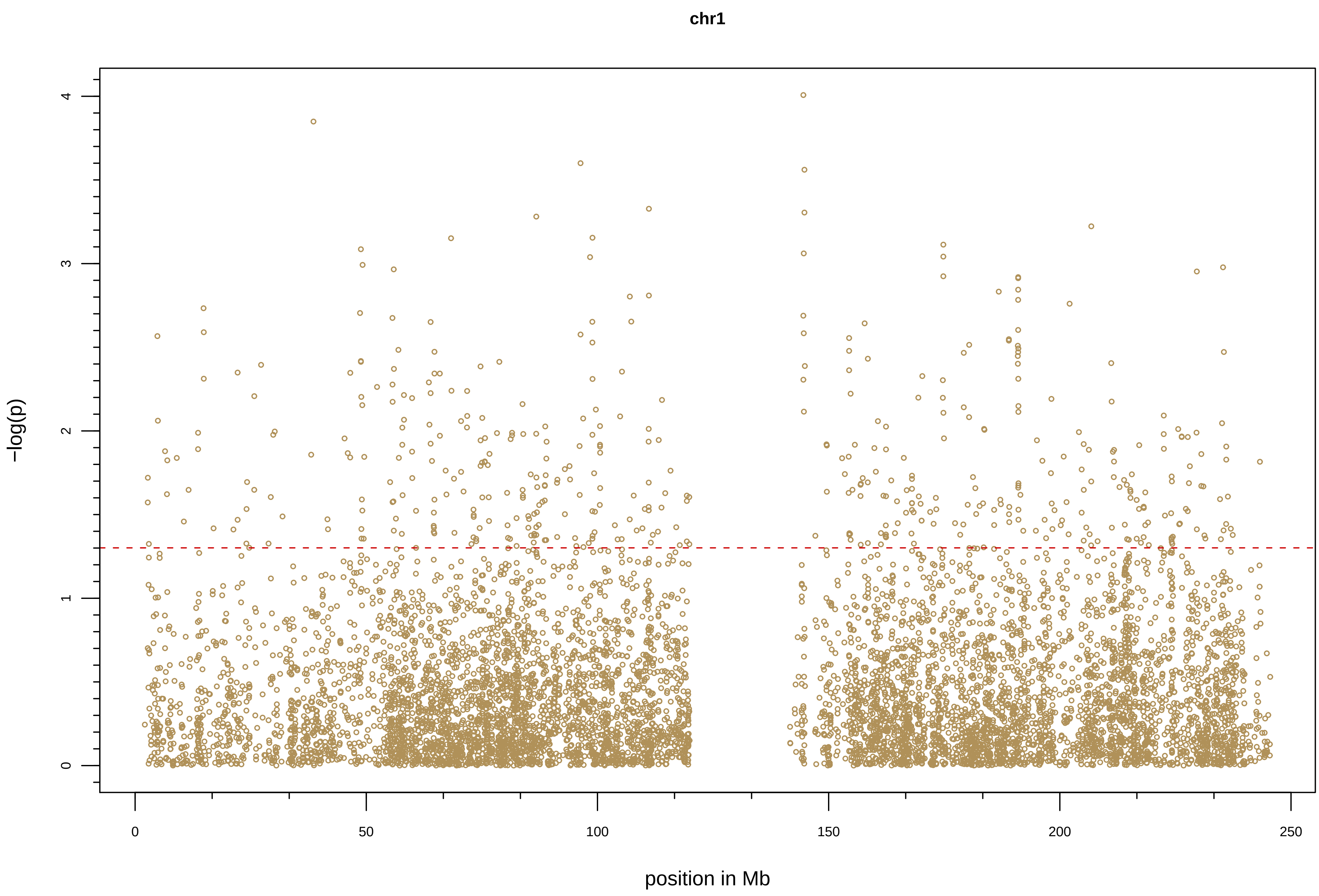
chr1
−log(p)
0
1
2
3
4
position in Mb
0
50
100
150
200
250

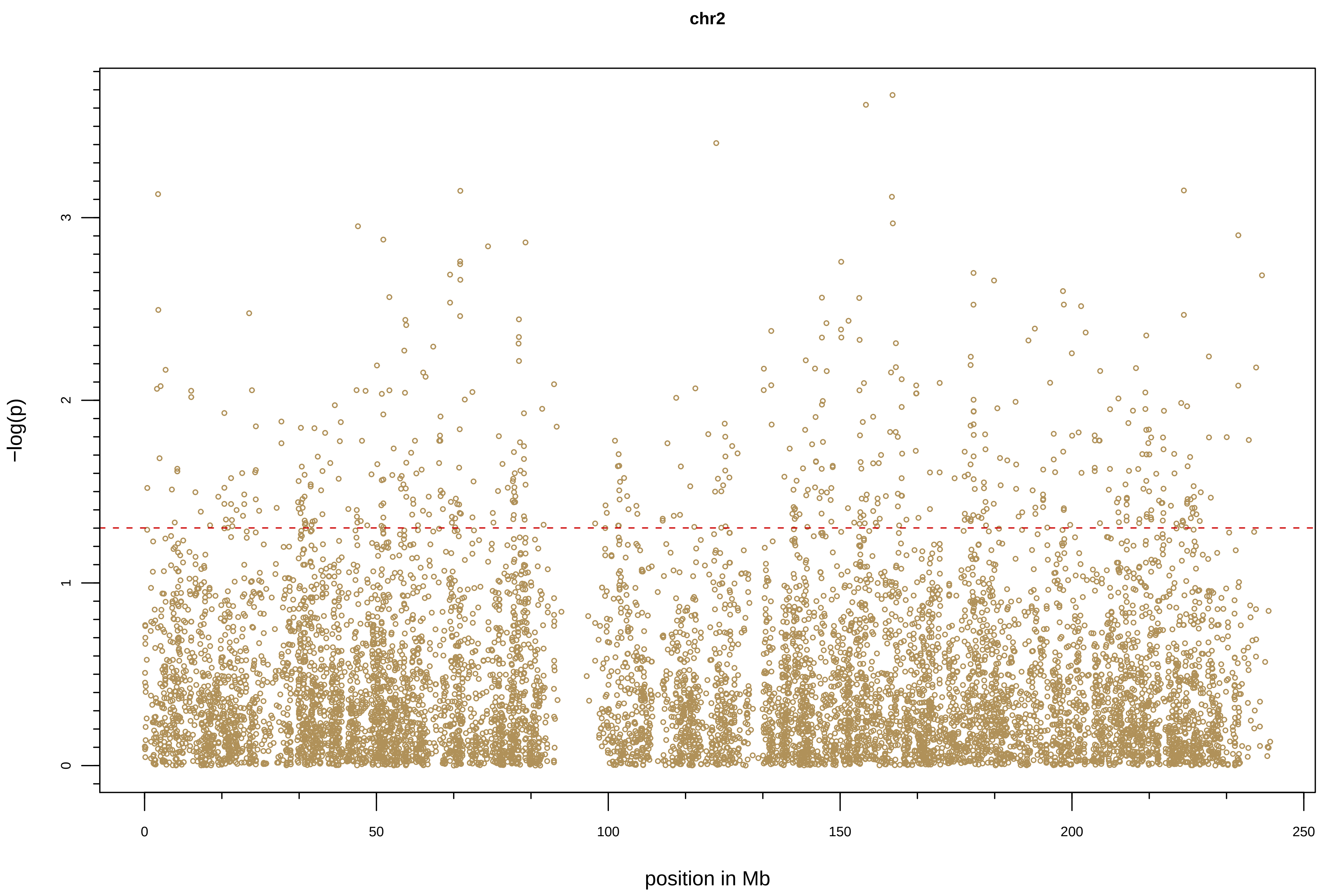
chr2
-log(p)
position in Mb
0
1
2
3
0
50
100
150
200
250

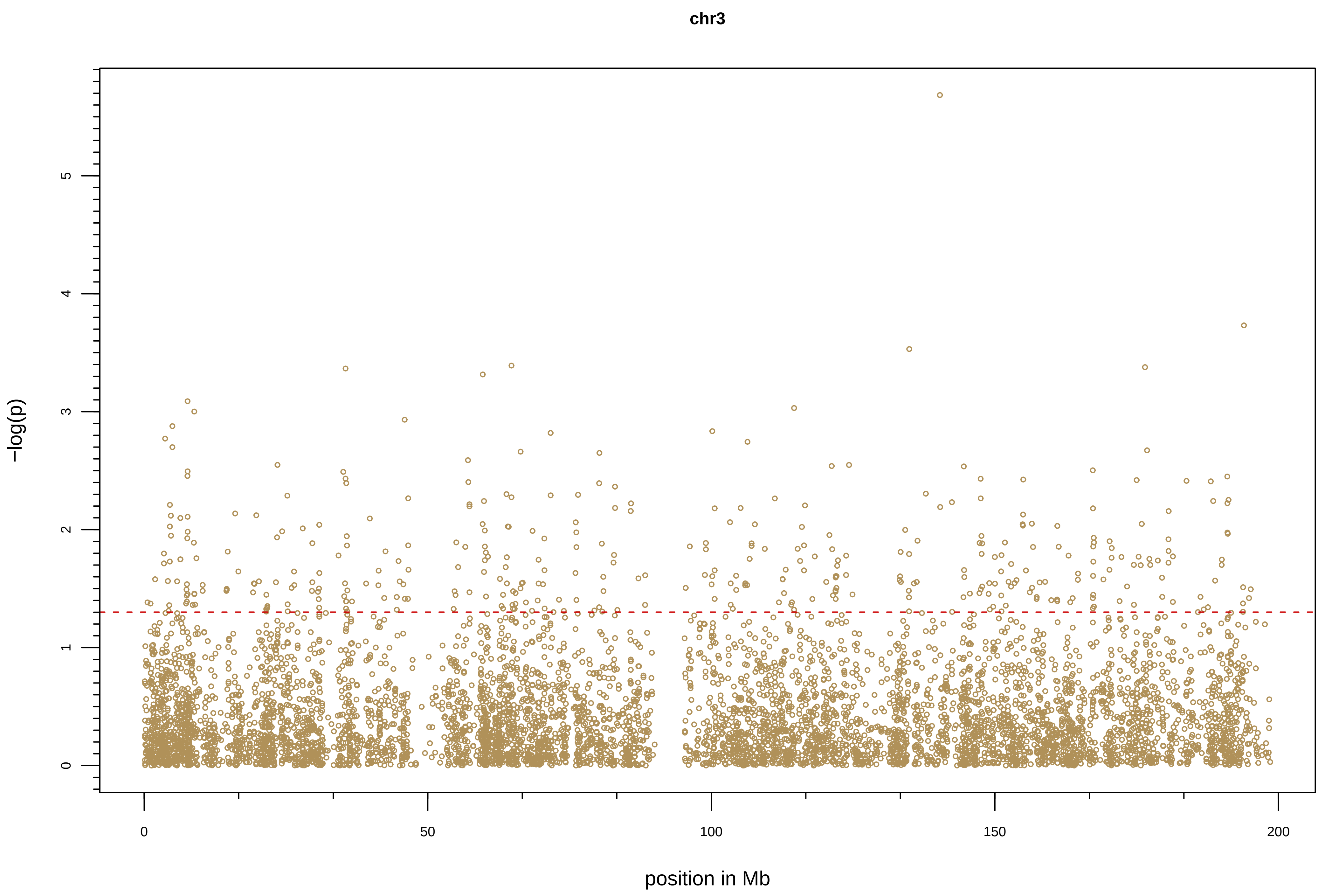

chr3
−log(p)
0
1
2
3
4
5
0
50
100
150
200
position in Mb

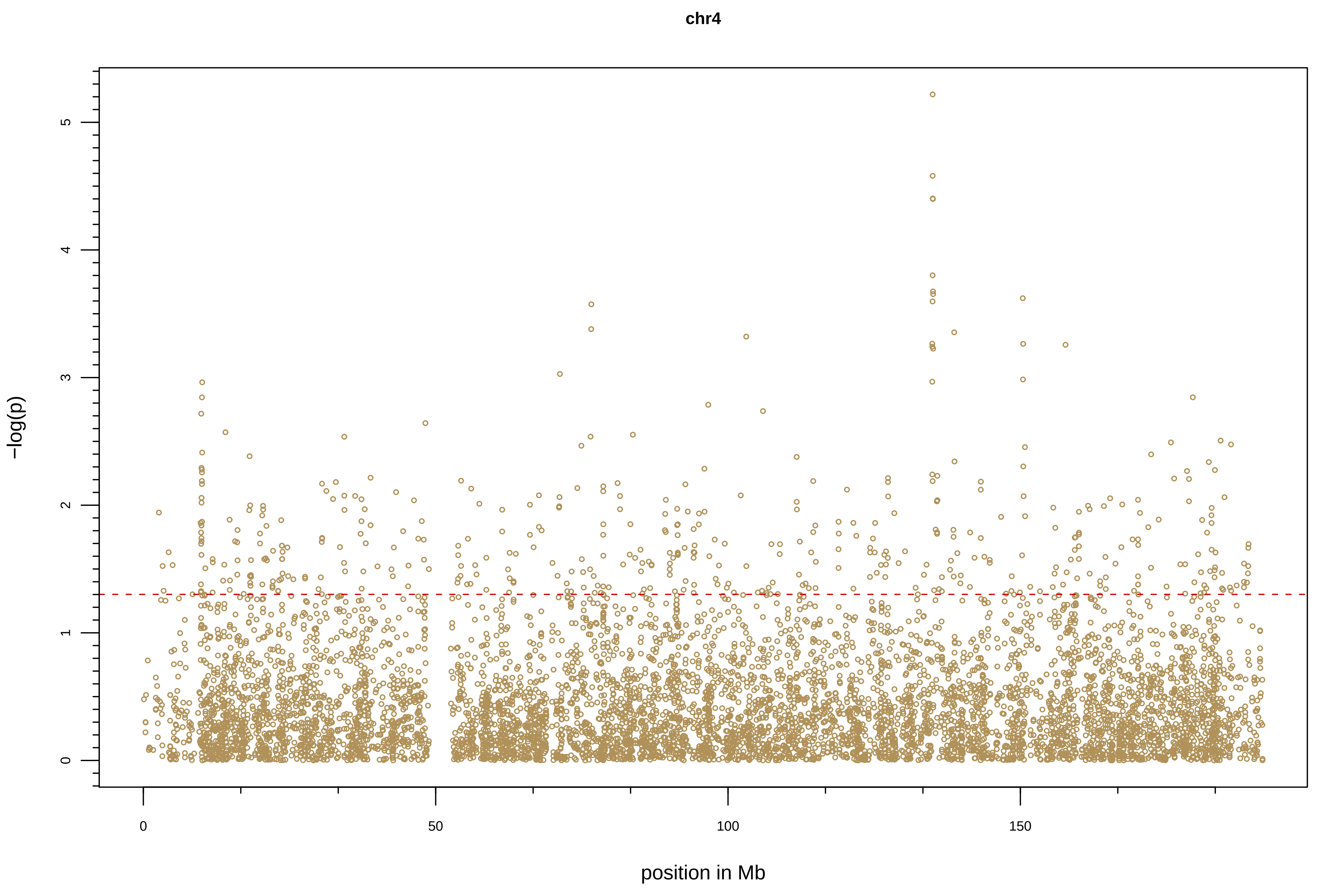
chr4
−log(p)
0
1
2
3
4
5
0
50
100
150
position in Mb

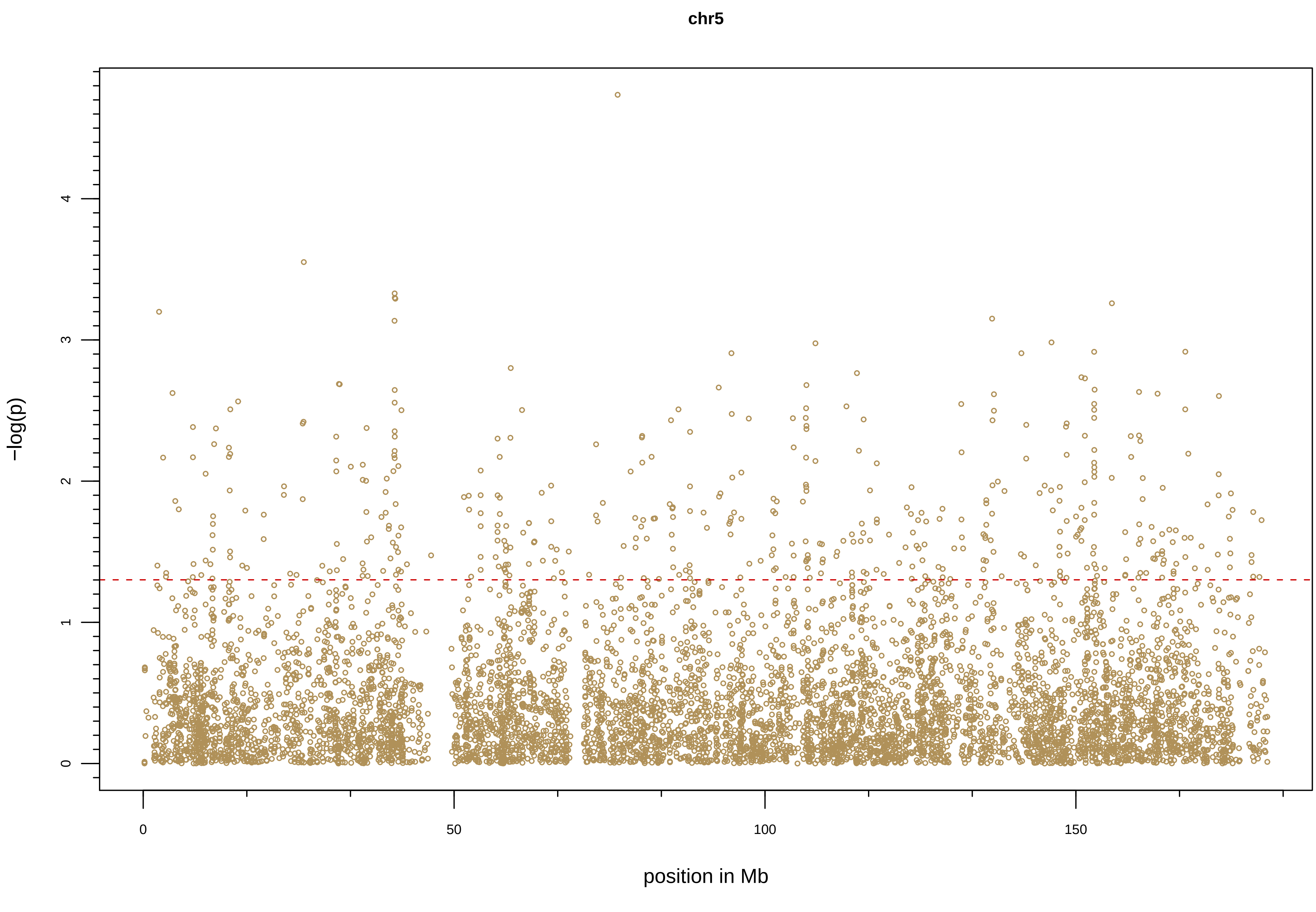
chr5
−log(p)
0
1
2
3
4
position in Mb
0
50
100
150

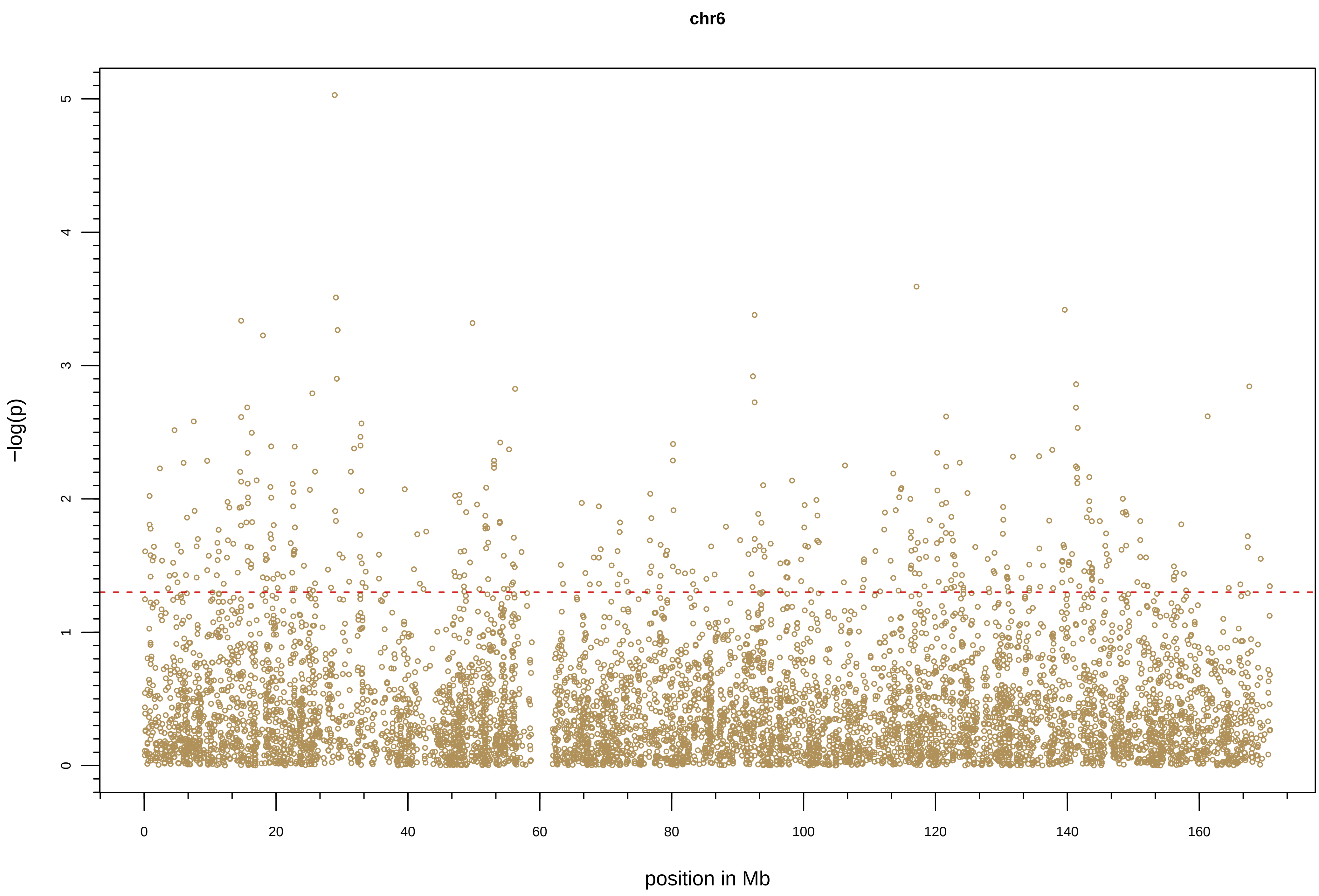

chr6
−log(p)
0
1
2
3
4
5
0
20
40
60
80
100
120
140
160
position in Mb

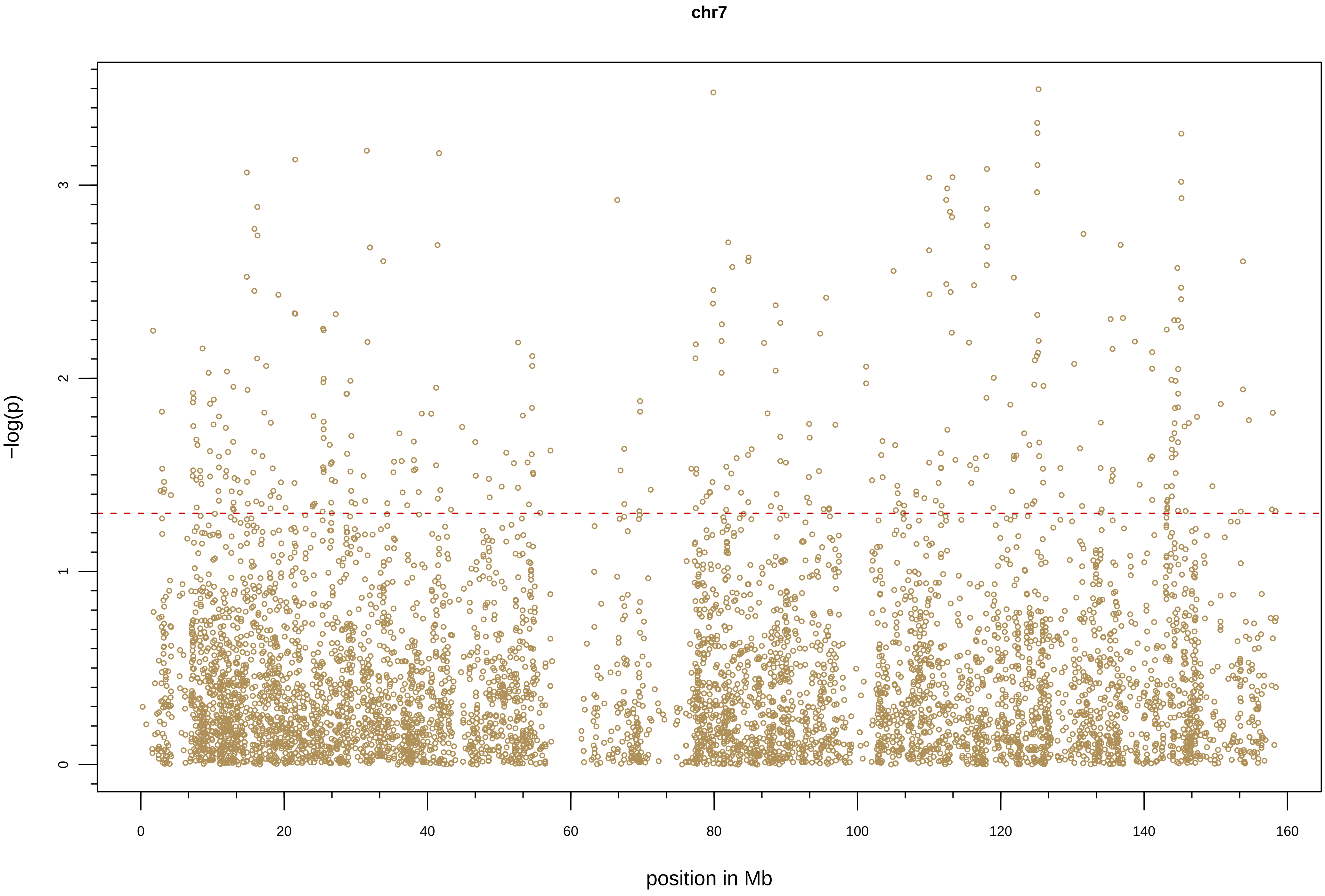

chr7
−log(p)
0
1
2
3
0
20
40
60
80
100
120
140
160
position in Mb

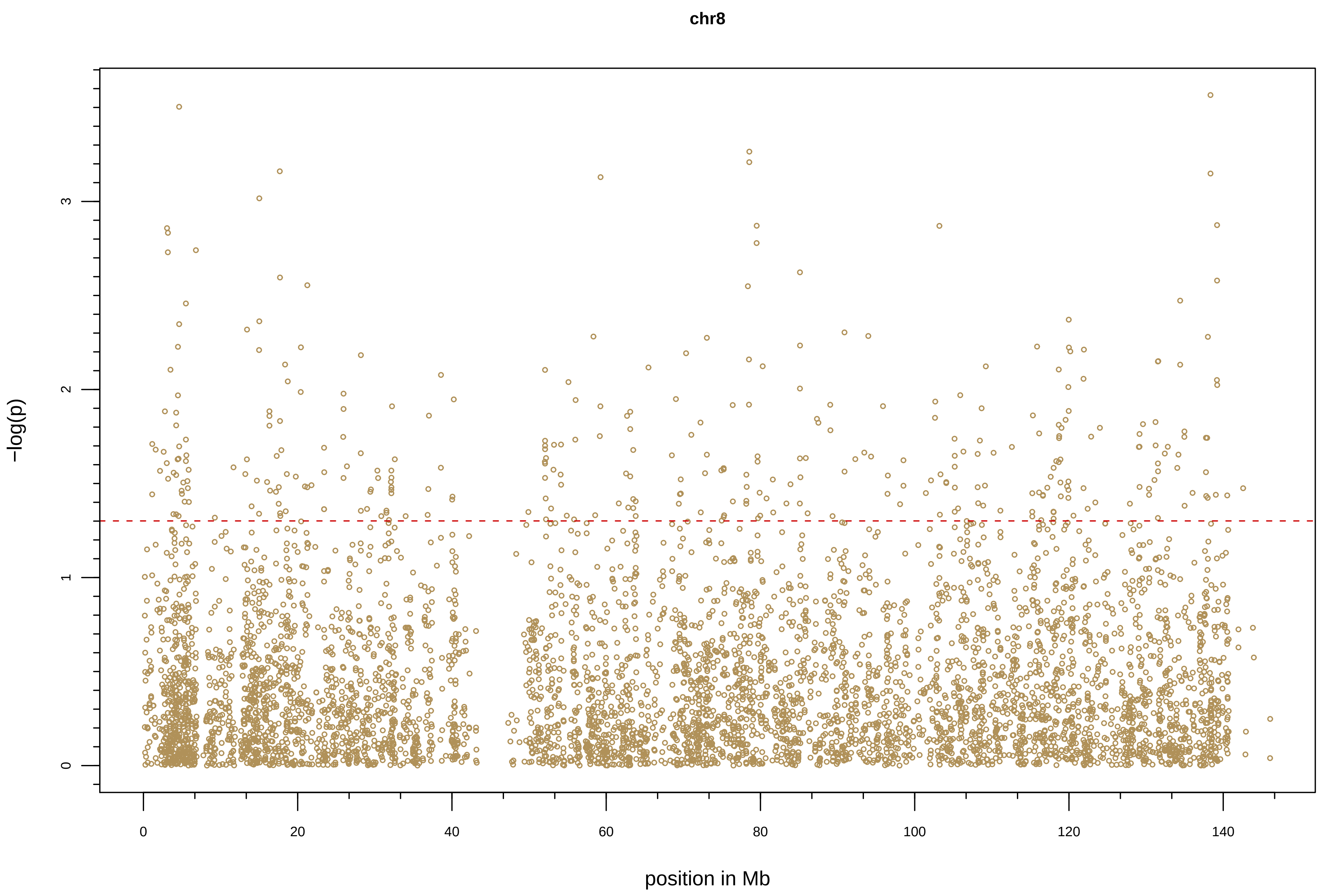
chr8
-log(p)
0
1
2
3
0
20
40
60
80
100
120
140
position in Mb

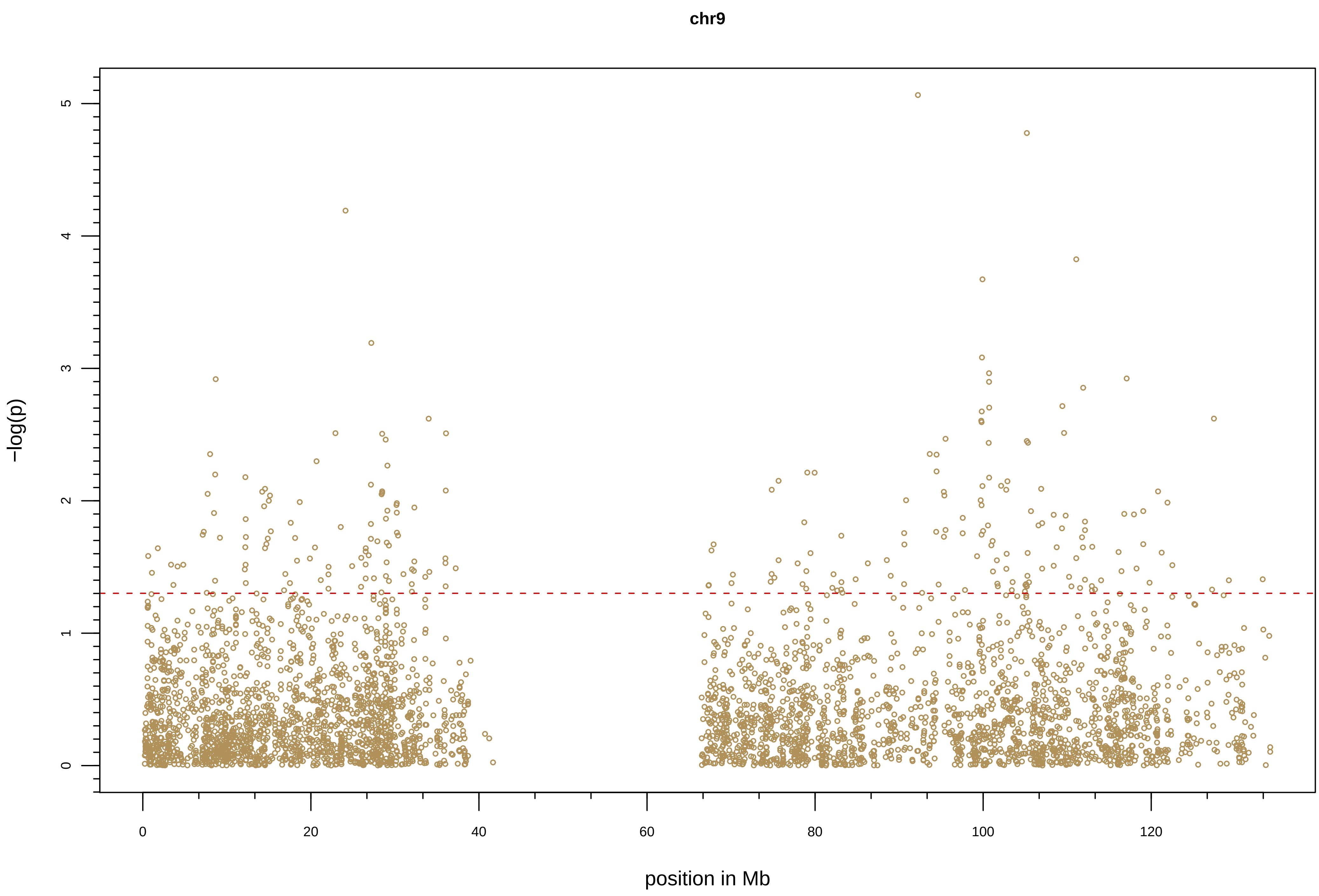

chr9
−log(p)
0
1
2
3
4
5
0
20
40
60
80
100
120
position in Mb

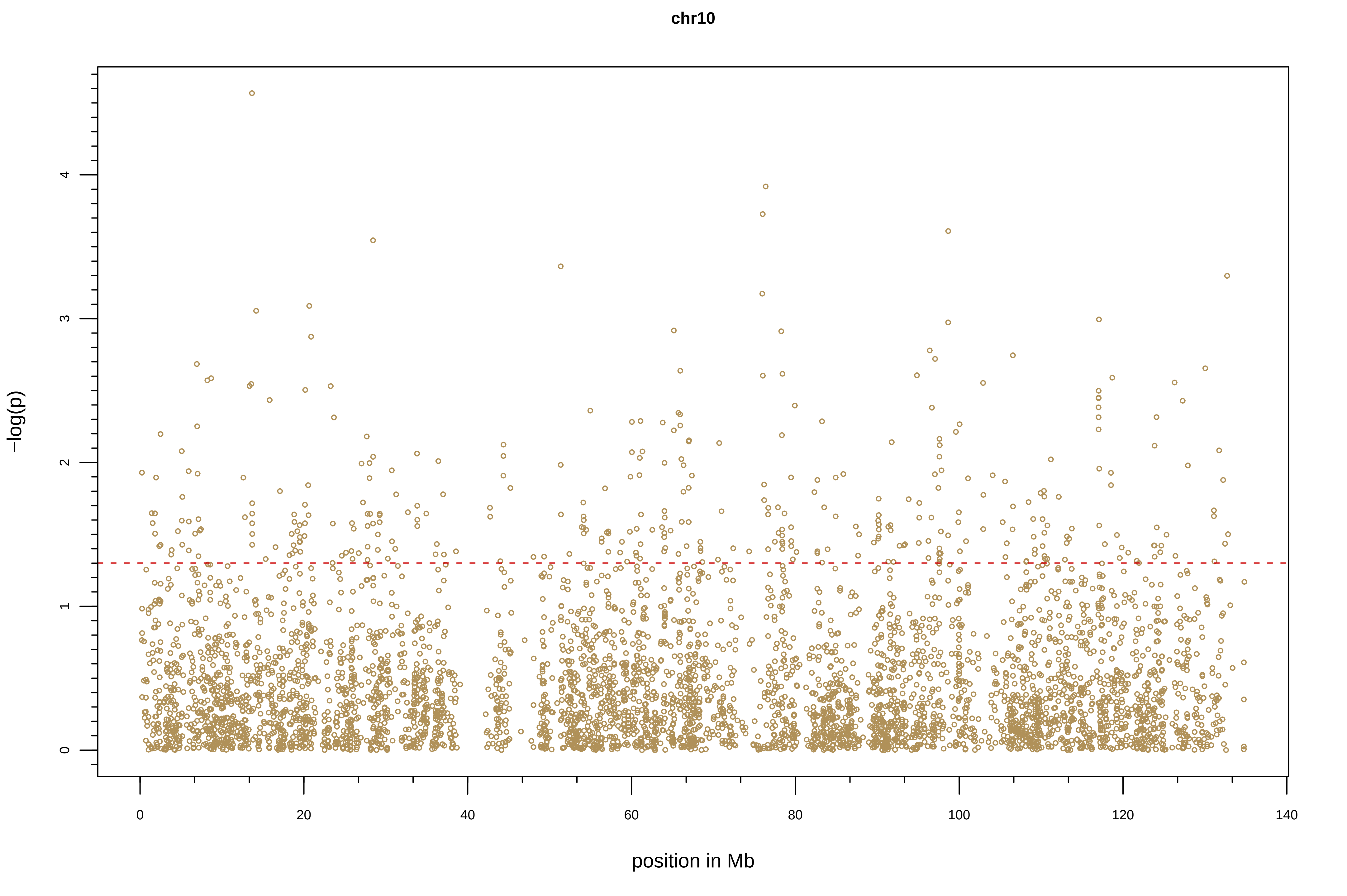
chr10
−log(p)
0
1
2
3
4
position in Mb
0
20
40
60
80
100
120
140

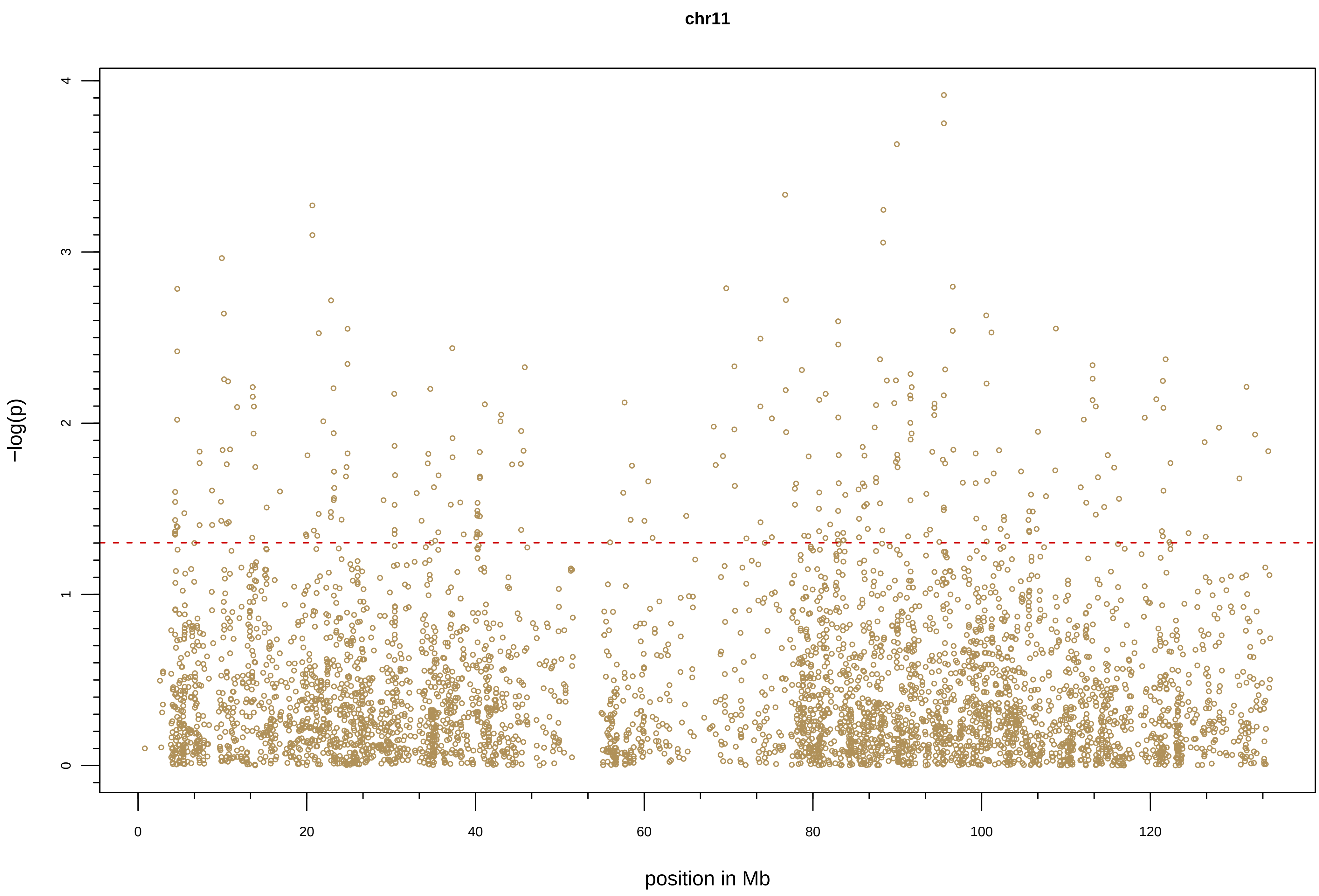
chr11
-log(p)
position in Mb
0
1
2
3
4
0
20
40
60
80
100
120

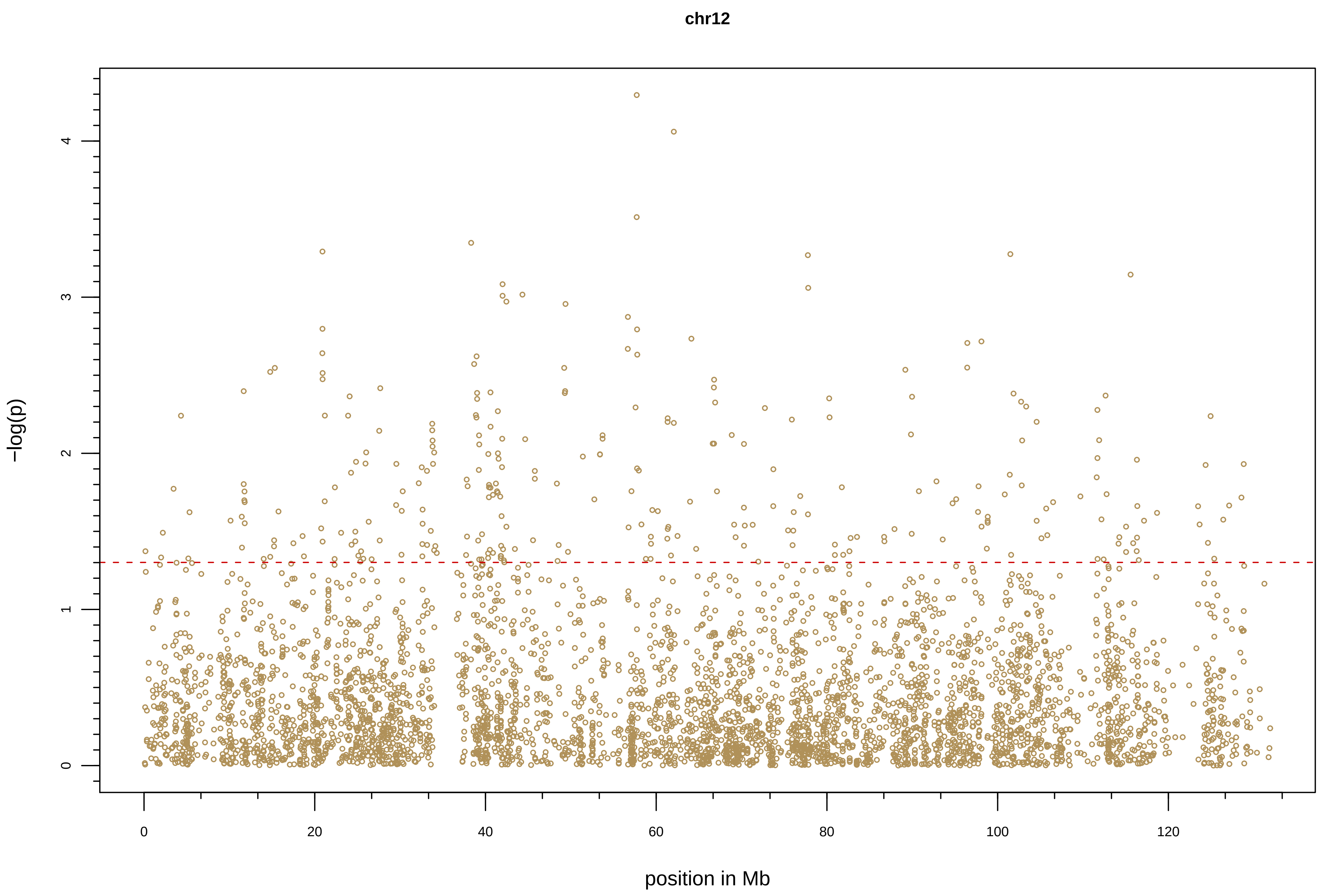
chr12
-log(p)
position in Mb
0
1
2
3
4
0
20
40
60
80
100
120

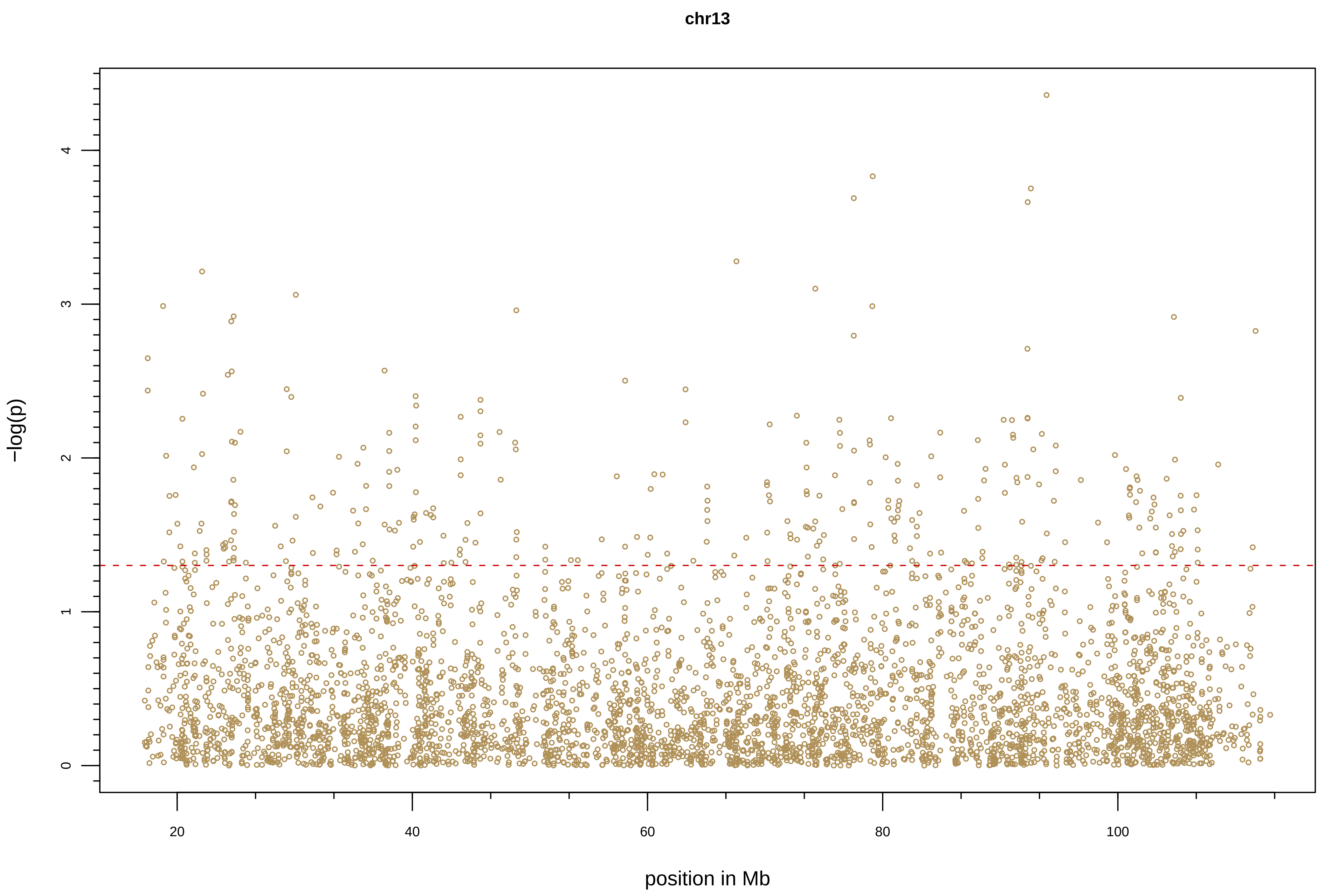
chr13
−log(p)
0
1
2
3
4
20
40
60
80
100
position in Mb

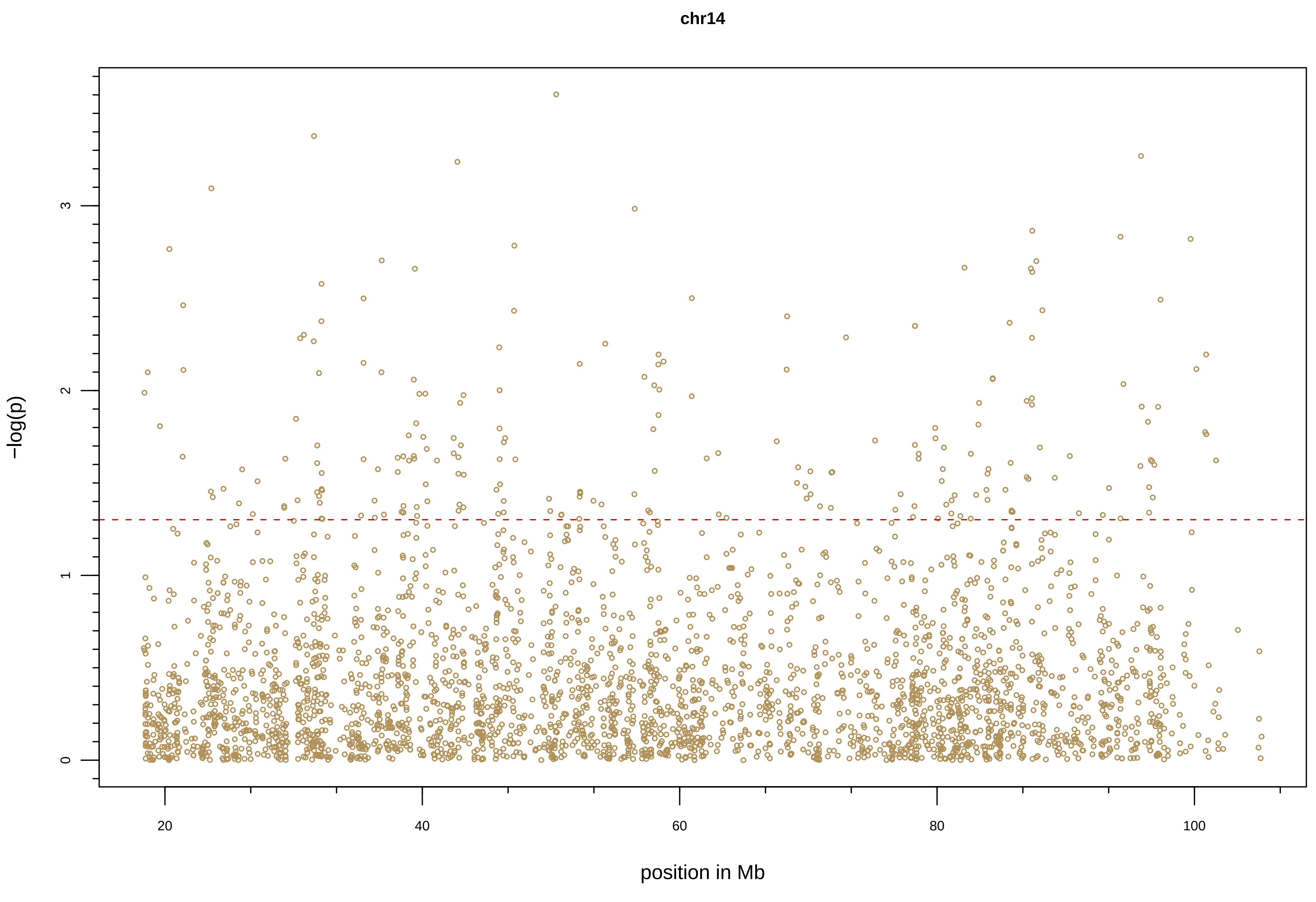
chr14
−log(p)
0
1
2
3
20
40
60
80
100
position in Mb

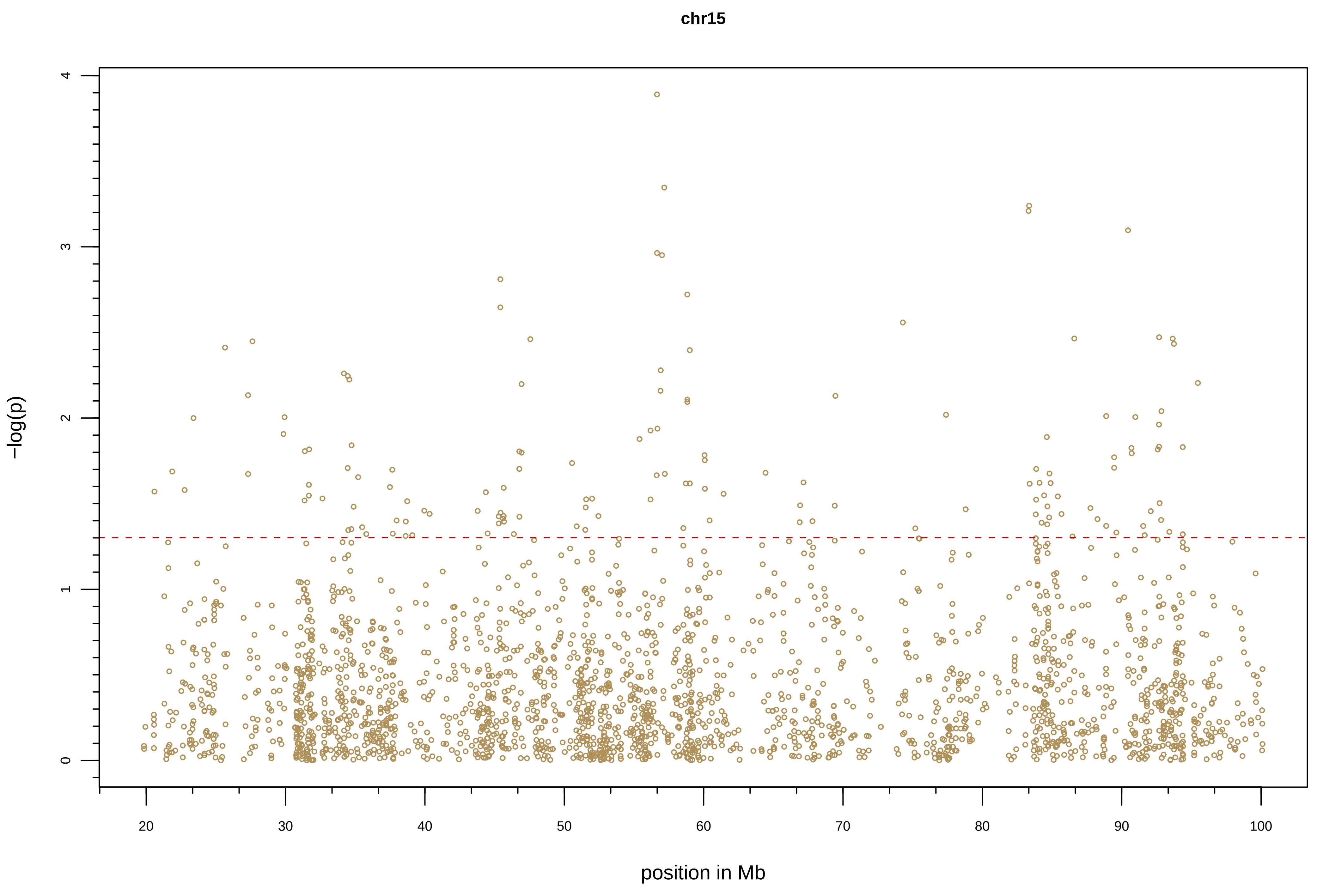
chr15
-log(p)
0
1
2
3
4
20
30
40
50
60
70
80
90
100
position in Mb

chr16

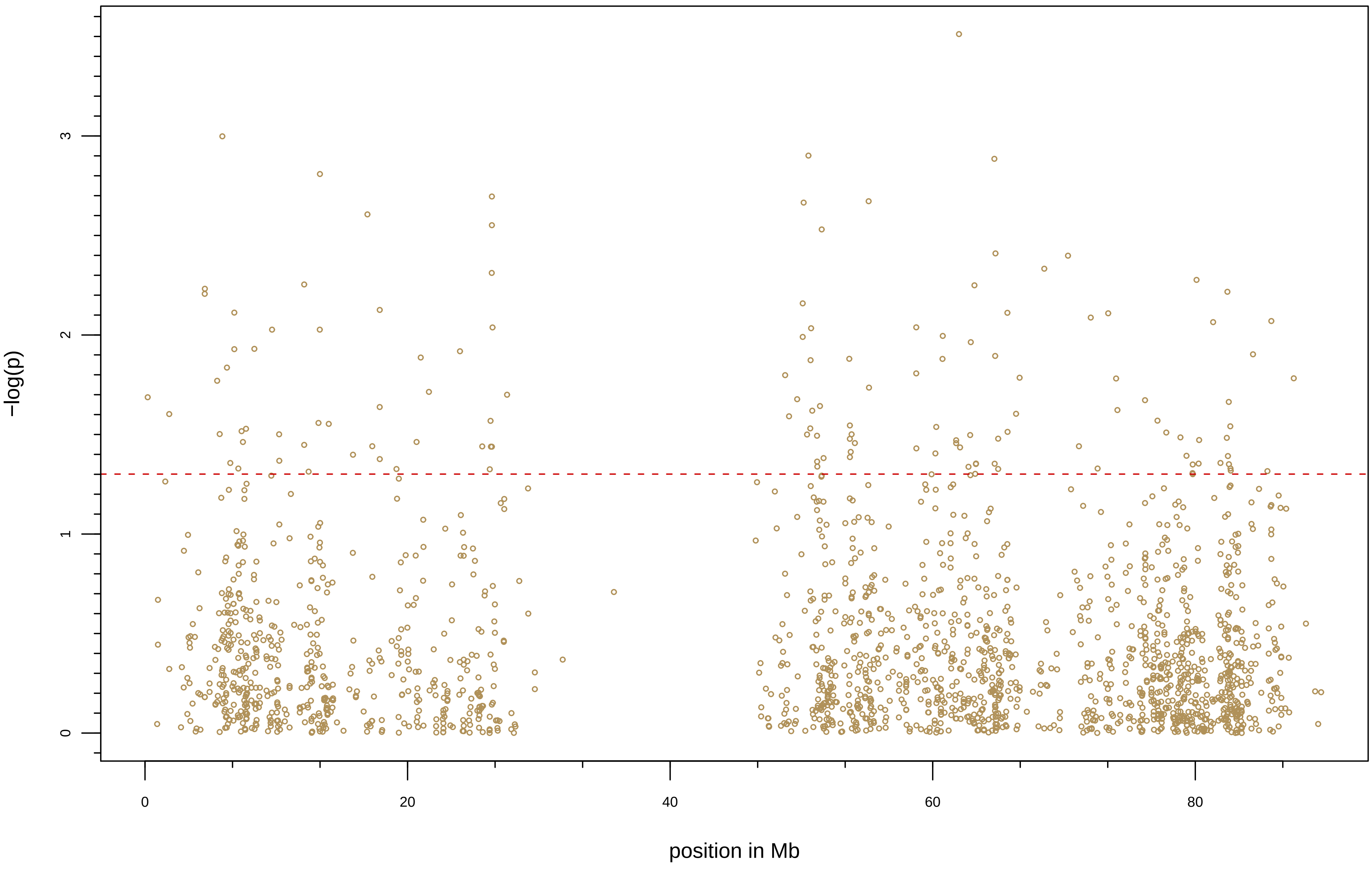

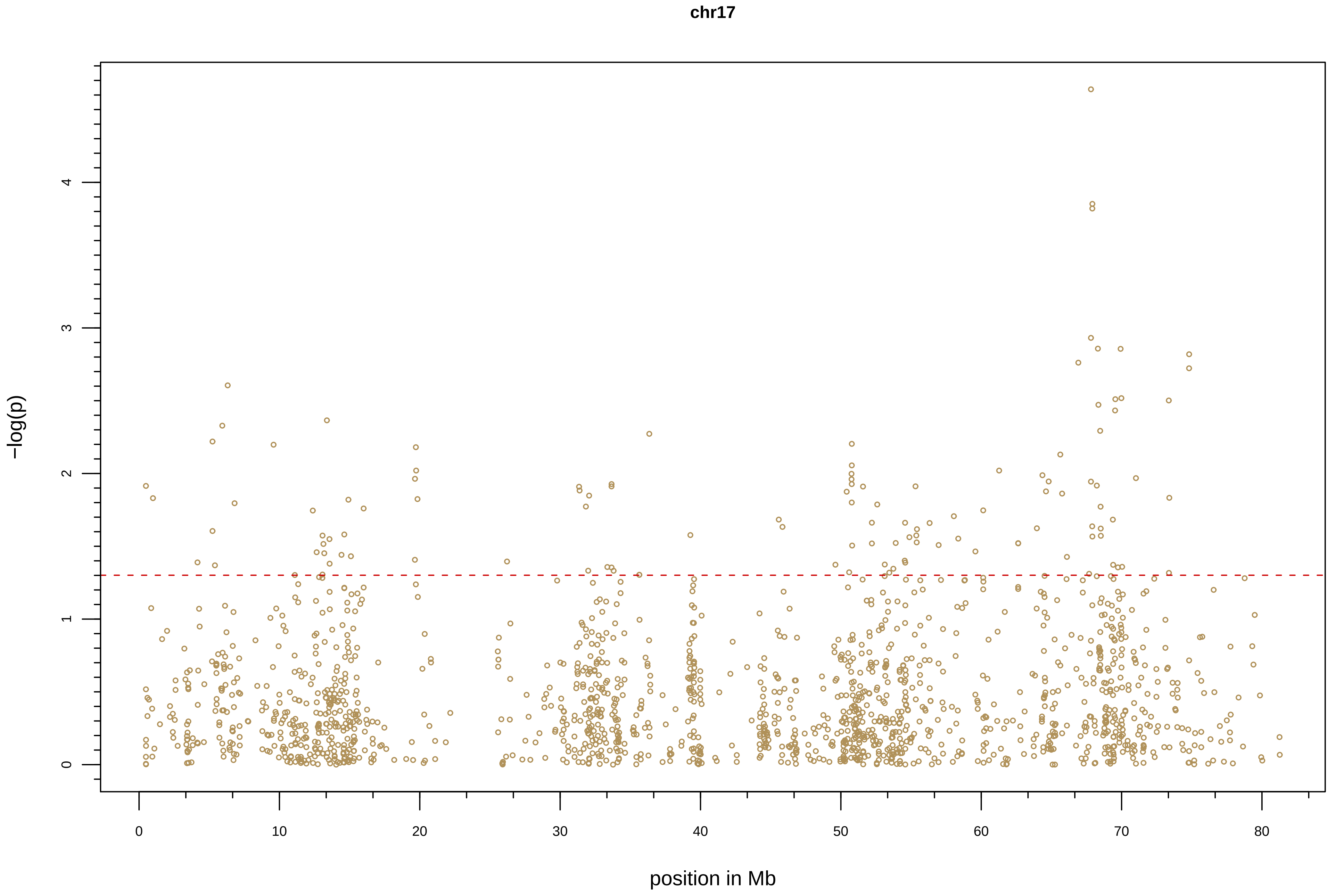
chr17
−log(p)
0
1
2
3
4
0
10
20
30
40
50
60
70
80
position in Mb

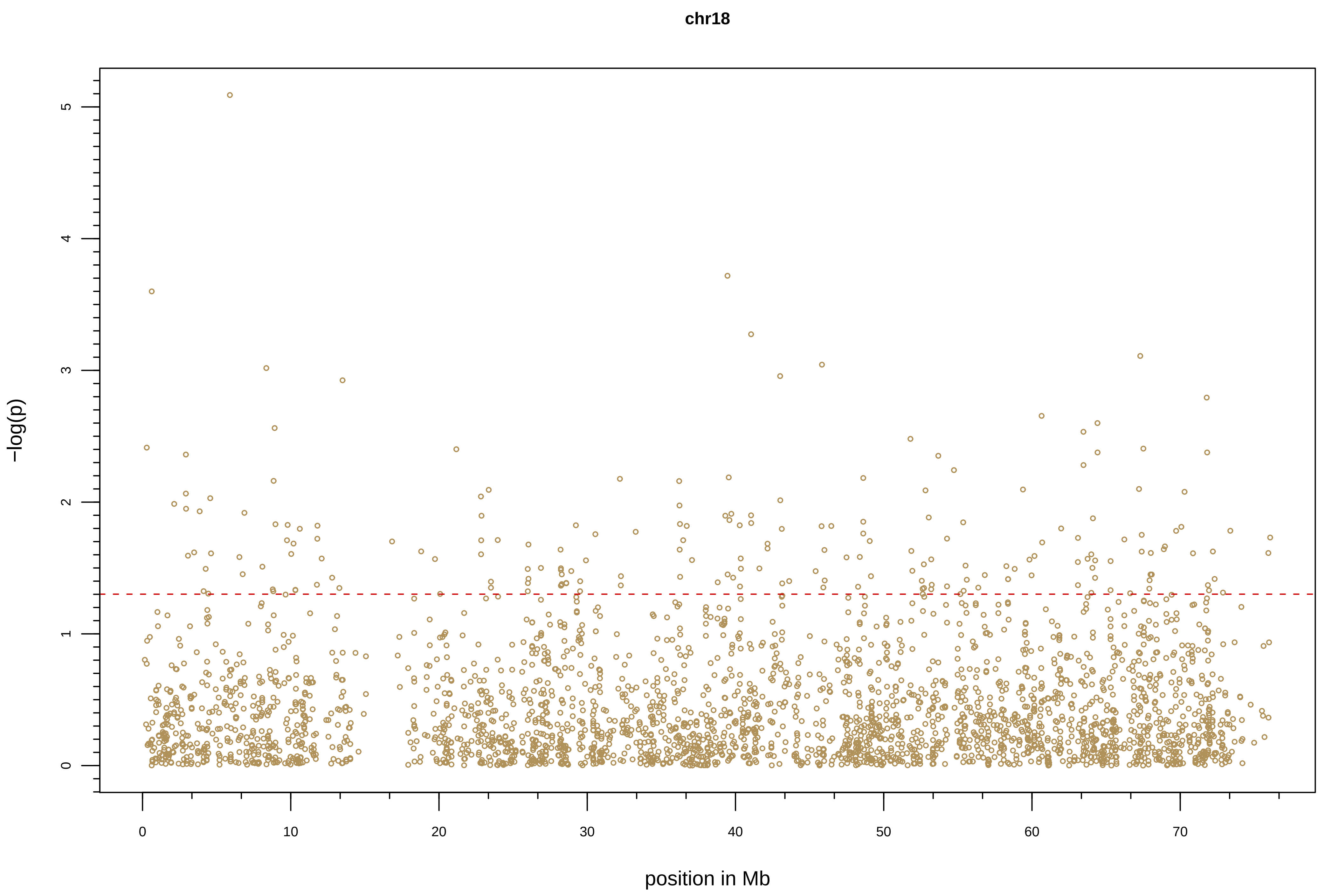
chr18
-log(p)
position in Mb
0
1
2
3
4
5
0
10
20
30
40
50
60
70

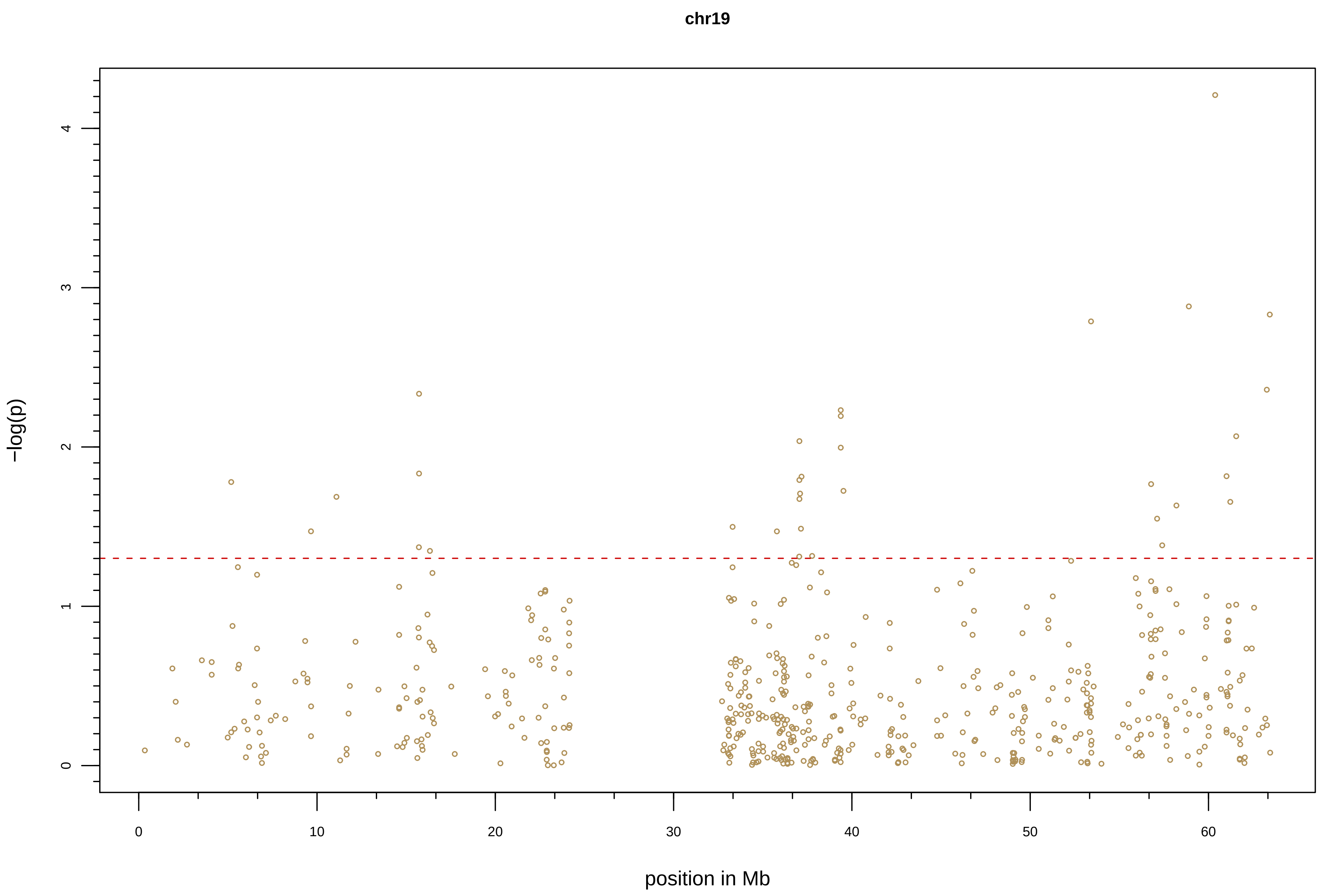
chr19
−log(p)
0
1
2
3
4
0
10
20
30
40
50
60
position in Mb

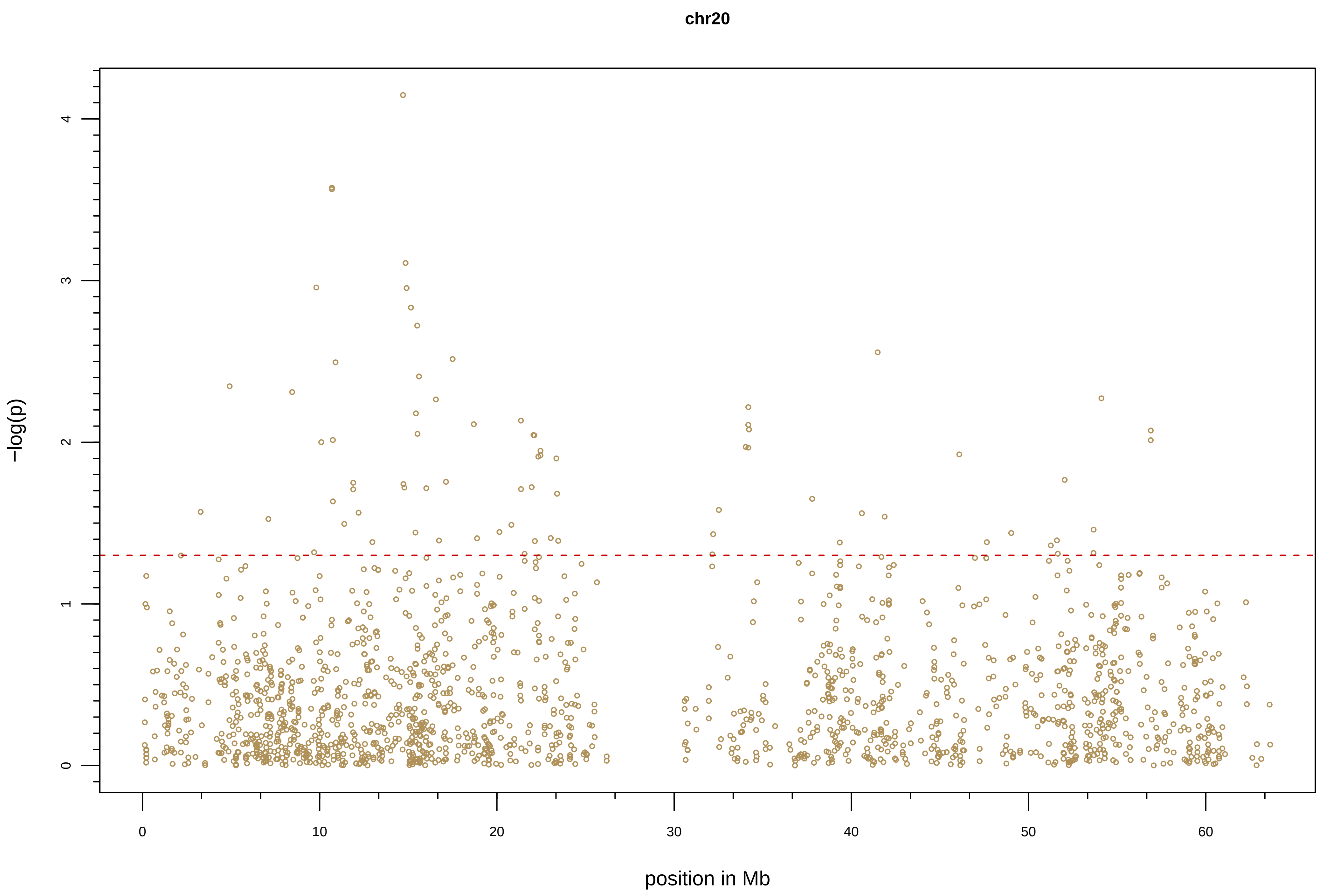

chr20
−log(p)
0
1
2
3
4
position in Mb
0
10
20
30
40
50
60

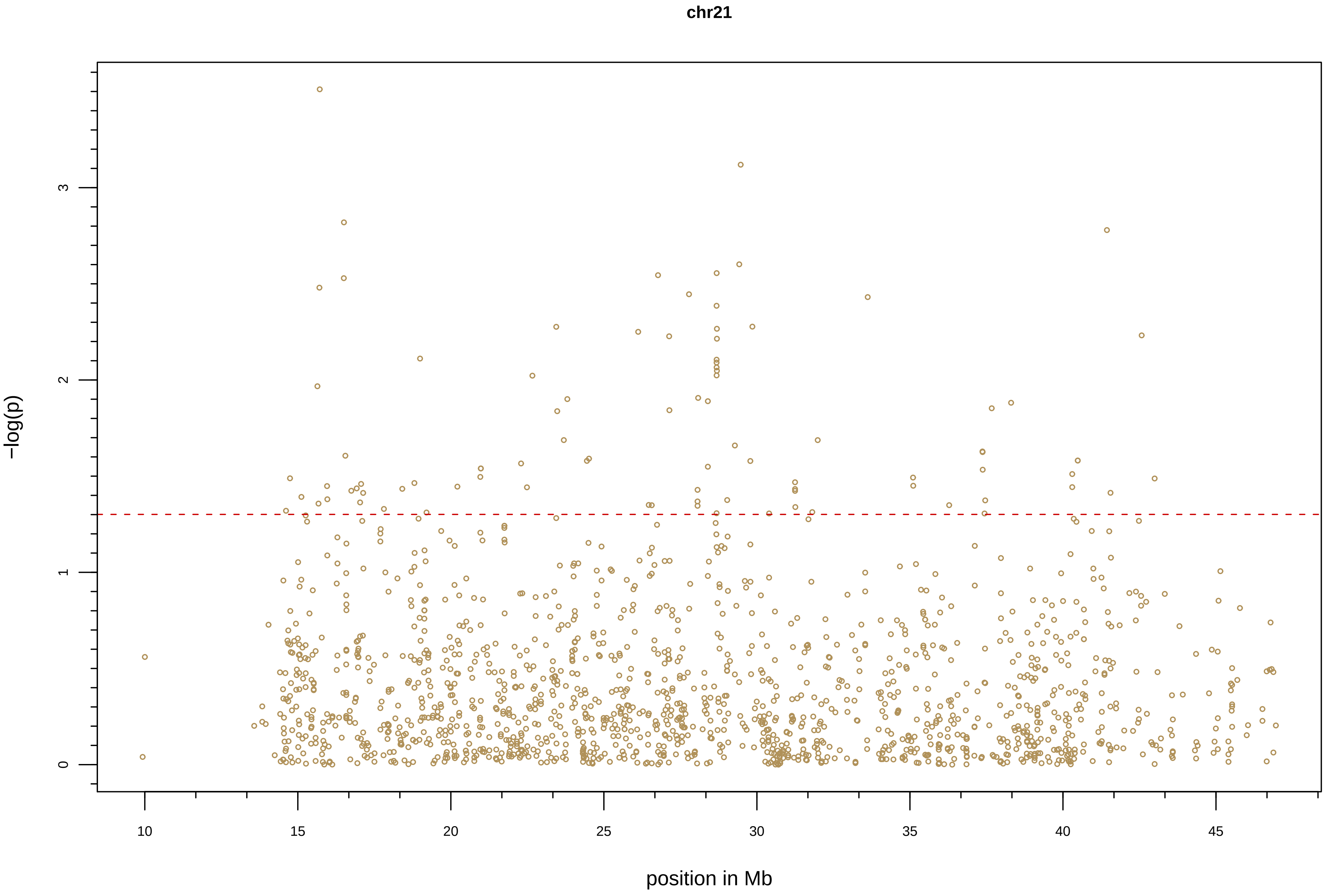
chr21
−log(p)
0
1
2
3
10
15
20
25
30
35
40
45
position in Mb

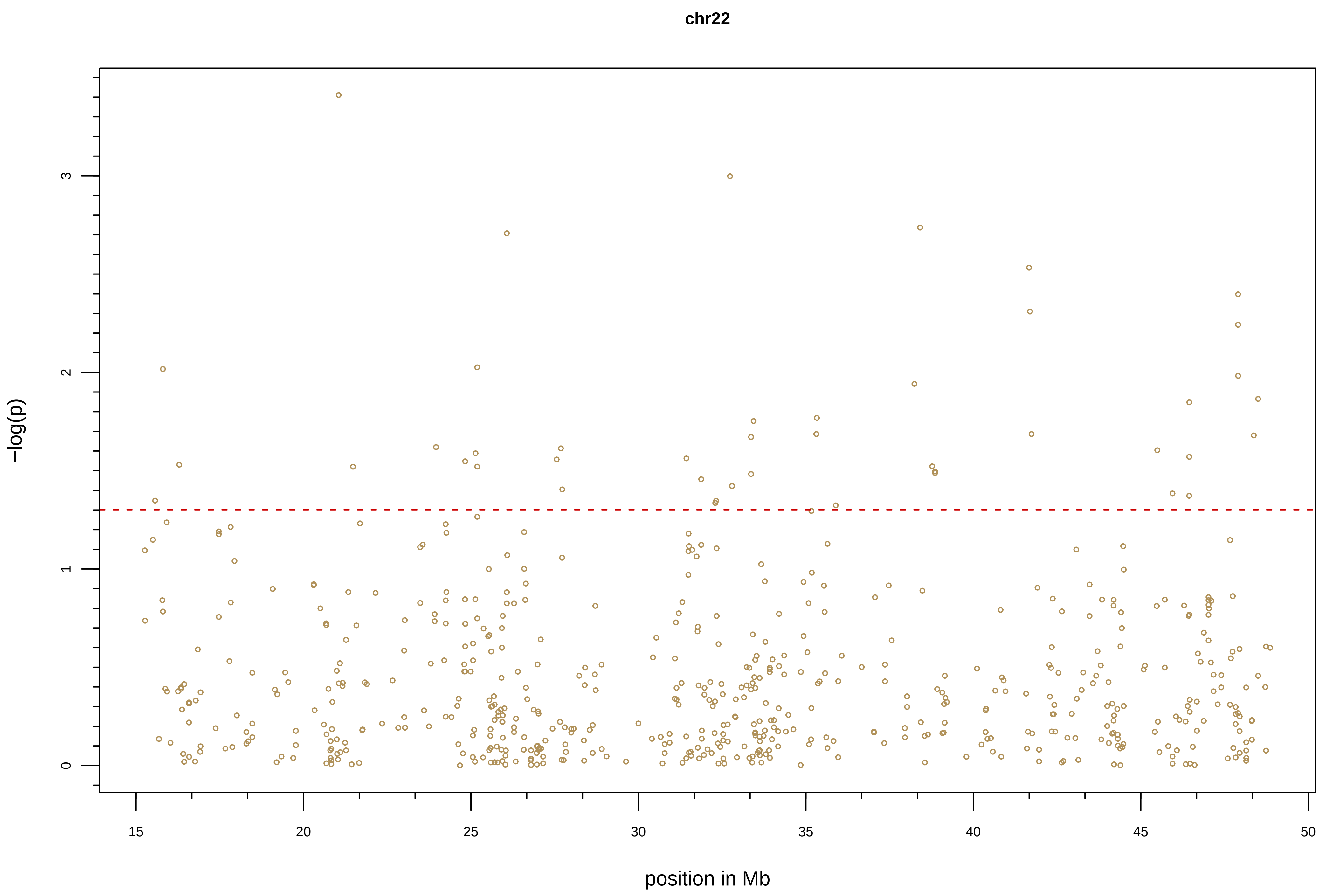

chr22
−log(p)
0
1
2
3
15
20
25
30
35
40
45
50
position in Mb

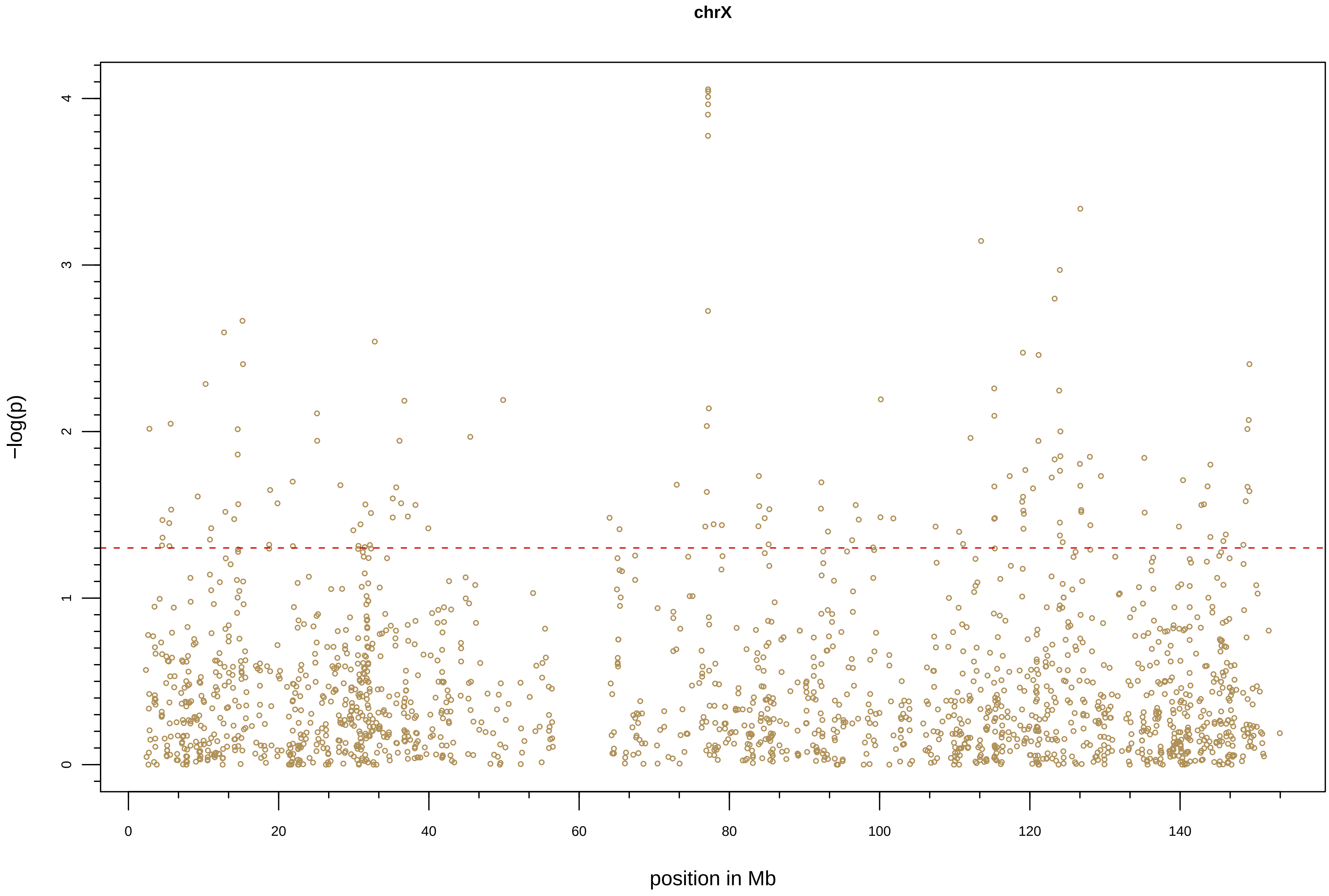

chrX
−log(p)
4
3
2
1
0
0
20
40
60
80
100
120
140
position in Mb
