# Supplementary material for: Systematic Association Mapping Identifies NELL1 as a Novel IBD Disease Gene
Source: PLoS One. 2007 Aug 8;2(8):e691. doi: 10.1371/journal.pone.0000691 (PMC1933598; doi:10.1371/journal.pone.0000691)
Supplement: Table S1 — Summary of the genome-wide association scan. Table (A) gives an overview of the SNP number and density per chromosome. Table (B) lists the distribution of the p-values in the genome-wide scan versus a randomized experiment, i.e. randomized affection status of individuals. CR: callrate in cases and controls, MAF: minor allele frequency in controls, pHWE: p-value for Hardy-Weinberg equilibrium in controls, QC: quality control, CCA: p-value of Chi2 test for alleles and genotypes (CCG), *excl. unmapped (0.12 MB PDF) [file pone.0000691.s009.pdf]

Supplementary Table 1: Summary of the genome-wide association scan. Table **(A)** gives an overview of the SNP number and density per chromosome. Table **(B)** lists the distribution of the p-values in the genome-wide scan versus a randomized experiment, i.e. randomized affection status of individuals. CR: callrate in cases and controls, MAF: minor allele frequency in controls, p_{HWE}: p-value for Hardy-Weinberg equilibrium in controls, QC: quality control, CC_A: p-value of χ^2 test for alleles and genotypes (CC_G), *excl. unmapped

A.

**Chr.**	**SNPs on chip**	**CR <90%**	**MAF = 1.0**	**p_{HWE} <0.01**	**SNPs passing QC* (%)**	**Avg. distance between SNPs [kbp]**	**Median distance between SNPs [kbp]**
1	9,197	495	899	405	7,398 (0.80)	32.9	11.8
2	10,352	592	883	530	8,347 (0.81)	29.1	11.1
3	7,813	492	683	367	6,271 (0.80)	31.7	11.9
4	8,595	485	873	475	6,762 (0.79)	28.3	11.5
5	8,371	567	649	432	6,723 (0.80)	26.9	10.8
6	8,089	621	562	408	6,498 (0.80)	26.3	10.8
7	7,069	535	556	375	5,603 (0.79)	28.2	10.3
8	6,975	463	662	374	5,476 (0.79)	26.7	9.2
9	4,801	280	396	279	3,846 (0.80)	34.8	11.4
10	5,692	290	459	271	4,672 (0.82)	28.8	11.5
11	5,367	274	495	305	4,293 (0.80)	31.1	11.8
12	5,263	335	420	287	4,221 (0.80)	31.2	11.6
13	5,241	334	494	292	4,121 (0.79)	23.2	10.4
14	4,015	259	345	224	3,187 (0.79)	27.3	11.4
15	3,032	177	326	182	2,347 (0.77)	34.2	11.8
16	2,380	116	241	125	1,898 (0.80)	47.1	12.1
17	1,963	95	157	103	1,608 (0.82)	50.3	16.6
18	3,570	181	295	188	2,906 (0.81)	26.1	10.7
19	688	33	52	35	568 (0.83)	111.3	29.6
20	2,090	123	160	98	1,709 (0.82)	37.2	15.3
21	1,913	117	146	114	1,536 (0.80)	24.1	9.3
22	761	42	77	34	608 (0.80)	55.4	23.3
X	2,334	129	358	58	1,789 (0.77)	84.4	36.7
**Σ**	**115,571**	**7,035**	**10,188**	**5,961**	**92,387 (0.80)**	**Avg. 38.1**	**Avg. 14.0**

B.

p-value	Real GWS CC_A	Real GWS CC_G	Randomized GWS CC_A	Randomized GWS CC_G
**> 0.05**	86,564	87,196	87,767	88,487
	93.697%	94.381%	94.999%	95.779%
**$0.05 \geq p > 10^{-2}$**	4,411	4,124	3,649	3,196
	4.774%	4.464%	3.950%	3.459%
**$10^{-2} \geq p > 10^{-3}$**	1,245	931	847	629
	1.348%	1.008%	0.917%	0.681%
**$10^{-3} \geq p > 10^{-4}$**	147	115	104	71
	0.159%	0.124%	0.113%	0.077%
**$10^{-4} \geq p > 10^{-5}$**	14	13	19	4
	0.015%	0.014%	0.021%	0.004%
**$p \leq 10^{-5}$**	6	8	1	0
	0.006%	0.009%	0.001%	0.000%
