# Supplementary material for: Systematic Association Mapping Identifies NELL1 as a Novel IBD Disease Gene
Source: PLoS One. 2007 Aug 8;2(8):e691. doi: 10.1371/journal.pone.0000691 (PMC1933598; doi:10.1371/journal.pone.0000691)
Supplement: Table S2 — Top 200 CD-associated SNPs, ranked with respect to p-values obtained in an allele- (pCCA) or genotype-based (pCCG) case-control comparison in panel A. Also included are pCCA, pCCG, and the transmission disequilibrium test results (pTDT) for the replication panel B. Nucleotide positions refer to NCBI build 35. Markers with p< = 0.05 in either the case-control analysis or the transmission disequilibrium test (TDT) in replication panel B are highlighted in bold italics. SNPs with a significant result in both panel B tests are additionally marked by grey shading. (0.15 MB PDF) [file pone.0000691.s010.pdf]

Supplementary Table 2: Top 200 CD-associated SNPs, ranked with respect to p-values obtained in an allele- (p_{CCA}) or genotype-based (p_{CCG}) case-control comparison in panel A. Also included are p_{CCA}, p_{CCG}, and the transmission disequilibrium test results (p_{TDT}) for the replication panel B. Nucleotide positions refer to NCBI build 35. Markers with $p \leq 0.05$ in either the case-control analysis or the transmission disequilibrium test (TDT) in replication panel B are highlighted in bold italics. SNPs with a significant result in both panel B tests are additionally marked by grey shading.

SNP Information						Screening (Panel A)					Replication (Panel B)					
Rank	Affymetrix SNP ID	dbSNP ID	Chr.	Position	Locus	MAF_{co}	MAF_{ca}	P_{CCA}	P_{CCG}	OR (95% CI)	MAF_{co}	MAF_{ca}	P_{CCA}	P_{CCG}	OR (95% CI)	P_{TDT}
1	***1741384***	***rs2076756***	***16***	***49,314,382***	***CARD15, intron***	***0.26***	***0.43***	***1.93E-13***	***2.04E-12***	***2.1 (1.58-2.80)***	***0.27***	***0.41***	***6.80E-20***	***1.39E-21***	***1.71 (1.42-2.05)***	***5.90E-08***
2	1683804	rs10513059	3	140,475,116	MRPS22, intron	0.13	0.06	2.07E-06	1.56E-05	0.39 (0.26-0.58)	0.12	0.10	0.15	0.14	0.83 (0.67-1.03)	0.93
3	1700797	rs200354	14	98,445,074	BCL11B, downstream	0.17	0.12	0.0032	2.22E-06	0.5 (0.36-0.70)	0.15	0.14	0.59	0.39	0.92 (0.75-1.12)	1.00
4	1668246	rs2009530	19	53,411,500	CARD8, intron	0.07	0.11	0.0016	4.92E-06	1.24 (0.82-1.87)	0.09	0.09	0.73	0.83	1.03 (0.81-1.30)	0.52
5	1680746	rs10484543	6	28,899,181	ZNF452, upstream	0.25	0.16	9.36E-06	3.56E-05	0.5 (0.37-0.68)	0.21	0.19	0.16	0.11	0.84 (0.70-1.01)	0.066
6	1646788	rs10507063	12	94,769,766	FLJ40089, intron	0.11	0.09	0.084	1.19E-05	0.55 (0.37-0.82)	0.11	0.10	0.13	0.060	0.89 (0.72-1.11)	0.70
7	1664649	rs4978664	9	106,899,483	RAD23B, upstream	0.43	0.32	1.67E-05	0.00013	0.59 (0.44-0.79)	0.39	0.38	0.57	0.33	0.9 (0.75-1.08)	0.66
8	1728972	rs7674505	4	134,894,593	SLC7A11, downstream	0.39	0.29	2.63E-05	0.00014	0.61 (0.46-0.82)	0.37	0.36	0.56	0.38	1.01 (0.84-1.20)	0.13
9	1670981	rs2478126	10	13,699,744	PRPF18, intron	0.08	0.03	2.70E-05	0.00012	0.34 (0.20-0.57)	0.06	0.07	0.30	0.29	1.18 (0.91-1.54)	0.83
10	1716065	rs2660727	4	134,912,460	SLC7A11, downstream	0.39	0.29	3.95E-05	0.00020	0.62 (0.47-0.82)	0.37	0.36	0.49	0.21	1.01 (0.85-1.21)	0.076
11	1741482	rs7324781	13	95,032,754	DZIP1, intron	0.49	0.59	4.37E-05	0.00030	1.82 (1.29-2.57)	0.46	0.45	0.46	0.75	0.94 (0.77-1.14)	0.25
12	1717655	rs6659639	1	15,185,462	BC036877, intron	0.29	0.36	0.0018	5.17E-05	1.21 (0.91-1.61)	0.31	0.32	0.68	0.86	1.02 (0.85-1.22)	0.050
13	1749262	rs1590734	10	117,348,909	ATRNL1, intron	0.46	0.53	0.0032	5.57E-05	1.08 (0.79-1.48)	0.47	0.48	0.30	0.11	1.21 (0.99-1.47)	0.84
14	***1700613***	***rs3859540***	***19***	***60,373,858***	***SYT5, downstream***	***0.12***	***0.19***	***6.18E-05***	***0.00039***	***1.88 (1.37-2.58)***	***0.15***	***0.17***	***0.036***	***0.12***	***1.21 (1.00-1.47)***	***0.27***
15	***1692451***	***rs6074780***	***20***	***14,654,879***	***BC036403, intron***	***0.27***	***0.36***	***7.12E-05***	***0.00037***	***1.59 (1.20-2.11)***	***0.31***	***0.34***	***0.047***	***0.084***	***1.13 (0.94-1.34)***	***0.64***
16	1717307	rs10487283	7	102,065,164	FBXL13, intron	0.20	0.17	0.16	8.60E-05	0.65 (0.47-0.89)	0.17	0.16	0.37	0.086	0.87 (0.71-1.05)	0.42
17	1684094	rs10509992	10	117,339,994	ATRNL1, intron	0.46	0.53	0.0036	8.67E-05	1.08 (0.79-1.49)	0.47	0.49	0.21	0.097	1.22 (1.00-1.49)	1.00
18	1747741	rs483952	10	100,373,989	HPSE2, intron	0.18	0.13	0.0054	8.73E-05	0.54 (0.39-0.76)	0.19	0.19	0.59	0.71	0.93 (0.78-1.13)	0.79
19	1752489	rs10494251	1	144,248,710	BCL9, intron	0.08	0.04	9.83E-05	0.00037	0.39 (0.24-0.63)	0.06	0.06	0.32	0.23	0.89 (0.68-1.17)	0.59
20	1666299	rs1559865	18	13,493,887	C18orf1, intron	0.30	0.38	0.0012	0.00010	1.25 (0.94-1.66)	0.31	0.34	0.052	0.15	1.17 (0.98-1.39)	0.30
21	1704045	rs10509357	10	76,699,180	ZNF503, downstream	0.25	0.34	0.00012	0.00039	1.54 (1.16-2.04)	0.27	0.28	0.34	0.091	1.16 (0.97-1.38)	0.52
22	1724833	rs1074742	11	95,480,084	MAML2, intron	0.43	0.33	0.00012	0.00078	0.59 (0.44-0.79)	0.39	0.39	0.82	0.98	0.98 (0.82-1.18)	0.56
23	1722142	rs10517460	4	37,789,649	BC050321, intron	0.11	0.07	0.011	0.00013	0.51 (0.34-0.76)	0.11	0.11	0.60	0.85	0.95 (0.76-1.18)	0.26
24	1750774	rs10493084	1	38,833,246	POU3F1, upstream	0.01	0.04	0.00014	0.0011	3.72 (1.75-7.92)	0.03	0.03	0.90	0.38	1.07 (0.72-1.59)	0.88
25	1728010	rs747066	9	112,777,901	ZFP37, downstream	0.34	0.43	0.00015	0.00038	1.78 (1.33-2.39)	0.36	0.36	0.97	0.079	0.9 (0.75-1.08)	0.86
26	***1717433***	***rs925084***	***4***	***134,887,991***	***SLC7A11, downstream***	***0.40***	***0.31***	***0.00016***	***0.00057***	***0.64 (0.48-0.86)***	***0.38***	***0.37***	***0.25***	***0.27***	***0.96 (0.80-1.15)***	***0.021***
27	1749044	rs1590736	10	117,348,799	ATRNL1, intron	0.46	0.53	0.0035	0.00016	1.11 (0.81-1.51)	0.45	0.48	0.16	0.13	1.22 (1.00-1.48)	0.87
28	1727572	rs1333261	13	93,704,104	GPC6, intron	0.30	0.21	0.00018	0.00048	0.57 (0.43-0.76)	0.26	0.24	0.22	0.46	0.91 (0.76-1.08)	0.11
29	1724641	rs10501843	11	95,479,768	MAML2, intron	0.33	0.24	0.00018	0.0013	0.62 (0.47-0.82)	0.29	0.28	0.48	0.55	0.97 (0.82-1.16)	0.73
30	1699729	rs2367129	3	194,078,009	LOC151963, intron	0.21	0.29	0.00019	0.00096	1.61 (1.21-2.14)	0.28	0.27	0.96	0.91	1.01 (0.85-1.21)	0.76
31	1690976	rs7092435	10	76,340,317	MYST4, intron	0.09	0.15	0.00019	0.00043	1.99 (1.41-2.82)	0.08	0.09	0.79	0.22	1.09 (0.85-1.38)	0.56
32	1699663	rs605216	13	78,642,856	C13orf10, downstream	0.10	0.05	0.00020	0.0010	0.46 (0.31-0.71)	0.08	0.08	0.84	0.11	0.93 (0.73-1.19)	0.84
33	1666587	rs7700170	4	134,958,596	SLC7A11, downstream	0.41	0.32	0.00021	0.0011	0.66 (0.50-0.88)	0.38	0.38	0.62	0.31	1.03 (0.86-1.23)	0.095
34	***1741147***	***rs4743487***	***9***	***101,615,858***	***CYLC2, upstream***	***0.26***	***0.18***	***0.00021***	***0.0010***	***0.6 (0.45-0.80)***	***0.24***	***0.21***	***0.015***	***0.047***	***0.83 (0.69-0.99)***	***0.80***
35	***1642811***	***rs2925757***	***2***	***160,926,676***	***ITGB6, upstream***	***0.15***	***0.22***	***0.00021***	***0.0014***	***1.68 (1.24-2.26)***	***0.16***	***0.19***	***0.0035***	***0.016***	***1.3 (1.08-1.58)***	***1.00***
36	1688004	rs2660693	4	134,977,216	SLC7A11, downstream	0.30	0.22	0.00022	0.0012	0.6 (0.45-0.80)	0.27	0.27	0.87	0.98	0.98 (0.82-1.17)	0.48
37	1698524	rs10501720	11	89,902,331	CHORDC1, upstream	0.13	0.20	0.00023	0.00075	1.82 (1.33-2.49)	0.17	0.17	0.82	0.69	1 (0.82-1.21)	0.43
38	1712739	rs853727	4	150,192,617	NR3C2, upstream	0.21	0.29	0.00024	0.0011	1.64 (1.23-2.17)	0.26	0.25	0.68	0.85	0.95 (0.80-1.14)	0.58
39	***1753314***	***rs793515***	***10***	***98,978,459***	***ARHGAP19, intron***	***0.34***	***0.25***	***0.00025***	***0.0010***	***0.6 (0.45-0.79)***	***0.31***	***0.30***	***0.65***	***0.89***	***0.97 (0.82-1.16)***	***0.0020***
40	1675357	rs10498440	14	51,483,323	GNG2, intron	0.47	0.38	0.00025	0.00082	0.58 (0.43-0.79)	0.42	0.44	0.12	0.19	1.19 (0.99-1.44)	0.50
41	1666766	rs10502288	18	621,435	CLUL1, intron	0.36	0.27	0.00025	0.0013	0.6 (0.46-0.80)	0.30	0.29	0.69	0.66	0.94 (0.79-1.12)	0.076
42	1656805	rs2804200	10	117,340,287	ATRNL1, intron	0.46	0.53	0.0048	0.00026	1.1 (0.80-1.50)	0.47	0.49	0.23	0.12	1.21 (1.00-1.48)	0.79
43	1697779	rs6077888	20	10,640,989	JAG1, upstream	0.48	0.57	0.00027	0.00082	1.78 (1.26-2.53)	0.48	0.48	0.57	0.21	0.87 (0.71-1.06)	0.61
44	1685926	rs7681167	4	76,518,401	DKFZP564O0823, downstream	0.19	0.12	0.00027	0.0012	0.59 (0.43-0.80)	0.16	0.16	0.89	0.72	0.99 (0.81-1.20)	0.35
45	1738725	rs249721	5	115,050,156	CDO1, downstream	0.18	0.13	0.0061	0.00027	0.56 (0.41-0.78)	0.19	0.17	0.067	0.20	0.85 (0.70-1.02)	0.19
46	1708793	rs973542	20	10,640,559	JAG1, upstream	0.48	0.57	0.00027	0.00082	1.79 (1.26-2.54)	0.48	0.48	0.57	0.21	0.87 (0.71-1.06)	0.61
47	1510136	rs1342705	1	144,479,072	GJA5, upstream	0.22	0.15	0.00027	0.00042	0.55 (0.40-0.74)	0.21	0.19	0.12	0.28	0.87 (0.72-1.05)	0.73
48	1716967	rs2928266	5	25,817,370	CDH10, upstream	0.15	0.09	0.00028	0.0019	0.56 (0.39-0.78)	0.15	0.13	0.23	0.37	0.87 (0.71-1.06)	0.39
49	***1673372***	***rs7644124***	***3***	***135,058,563***	***RAB6B, intron***	***0.30***	***0.39***	***0.00029***	***0.00084***	***1.6 (1.20-2.14)***	***0.34***	***0.33***	***0.28***	***0.18***	***0.86 (0.72-1.03)***	***0.021***
50	1699331	rs3764008	12	20,905,406	SLCO1B3, intron	0.16	0.10	0.0016	0.00030	0.52 (0.37-0.74)	0.16	0.16	0.96	1.00	0.99 (0.82-1.20)	0.47
51	1650842	rs10490876	3	176,634,491	NLGN1, downstream	0.08	0.04	0.00042	0.00031	0.41 (0.25-0.65)	0.05	0.06	0.17	0.32	1.2 (0.90-1.61)	0.39
52	***1740646***	***rs9257453***	***6***	***29,076,909***	***NM_030903, downstream***	***0.13***	***0.07***	***0.00031***	***0.0011***	***0.51 (0.36-0.74)***	***0.09***	***0.08***	***0.38***	***0.29***	***0.87 (0.69-1.10)***	***0.027***
53	1718827	rs10503282	8	4,626,114	CSMD1, intron	0.11	0.06	0.00031	0.0016	0.5 (0.33-0.74)	0.09	0.09	0.98	0.86	1.01 (0.80-1.28)	0.84
54	1678959	rs3211830	7	79,924,369	CD36, intron	0.09	0.04	0.00033	0.0019	0.46 (0.30-0.72)	0.06	0.07	0.34	0.58	1.15 (0.88-1.49)	0.59
55	1672210	rs10509991	10	117,340,550	ATRNL1, intron	0.45	0.52	0.0059	0.00037	1.1 (0.81-1.50)	0.45	0.47	0.30	0.13	1.19 (0.98-1.45)	0.87
56	1668703	rs7582749	2	37,510,988	QPCT, intron	0.04	0.02	0.17	0.00037	0.44 (0.22-0.89)	0.04	0.04	0.91	0.97	0.98 (0.69-1.37)	0.80
57	1643446	rs7263053	20	46,496,933	SULF2, upstream	0.25	0.28	0.22	0.00039	1.45 (1.09-1.92)	0.27	0.29	0.23	0.44	1.09 (0.91-1.31)	0.46
58	***1662241***	***rs10483112***	***22***	***21,051,522***	***BC030984, intron***	***0.04***	***0.01***	***0.00039***	***0.0020***	***0.31 (0.15-0.62)***	***0.03***	***0.02***	***0.025***	***0.024***	***0.6 (0.40-0.92)***	***0.37***
59	1712491	rs10496586	2	122,902,815	TSN, downstream	0.22	0.30	0.00039	0.0018	1.64 (1.23-2.19)	0.27	0.26	0.27	0.52	0.9 (0.76-1.08)	0.32
60	1704979	rs10505673	8	138,447,703	KHDRBS3, downstream	0.24	0.17	0.00071	0.00041	0.7 (0.52-0.93)	0.21	0.21	0.98	0.69	1.03 (0.86-1.23)	0.90
61	1713059	rs1845983	4	76,491,942	DKFZP564O0823, downstream	0.19	0.13	0.00042	0.0018	0.6 (0.44-0.82)	0.16	0.15	0.48	0.31	0.9 (0.74-1.09)	0.23
62	1671422	rs1546210	6	92,633,312	MAP3K7, upstream	0.25	0.17	0.00042	0.0017	0.59 (0.44-0.79)	0.21	0.21	0.86	0.58	0.95 (0.80-1.14)	0.19
63	1697996	rs8005131	14	32,660,856	NPAS3, intron	0.29	0.37	0.00042	0.0031	1.56 (1.17-2.08)	0.32	0.33	0.44	0.35	1.12 (0.94-1.33)	0.57
64	***1731332***	***rs7428001***	***3***	***35,514,961***	***PDCD6IP, downstream***	***0.38***	***0.47***	***0.00043***	***0.0013***	***1.65 (1.22-2.25)***	***0.44***	***0.44***	***0.84***	***0.97***	***0.99 (0.82-1.20)***	***0.012***
65	1740198	rs10508921	10	51,680,714	NM_014114, downstream	0.12	0.07	0.00043	0.0019	0.53 (0.37-0.77)	0.08	0.08	0.66	0.82	0.96 (0.75-1.22)	0.49
66	1748208	rs1559781	4	138,582,822	SLC7A11, downstream	0.03	0.01	0.00044	0.0028	0.19 (0.06-0.55)	0.04	0.05	0.65	0.90	1.07 (0.78-1.47)	0.65
67	1700287	rs4968535	17	56,600,056	BCAS3, intron	0.23	0.21	0.36	0.00045	0.72 (0.54-0.96)	0.24	0.25	0.27	0.17	1.04 (0.87-1.25)	0.60
68	1711405	rs1793483	11	76,653,115	PAK1, upstream	0.31	0.39	0.00046	0.0016	1.61 (1.21-2.15)	0.37	0.38	0.41	0.71	1.06 (0.89-1.27)	0.87
69	1669739	rs10490395	2	56,359,917	KIAA1912, intron	0.05	0.02	0.0039	0.00047	0.35 (0.18-0.67)	0.03	0.03	0.70	0.63	0.91 (0.64-1.30)	0.76
70	***1724612***	***rs1992662***	***5***	***40,429,609***	***PTGER4, upstream***	***0.33***	***0.25***	***0.00047***	***0.0018***	***0.6 (0.45-0.80)***	***0.32***	***0.26***	***7.59E-05***	***0.00017***	***0.76 (0.63-0.91)***	***0.0013***
71	1751402	rs4323713	1	111,535,262	CHI3L2, downstream	0.19	0.26	0.00047	0.0033	1.61 (1.20-2.17)	0.26	0.27	0.60	0.87	1.04 (0.87-1.25)	0.43
72	***1672987***	***rs6947579***	***7***	***125,126,957***	***GRM8, downstream***	***0.26***	***0.34***	***0.00048***	***0.0022***	***1.51 (1.14-2.00)***	***0.29***	***0.33***	***0.0065***	***0.013***	***1.18 (0.99-1.41)***	***1.00***
73	1726836	rs812965	3	59,711,339	FHIT, downstream	0.15	0.22	0.00048	0.0018	1.64 (1.22-2.21)	0.21	0.20	0.30	0.59	0.91 (0.76-1.09)	0.30
74	1664846	rs10494257	1	144,479,385	GJA5, upstream	0.22	0.15	0.00050	0.00080	0.56 (0.42-0.76)	0.21	0.19	0.16	0.35	0.88 (0.73-1.06)	0.84
75	***1703018***	***rs1992660***	***5***	***40,450,824***	***PTGER4, upstream***	***0.40***	***0.31***	***0.00050***	***0.0020***	***0.64 (0.48-0.86)***	***0.38***	***0.31***	***4.53E-05***	***0.00021***	***0.72 (0.60-0.86)***	***0.00050***
76	1650953	rs3922590	10	133,121,612	NM_018461, upstream	0.10	0.16	0.00050	0.0022	1.77 (1.28-2.46)	0.12	0.12	0.55	0.75	1.05 (0.85-1.29)	0.19
77	1691966	rs10498871	6	70,969,556	COL19A1, intron	0.28	0.28	0.94	0.00051	0.78 (0.59-1.04)	0.28	0.29	0.39	0.15	1.01 (0.84-1.20)	0.90
78	1672368	rs3764007	12	20,905,430	SLCO1B3, intron	0.16	0.10	0.00051	0.0017	0.54 (0.39-0.76)	0.16	0.16	0.73	0.91	0.98 (0.80-1.19)	0.34
79	***1689671***	***rs1553575***	***5***	***40,538,689***	***PTGER4, upstream***	***0.38***	***0.29***	***0.00051***	***0.0021***	***0.65 (0.49-0.87)***	***0.35***	***0.28***	***1.68E-06***	***6.37E-06***	***0.65 (0.55-0.78)***	***0.051***
80	1703646	rs8000906	13	68,661,229	PCDH9, upstream	0.09	0.05	0.00053	0.0019	0.5 (0.33-0.78)	0.09	0.09	0.88	0.99	1.02 (0.80-1.29)	0.83
81	1715786	rs1463446	12	101,488,216	PAH, downstream	0.35	0.44	0.00053	0.0029	1.55 (1.16-2.07)	0.39	0.40	0.32	0.60	1.08 (0.90-1.29)	0.74
82	1686118	rs10502861	18	41,054,146	SLC14A2, upstream	0.24	0.32	0.00053	0.0020	1.5 (1.13-1.99)	0.27	0.29	0.32	0.22	1.03 (0.87-1.23)	0.86
83	***1663457***	***rs1793004***	***11***	***20,655,505***	***NELL1, intron***	***0.27***	***0.20***	***0.00053***	***0.0020***	***0.59 (0.44-0.80)***	***0.28***	***0.24***	***0.025***	***0.052***	***0.85 (0.71-1.00)***	***0.045***
84	***1678461***	***rs10487428***	***7***	***125,164,048***	***GRM8, downstream***	***0.24***	***0.32***	***0.00054***	***0.0018***	***1.65 (1.24-2.19)***	***0.27***	***0.30***	***0.028***	***0.10***	***1.19 (1.00-1.42)***	***0.54***
85	1705772	rs8011773	14	96,927,485	VRK1, downstream	0.18	0.25	0.00054	0.0041	1.6 (1.19-2.16)	0.20	0.23	0.057	0.084	1.13 (0.94-1.36)	0.37
86	***1663676***	***rs10484545***	***6***	***29,342,489***	***OR5V1, downstream***	***0.11***	***0.06***	***0.00054***	***0.0017***	***0.5 (0.34-0.73)***	***0.07***	***0.10***	***0.00030***	***2.84E-07***	***1.28 (1.00-1.63)***	***0.16***
87	1672449	rs1493517	4	134,829,467	SLC7A11, downstream	0.38	0.29	0.00054	0.0023	0.64 (0.48-0.85)	0.37	0.35	0.23	0.48	0.91 (0.76-1.09)	0.11
88	1755381	rs10517634	4	157,502,957	CTSO, upstream	0.36	0.45	0.00055	0.00066	1.39 (1.03-1.87)	0.43	0.42	0.44	0.72	0.95 (0.79-1.15)	0.21
89	1697863	rs9307696	4	134,844,616	SLC7A11, downstream	0.38	0.29	0.00057	0.0024	0.64 (0.48-0.85)	0.37	0.35	0.15	0.35	0.89 (0.75-1.07)	0.095
90	1670687	rs6673693	1	206,111,815	AY320401, downstream	0.29	0.37	0.00060	0.0034	1.52 (1.15-2.02)	0.30	0.32	0.39	0.42	1.12 (0.93-1.33)	0.73
91	1686876	rs1488927	7	153,840,317	BC035912, downstream	0.40	0.48	0.0025	0.00061	1.2 (0.89-1.62)	0.45	0.45	0.86	0.68	1.03 (0.85-1.25)	0.50
92	1757706	rs1536299	13	23,216,963	MIPEP, intron	0.29	0.21	0.00061	0.0035	0.62 (0.47-0.83)	0.23	0.26	0.056	0.13	1.2 (1.00-1.43)	0.15
93	1665309	rs289386	15	83,382,393	PDE8A, intron	0.41	0.49	0.00062	0.0026	1.63 (1.20-2.23)	0.43	0.43	0.87	0.61	0.94 (0.78-1.13)	0.91
94	***1662678***	***rs723510***	***8***	***78,662,620***	***PXMP3, upstream***	***0.39***	***0.31***	***0.00062***	***0.00069***	***0.58 (0.43-0.76)***	***0.36***	***0.32***	***0.037***	***0.093***	***0.82 (0.69-0.98)***	***0.069***
95	1724142	rs10514013	18	67,305,710	NM_178506, upstream	0.09	0.05	0.00078	0.00063	0.45 (0.29-0.70)	0.06	0.06	0.75	0.72	1.06 (0.81-1.39)	0.82
96	1743512	rs1413829	9	27,191,968	TEK, intron	0.28	0.20	0.00064	0.0032	0.66 (0.49-0.87)	0.28	0.26	0.27	0.45	0.89 (0.75-1.07)	0.43
97	***1735637***	***rs7795363***	***7***	***31,560,089***	***PDE1C, downstream***	***0.17***	***0.11***	***0.00066***	***0.0026***	***0.57 (0.41-0.79)***	***0.16***	***0.15***	***0.34***	***0.027***	***0.99 (0.81-1.20)***	***0.71***
98	1728709	rs951308	10	76,287,485	MYST4, intron	0.10	0.15	0.00067	0.0015	1.85 (1.32-2.60)	0.09	0.10	0.62	0.56	1.09 (0.86-1.36)	0.85
99	1664382	rs10486725	7	41,645,949	GLI3, downstream	0.08	0.13	0.00068	0.0023	1.77 (1.23-2.55)	0.10	0.10	0.72	0.26	0.92 (0.74-1.15)	0.93
100	***1694998***	***rs396462***	***8***	***17,714,476***	***FGL1, downstream***	***0.24***	***0.17***	***0.00069***	***0.0018***	***0.68 (0.50-0.91)***	***0.21***	***0.22***	***0.46***	***0.46***	***1.03 (0.86-1.24)***	***0.030***
101	1643555	rs6122682	20	46,497,290	SULF2, upstream	0.24	0.27	0.29	0.00070	1.41 (1.06-1.87)	0.26	0.27	0.69	0.66	1 (0.84-1.20)	0.80
102	***1669289***	***rs10489924***	***1***	***99,170,995***	***AK095544, upstream***	***0.13***	***0.08***	***0.00070***	***0.0024***	***0.56 (0.39-0.80)***	***0.10***	***0.10***	***0.65***	***0.57***	***1.03 (0.82-1.29)***	***0.026***
103	1734534	rs1859346	5	136,475,473	SPOCK, intron	0.33	0.26	0.00071	0.0018	0.61 (0.46-0.81)	0.31	0.30	0.55	0.69	0.93 (0.78-1.11)	0.25
104	***1658904***	***rs9283536***	***2***	***223,756,011***	***SCG2, downstream***	***0.23***	***0.30***	***0.00071***	***0.0016***	***1.5 (1.13-2.00)***	***0.26***	***0.28***	***0.24***	***0.42***	***1.08 (0.90-1.28)***	***0.0052***
105	***1653909***	***rs7725523***	***5***	***40,407,980***	***PTGER4, upstream***	***0.27***	***0.19***	***0.00073***	***0.0047***	***0.63 (0.47-0.85)***	***0.25***	***0.23***	***0.14***	***0.32***	***0.87 (0.73-1.04)***	***0.013***
106	***1721150***	***rs6461599***	***7***	***21,573,649***	***DNAH11, intron***	***0.17***	***0.11***	***0.00074***	***0.0040***	***0.59 (0.43-0.82)***	***0.14***	***0.14***	***0.90***	***0.67***	***0.99 (0.81-1.21)***	***0.018***
107	1710316	rs1419790	7	34,548,784	BMPER, downstream	0.15	0.17	0.54	0.00074	1.3 (0.95-1.77)	0.16	0.17	0.71	0.49	1 (0.82-1.21)	0.25
108	1725734	rs10504252	8	59,380,280	LOC137886, upstream	0.21	0.14	0.00074	0.0029	0.58 (0.43-0.80)	0.20	0.19	0.85	0.97	0.98 (0.81-1.18)	0.072
109	1707295	rs2832238	21	29,471,232	C21orf7, downstream	0.25	0.18	0.00076	0.0030	0.58 (0.43-0.77)	0.23	0.23	0.83	0.63	1.05 (0.88-1.26)	1.00
110	1662590	rs549700	11	88,277,830	GRM5, intron	0.04	0.01	0.00088	0.00076	0.28 (0.12-0.61)	0.02	0.02	0.82	0.97	1.05 (0.67-1.65)	0.73
111	1679422	rs10497212	2	160,790,207	ITGB6, intron	0.12	0.18	0.00077	0.0036	1.68 (1.22-2.29)	0.13	0.14	0.41	0.46	1.06 (0.86-1.30)	0.64

112	1758931	rs4707614	6	91,138,857	MAP3K7, downstream	0.42	0.39	0.16	0.00078	0.64 (0.48-0.86)	0.41	0.41	0.96	1.00	1 (0.83-1.20)	0.55
113	1649870	rs10485515	20	14,797,853	BC036403, intron	0.17	0.24	0.00078	0.0016	1.46 (1.09-1.96)	0.21	0.22	0.33	0.53	1.07 (0.89-1.28)	0.85
114	1701880	rs10494248	1	144,333,076	BCL9, downstream	0.03	0.01	0.00087	0.00079	0.19 (0.06-0.56)	0.01	0.01	0.67	0.65	0.92 (0.54-1.58)	0.34
115	***1660459***	***rs10501292***	***11***	***42,952,963***	***API5, upstream***	***0.15***	***0.10***	***0.0098***	***0.00080***	***0.58 (0.41-0.81)***	***0.12***	***0.12***	***0.67***	***0.52***	***0.98 (0.80-1.21)***	***0.015***
116	***1719121***	***rs951199***	***11***	***20,657,351***	***NELL1, intron***	***0.28***	***0.21***	***0.00080***	***0.0027***	***0.62 (0.46-0.82)***	***0.27***	***0.24***	***0.029***	***0.064***	***0.86 (0.72-1.02)***	***0.27***
117	***1749234***	***rs10520704***	***15***	***90,521,604***	***SIAT8B, upstream***	***0.35***	***0.44***	***0.00080***	***0.0033***	***1.47 (1.10-1.97)***	***0.41***	***0.38***	***0.020***	***0.044***	***0.86 (0.71-1.03)***	***0.077***
118	1682582	rs569626	22	26,071,824	CRYBA4, downstream	0.48	0.41	0.0020	0.00080	0.55 (0.41-0.75)	0.46	0.44	0.13	0.32	0.87 (0.72-1.05)	0.33
119	1692328	rs9323605	14	24,686,376	NOVA1, downstream	0.12	0.07	0.00081	0.0034	0.53 (0.36-0.77)	0.12	0.11	0.21	0.38	0.86 (0.70-1.07)	0.62
120	***1678160***	***rs10508621***	***10***	***20,686,445***	***NEBL, downstream***	***0.12***	***0.07***	***0.00082***	***0.0047***	***0.55 (0.38-0.80)***	***0.10***	***0.10***	***0.91***	***0.99***	***1.01 (0.81-1.26)***	***0.029***
121	1713936	rs10508494	10	15,861,784	C10orf97, intron	0.28	0.21	0.0037	0.00082	0.59 (0.44-0.79)	0.24	0.25	0.28	0.33	1.06 (0.89-1.27)	0.70
122	***1710126***	***rs3118223***	***1***	***49,175,821***	***FLJ11588, intron***	***0.37***	***0.46***	***0.00082***	***0.0033***	***1.4 (1.04-1.89)***	***0.40***	***0.37***	***0.026***	***0.084***	***0.84 (0.70-1.00)***	***0.47***
123	1647863	rs10500069	7	118,109,407	KCND2, upstream	0.24	0.17	0.00083	0.0029	0.65 (0.48-0.87)	0.21	0.21	0.54	0.81	0.94 (0.79-1.13)	0.90
124	1710468	rs2134066	12	42,000,804	ADAMTS20, downstream	0.48	0.40	0.00083	0.0029	0.68 (0.50-0.92)	0.43	0.45	0.24	0.30	1.16 (0.96-1.41)	0.074
125	***1713324***	***rs4743484***	***9***	***101,559,035***	***PPP3R2, intron***	***0.26***	***0.19***	***0.00083***	***0.0025***	***0.61 (0.46-0.81)***	***0.25***	***0.21***	***0.0016***	***0.0069***	***0.78 (0.65-0.93)***	***0.90***
126	***1753829***	***rs9304017***	***18***	***8,617,595***	***KIAA0802, upstream***	***0.13***	***0.14***	***0.36***	***0.00084***	***0.93 (0.66-1.29)***	***0.14***	***0.16***	***0.082***	***0.22***	***1.17 (0.96-1.42)***	***0.042***
127	1656796	rs3096085	5	146,026,611	PPP2R2B, intron	0.02	0.05	0.0010	0.00085	2.75 (1.49-5.08)	0.04	0.03	0.44	0.75	0.88 (0.62-1.24)	0.64
128	1665215	rs10519449	15	21,601,298	NDN, upstream	0.10	0.12	0.22	0.00086	1.01 (0.71-1.45)	0.12	0.13	0.79	0.64	1 (0.81-1.23)	0.38
129	***1693727***	***rs10255000***	***7***	***14,814,769***	***DGKB, upstream***	***0.16***	***0.10***	***0.00086***	***0.0038***	***0.57 (0.41-0.80)***	***0.15***	***0.13***	***0.025***	***0.088***	***0.8 (0.66-0.98)***	***0.32***
130	1748043	rs2852095	18	45,834,049	MYO5B, intron	0.38	0.30	0.00090	0.0057	0.67 (0.50-0.89)	0.33	0.33	0.72	0.76	1 (0.83-1.19)	0.82
131	***1668856***	***rs10500037***	***7***	***113,301,738***	***PPP1R3A, upstream***	***0.29***	***0.37***	***0.00091***	***0.0046***	***1.46 (1.10-1.94)***	***0.34***	***0.30***	***0.013***	***0.044***	***0.81 (0.68-0.97)***	***0.051***
132	1693104	rs1528039	7	110,036,723	IMMP2L, intron	0.25	0.18	0.00091	0.0032	0.61 (0.46-0.82)	0.21	0.22	0.75	0.12	0.96 (0.80-1.15)	0.30
133	1729588	rs770918	1	98,640,958	SNX7, upstream	0.16	0.10	0.00091	0.0014	0.61 (0.44-0.85)	0.14	0.13	0.74	0.54	0.94 (0.76-1.15)	0.44
134	1694761	rs1994893	16	17,820,087	LOC339047, downstream	0.17	0.12	0.0075	0.00092	0.58 (0.42-0.80)	0.14	0.14	0.62	0.23	1 (0.82-1.22)	0.33
135	***1705295***	***rs1513287***	***3***	***114,754,898***	***FLJ20174, intron***	***0.44***	***0.35***	***0.00093***	***0.0052***	***0.65 (0.48-0.86)***	***0.41***	***0.37***	***0.017***	***0.023***	***0.77 (0.65-0.93)***	***1.00***
136	1688914	rs3847621	11	35,321,011	SLC1A2, intron	0.38	0.37	0.77	0.00093	0.74 (0.55-0.99)	0.37	0.37	0.60	0.75	1.02 (0.85-1.22)	0.82
137	1689102	rs2837758	21	40,940,879	DSCAM, intron	0.42	0.37	0.061	0.00095	0.99 (0.74-1.33)	0.41	0.40	0.61	0.83	0.97 (0.81-1.17)	0.55
138	1661156	rs10506296	12	49,374,124	KIAA1463, upstream	0.04	0.01	0.0011	0.00096	0.28 (0.13-0.62)	0.03	0.02	0.10	0.18	0.73 (0.47-1.11)	0.85
139	1691716	rs10503536	8	15,057,605	TUSC3, upstream	0.17	0.23	0.00096	0.0036	1.56 (1.16-2.09)	0.20	0.21	0.47	0.72	1.05 (0.88-1.27)	0.90
140	1657660	rs10492248	12	44,329,880	TMEM16F, downstream	0.30	0.23	0.00096	0.0042	0.66 (0.49-0.87)	0.24	0.26	0.11	0.22	1.17 (0.98-1.40)	0.23
141	1727943	rs10520778	15	93,728,732	FLJ11175, downstream	0.41	0.34	0.0034	0.00097	0.59 (0.44-0.78)	0.37	0.36	0.63	0.18	0.89 (0.74-1.07)	0.34
142	1655695	rs2241330	3	8,832,963	OXTR, upstream	0.07	0.03	0.00100	0.0041	0.45 (0.27-0.74)	0.08	0.10	0.12	0.17	1.23 (0.97-1.55)	0.79
143	1667263	rs7200548	16	5,830,572	A2BP1, upstream	0.18	0.25	0.0010	0.0035	1.63 (1.22-2.17)	0.19	0.18	0.78	0.96	0.97 (0.81-1.17)	0.30
144	1671948	rs10507786	13	80,217,127	BC016673, downstream	0.24	0.17	0.0010	0.0023	0.67 (0.50-0.90)	0.22	0.22	0.69	0.92	0.97 (0.81-1.16)	0.41
145	1748626	rs10500715	11	9,929,638	CMT4B2, intron	0.40	0.48	0.0011	0.0017	1.31 (0.97-1.76)	0.45	0.44	0.59	0.17	1.05 (0.87-1.27)	0.16
146	1675192	rs10512301	9	102,401,716	CYLC2, upstream	0.04	0.08	0.0011	0.0035	2.06 (1.33-3.19)	0.06	0.07	0.46	0.60	1.12 (0.86-1.47)	1.00
147	***1661269***	***rs9304344***	***18***	***43,012,663***	***AY134745, downstream***	***0.40***	***0.48***	***0.0011***	***0.0043***	***1.46 (1.08-1.98)***	***0.45***	***0.45***	***0.98***	***0.047***	***0.88 (0.73-1.07)***	***0.74***
148	1758667	rs3886870	2	45,954,129	PRKCE, intron	0.22	0.29	0.0011	0.0051	1.51 (1.14-2.00)	0.25	0.25	0.68	0.35	1.01 (0.84-1.22)	0.67
149	1699371	rs1446240	15	57,083,783	RNF111, intron	0.18	0.12	0.0011	0.0042	0.58 (0.42-0.81)	0.19	0.17	0.12	0.31	0.87 (0.72-1.05)	0.51
150	1746102	rs9283944	8	107,403,285	ZFPM2, downstream	0.25	0.20	0.052	0.0011	0.64 (0.48-0.86)	0.23	0.25	0.097	0.17	1.11 (0.93-1.33)	0.096
151	***1751270***	***rs1260126***	***7***	***145,261,967***	***CNTNAP2, intron***	***0.24***	***0.17***	***0.0012***	***0.0056***	***0.64 (0.48-0.86)***	***0.23***	***0.19***	***0.011***	***0.015***	***0.77 (0.64-0.92)***	***0.17***
152	***1715515***	***rs2123647***	***8***	***96,000,958***	***CCNE2, upstream***	***0.34***	***0.28***	***0.012***	***0.0012***	***0.61 (0.46-0.81)***	***0.35***	***0.33***	***0.13***	***0.22***	***0.85 (0.71-1.02)***	***0.0088***
153	1667987	rs6501291	17	64,732,049	ABCA10, intron	0.38	0.30	0.0012	0.0021	0.6 (0.45-0.80)	0.38	0.37	0.45	0.62	0.92 (0.77-1.10)	0.42
154	1660643	rs10483431	14	32,751,215	NPAS3, intron	0.16	0.13	0.11	0.0012	0.66 (0.48-0.91)	0.14	0.14	0.90	0.95	1 (0.82-1.22)	0.33
155	1695412	rs2173019	5	167,547,549	ODZ2, intron	0.22	0.16	0.0012	0.0012	0.68 (0.50-0.91)	0.16	0.16	0.85	0.62	1.05 (0.86-1.27)	0.66
156	***1674500***	***rs778999***	***5***	***152,887,880***	***GRIA1, intron***	***0.32***	***0.24***	***0.0012***	***0.0032***	***0.62 (0.46-0.82)***	***0.34***	***0.31***	***0.016***	***0.037***	***0.84 (0.70-1.00)***	***0.14***
157	1645109	rs9302576	16	64,469,579	CDH5, upstream	0.05	0.02	0.0077	0.0012	0.4 (0.22-0.74)	0.05	0.04	0.30	0.48	0.86 (0.63-1.17)	0.89
158	1680152	rs9314133	5	94,552,156	FLJ22344, upstream	0.22	0.29	0.0012	0.0014	1.42 (1.07-1.89)	0.26	0.24	0.11	0.11	0.92 (0.77-1.10)	0.56
159	***1678082***	***rs10521209***	***16***	***49,313,210***	***CARD15, intron***	***0.41***	***0.33***	***0.0013***	***0.0031***	***0.7 (0.52-0.93)***	***0.41***	***0.35***	***8.65E-05***	***8.68E-05***	***0.67 (0.56-0.80)***	***0.0083***
160	1727370	rs6821328	4	54,058,427	SCFD2, intron	0.45	0.46	0.59	0.0013	0.8 (0.59-1.08)	0.47	0.49	0.28	0.46	1.13 (0.93-1.38)	0.27
161	***1745616***	***rs1161308***	***5***	***99,248,531***	***CHD1, upstream***	***0.30***	***0.31***	***0.65***	***0.0013***	***0.84 (0.63-1.12)***	***0.32***	***0.29***	***0.13***	***0.30***	***0.89 (0.75-1.07)***	***0.011***
162	***1651987***	***rs10507353***	***13***	***25,700,935***	***CDK8, upstream***	***0.45***	***0.54***	***0.0013***	***0.0064***	***1.57 (1.13-2.17)***	***0.50***	***0.49***	***0.75***	***0.92***	***0.99 (0.81-1.21)***	***0.019***
163	***1744306***	***rs2631372***	***5***	***131,731,477***	***SLC22A4, downstream***	***0.31***	***0.28***	***0.26***	***0.0013***	***1.06 (0.80-1.41)***	***0.33***	***0.29***	***0.0080***	***0.022***	***0.78 (0.65-0.93)***	***0.030***
164	1643625	rs2699750	7	118,082,982	KCND2, upstream	0.24	0.18	0.0013	0.0021	0.68 (0.51-0.91)	0.22	0.21	0.67	0.91	0.97 (0.81-1.16)	0.70
165	1738328	rs4894919	3	106,541,038	ALCAM, upstream	0.28	0.35	0.0018	0.0013	1.33 (1.01-1.76)	0.31	0.30	0.38	0.58	0.95 (0.80-1.13)	0.73
166	1673656	rs10505696	8	139,299,886	COL22A1, downstream	0.38	0.30	0.0013	0.0024	0.6 (0.45-0.80)	0.32	0.32	0.75	0.38	0.92 (0.77-1.10)	0.73
167	1713906	rs1435385	4	37,051,072	KIAA1239, upstream	0.08	0.09	0.57	0.0013	0.86 (0.57-1.29)	0.08	0.07	0.40	0.44	0.88 (0.68-1.13)	0.84
168	1690890	rs10520307	2	82,082,432	SUCLG1, downstream	0.11	0.06	0.0014	0.0014	0.5 (0.33-0.74)	0.08	0.08	0.65	0.34	0.92 (0.72-1.17)	0.68
169	***1720469***	***rs7607642***	***2***	***182,817,682***	***LOC151242, intron***	***0.32***	***0.39***	***0.0022***	***0.0014***	***1.31 (0.98-1.75)***	***0.36***	***0.37***	***0.37***	***0.64***	***1.07 (0.89-1.28)***	***0.0052***
170	1680241	rs1930551	9	102,419,896	CYLC2, upstream	0.04	0.08	0.0020	0.0014	2.06 (1.31-3.22)	0.06	0.07	0.36	0.49	1.15 (0.88-1.51)	0.50
171	***1699949***	***rs7868736***	***9***	***113,607,737***	***NM_152575, upstream***	***0.24***	***0.31***	***0.0014***	***0.0016***	***1.39 (1.04-1.84)***	***0.25***	***0.29***	***0.0025***	***0.0065***	***1.24 (1.04-1.48)***	***0.64***
172	1736493	rs1891281	6	14,704,630	CD83, downstream	0.19	0.14	0.012	0.0014	0.6 (0.44-0.82)	0.16	0.16	0.76	0.86	1.01 (0.84-1.23)	0.61
173	***1688588***	***rs1823634***	***11***	***4,397,414***	***SSA1, upstream***	***0.31***	***0.33***	***0.39***	***0.0014***	***0.89 (0.67-1.18)***	***0.31***	***0.29***	***0.23***	***0.47***	***0.91 (0.76-1.08)***	***0.020***
174	1665706	rs1073298	7	28,138,715	CREB5, upstream	0.15	0.13	0.20	0.0014	0.94 (0.68-1.29)	0.16	0.14	0.21	0.43	0.88 (0.72-1.07)	0.51
175	1702872	rs10487909	7	15,879,068	MEOX2, upstream	0.01	0.03	0.0017	0.0015	3.66 (1.56-8.60)	0.03	0.02	0.069	0.14	0.69 (0.44-1.07)	0.59
176	1714833	rs2823651	21	16,506,279	AF486622, intron	0.31	0.39	0.0015	0.0067	1.56 (1.18-2.07)	0.34	0.37	0.062	0.12	1.2 (1.01-1.44)	0.65
177	1665892	rs10521132	16	25,628,337	HS3ST4, intron	0.23	0.19	0.036	0.0015	0.64 (0.47-0.86)	0.24	0.24	0.71	0.62	1 (0.84-1.20)	0.34
178	***1648042***	***rs2964551***	***5***	***114,739,639***	***FEM1C, downstream***	***0.03***	***0.01***	***0.0017***	***0.0015***	***0.27 (0.12-0.64)***	***0.02***	***0.02***	***0.97***	***0.64***	***1.01 (0.67-1.52)***	***0.016***
179	1722370	rs2385635	2	220,597,651	EPHA4, downstream	0.23	0.19	0.058	0.0015	0.65 (0.48-0.88)	0.24	0.22	0.26	0.36	0.88 (0.74-1.06)	0.57
180	1718706	rs746413	20	40,143,404	PTPRT, intron	0.41	0.45	0.13	0.0016	1.61 (1.18-2.18)	0.41	0.40	0.46	0.71	0.96 (0.80-1.15)	1.00
181	1668809	rs10516960	4	96,516,855	UNC5C, intron	0.42	0.50	0.0016	0.0026	1.37 (1.00-1.86)	0.49	0.48	0.60	0.58	1.01 (0.82-1.23)	0.40
182	1646814	rs1160521	1	190,469,936	B3GALT2, upstream	0.11	0.06	0.0032	0.0016	0.51 (0.34-0.76)	0.11	0.10	0.65	0.57	0.98 (0.79-1.22)	0.21
183	1746058	rs1953915	14	48,230,239	RPS29, downstream	0.42	0.50	0.0016	0.0024	1.73 (1.26-2.36)	0.45	0.47	0.25	0.53	1.1 (0.91-1.33)	0.78
184	1673470	rs2864038	10	106,879,028	SORCS3, intron	0.02	0.00	0.0018	0.0017	0.17 (0.05-0.59)	0.02	0.01	0.63	0.63	0.88 (0.53-1.47)	0.10
185	1758121	rs1420626	12	14,782,776	GUCY2C, upstream	0.25	0.18	0.0030	0.0017	0.59 (0.44-0.80)	0.24	0.24	0.94	1.00	0.99 (0.83-1.18)	0.71
186	1727427	rs491552	6	151,403,169	FTHFSDC1, intron	0.42	0.45	0.26	0.0017	0.88 (0.65-1.19)	0.45	0.45	0.68	0.42	0.9 (0.75-1.09)	0.82
187	1755134	rs10505614	8	135,071,436	ZNF406, downstream	0.33	0.27	0.018	0.0017	0.62 (0.47-0.83)	0.29	0.30	0.56	0.84	1.05 (0.88-1.25)	0.81
188	***1676184***	***rs10515690***	***5***	***152,909,131***	***GRIA1, intron***	***0.43***	***0.36***	***0.0060***	***0.0017***	***0.59 (0.44-0.79)***	***0.43***	***0.40***	***0.043***	***0.054***	***0.9 (0.75-1.09)***	***0.25***
189	1643487	rs6726390	2	67,988,748	C1D, downstream	0.14	0.09	0.0017	0.0027	0.63 (0.45-0.88)	0.11	0.12	0.72	0.91	1.05 (0.84-1.29)	0.17
190	***1730891***	***rs1695183***	***3***	***23,497,789***	***UBE2E2, intron***	***0.22***	***0.16***	***0.0028***	***0.0018***	***0.59 (0.43-0.80)***	***0.22***	***0.19***	***0.10***	***0.22***	***0.85 (0.71-1.02)***	***0.023***
191	***1688424***	***rs272867***	***5***	***131,708,956***	***SLC22A4, downstream***	***0.40***	***0.37***	***0.15***	***0.0018***	***1.05 (0.78-1.40)***	***0.43***	***0.38***	***0.0034***	***0.0085***	***0.74 (0.62-0.90)***	***0.26***
192	***1755639***	***rs830772***	***8***	***76,515,133***	***HNF4G, intron***	***0.15***	***0.19***	***0.012***	***0.0019***	***1.26 (0.93-1.71)***	***0.15***	***0.17***	***0.23***	***0.0099***	***1.21 (1.00-1.47)***	***0.14***
193	1685410	rs10486718	7	41,432,657	INHBA, downstream	0.02	0.01	0.0020	0.0019	0.21 (0.07-0.62)	0.02	0.02	0.12	0.23	1.41 (0.89-2.24)	0.47
194	***1755609***	***rs747357***	***4***	***9,829,147***	***KIAA1729, downstream***	***0.43***	***0.51***	***0.0019***	***0.011***	***1.5 (1.10-2.04)***	***0.43***	***0.46***	***0.085***	***0.010***	***1.32 (1.09-1.60)***	***0.92***
195	1736719	rs2025052	14	29,844,295	PRKCM, upstream	0.12	0.13	0.78	0.0019	0.88 (0.63-1.23)	0.13	0.14	0.50	0.44	1.04 (0.85-1.27)	0.87
196	1662317	rs6112307	20	19,237,755	SLC24A3, intron	0.36	0.35	0.57	0.0020	0.73 (0.55-0.98)	0.35	0.35	0.93	0.19	1.07 (0.90-1.28)	0.14
197	***1680706***	***rs1591783***	***1***	***154,019,189***	***ETV3, upstream***	***0.47***	***0.39***	***0.0028***	***0.0021***	***0.58 (0.42-0.78)***	***0.47***	***0.43***	***0.024***	***0.074***	***0.85 (0.70-1.03)***	***0.38***
198	1657740	rs2023661	16	26,362,243	HS3ST4, downstream	0.44	0.36	0.0028	0.0021	0.59 (0.44-0.79)	0.42	0.42	0.84	0.58	0.93 (0.77-1.13)	0.87
199	1729462	rs7732995	5	106,614,697	EFNA5, downstream	0.24	0.31	0.0021	0.0077	1.48 (1.12-1.97)	0.29	0.29	0.83	0.57	0.95 (0.79-1.13)	0.23
200	1671138	rs3790889	1	82,124,621	LPHN2, intron	0.39	0.37	0.43	0.0021	0.72 (0.54-0.95)	0.40	0.40	0.86	0.99	1.01 (0.84-1.22)	0.74
