# Supplementary material for: Systematic Association Mapping Identifies NELL1 as a Novel IBD Disease Gene
Source: PLoS One. 2007 Aug 8;2(8):e691. doi: 10.1371/journal.pone.0000691 (PMC1933598; doi:10.1371/journal.pone.0000691)
Supplement: Table S3 — Fine mapping of the CD association signal at the NELL1 locus in replication panels B and D. The p-values of the allele-based (pCCA) and genotype-based (pCCG) association analyses of the tagging SNPs are shown, pTDT is the p-value for the transmission disequilibrium test (TDT). Lead SNPs from the initial screening (see Table S2) are highlighted by grey shading, nonsynonymous SNPs in red color. Polymorphisms that are significant in either the TDT or the case-control analyses, are highlighted in bold italics and those significant in both are highlighted in blue color. Pairwise LD is listed using the metric r2 as calculated with Haploview [1] and minor allele frequencies (MAF) are listed for control individuals. Nucleotide positions refer to NCBI build 35. (0.14 MB PDF) [file pone.0000691.s011.pdf]

Supplementary Table 3: Fine mapping of the CD association signal at the *NELL1* locus in replication panels B and D. The p-values of the allele-based (p_{CCA}) and genotype-based (p_{CCG}) association analyses of the tagging SNPs are shown, p_{TDT} is the p-value for the transmission disequilibrium test (TDT). Lead SNPs from the initial screening (see Table S2) are highlighted by grey shading, nonsynonymous SNPs in red color. Polymorphisms that are significant in either the TDT or the case-control analyses, are highlighted in bold italics and those significant in both are highlighted in blue color. Pairwise LD is listed using the metric r^2 as calculated with Haploview [1] and minor allele frequencies (MAF) are listed for control individuals. Nucleotide positions refer to NCBI build 35.

		Panel B										Panel D				
dbSNP ID	Position	#	Distance [kb]	LD [r^2]	MAF_{co}	MAF_{ca}	p_{CCA}	p_{CCG}	OR (95% CI)	T:U	p_{TDT}	#	Distance [kb]	LD [r^2]	T:U	p_{TDT}
rs3740872	20,605,132	1	-	0.022	0.37	0.38	0.42	0.55	1.1 (0.92-1.32)	166:163	0.87	1	-	0.114	189:162	0.15
rs2000959	20,615,255											2	10.1	0.435	160:143	0.33
rs10766710	20,624,679											3	9.4	0.032	195:156	0.037
rs7122910	20,636,147	2	31.0	0.000	0.30	0.32	0.32	0.60	1.08 (0.90-1.28)	154:147	0.69	4	11.5	0.000	145:138	0.68
rs1792969	20,646,062	3	9.9	0.001	0.11	0.13	0.11	0.048	1.12 (0.90-1.38)	73:71	0.87	5	9.9	0.000	79:66	0.28
NELL1_01	20,647,024	4	1.0	0.007	0.01		0.57	0.56			0.034	6	1.0	0.012	3:2	0.65
rs1793005	20,655,334	5	8.3	0.978	0.27	0.24	0.031	0.063	0.86 (0.72-1.03)	137:126	0.50	7	8.3	0.994	156:117	0.018
rs1793004	20,655,505	6	0.2	0.981	0.28	0.24	0.025	0.056	0.85 (0.71-1.00)	135:104	0.045	8	0.2	0.966	140:107	0.036
rs1793003	20,655,970	7	0.5	0.983	0.27	0.24	0.053	0.096	0.88 (0.74-1.05)	139:128	0.50	9	0.5	0.953	157:119	0.022
rs951199	20,657,351	8	1.4	0.083	0.27	0.24	0.029	0.064	0.86 (0.72-1.03)	141:125	0.27	10	1.4	0.089	151:117	0.038
rs870194	20,663,881	9	6.5	0.509	0.21	0.19	0.28	0.25	0.87 (0.73-1.05)	122:93	0.048	11	6.5	0.505	116:102	0.34
rs1607616	20,674,469	10	10.6	0.521	0.35	0.31	0.0046	0.010	0.76 (0.64-0.91)	191:132	0.0010	12	10.6	0.515	152:140	0.48
rs1792983	20,674,751	11	0.3	0.038	0.21	0.19	0.24	0.23	0.87 (0.72-1.04)	136:99	0.016	13	0.3	0.042	117:102	0.31
rs11025705	20,688,454	12	13.7	0.024	0.14	0.14	0.95	0.99	1.01 (0.83-1.23)	87:71	0.20	14	13.7	0.029	87:75	0.35
rs7952174	20,695,492	13	7.0	0.004	0.15	0.14	0.30	0.54	0.89 (0.73-1.09)	90:82	0.54	15	7.0	0.003	98:71	0.038
rs908940	20,708,141	14	12.6	0.004	0.03	0.02	0.44	0.15	0.81 (0.54-1.22)	22:15	0.25	16	12.6	0.003	14:11	0.55
rs7116986	20,711,993	15	3.9	0.030	0.14	0.14	0.93	0.99	0.99 (0.81-1.21)	89:77	0.35	17	3.9	0.031	92:68	0.058
rs1996623	20,715,992	16	4.0	0.323	0.15	0.15	0.65	0.74	1.02 (0.84-1.25)	87:73	0.27	18	4.0	0.338	95:83	0.37
rs1519727	20,721,594	17	5.6	0.390	0.35	0.35	0.83	0.49	0.94 (0.78-1.12)	151:145	0.73	19	5.6	0.322	162:146	0.36
rs1554368	20,732,334	18	10.7	0.012	0.18	0.17	0.82	0.45	0.95 (0.78-1.14)	114:92	0.13	20	10.7	0.015	102:77	0.062
rs17232778	20,735,857	19	3.5	0.001	0.08	0.07	0.62	0.82	0.95 (0.74-1.22)	48:38	0.28	21	3.5	0.003	47:39	0.39
rs1429796	20,739,579	20	3.7	0.005	0.02	0.01	0.0068	0.0059	0.49 (0.30-0.80)	12:8	0.37	22	3.7	0.017	24:17	0.27
rs4922623	20,741,119	21	1.5	0.363	0.31	0.31	0.97	0.98	1 (0.84-1.19)	165:133	0.064	23	1.5	0.496	144:137	0.68
rs327036	20,742,093											24	1.0	0.411	118:99	0.20
rs327025	20,746,233	22	5.1	0.121	0.15	0.14	0.62	0.40	0.99 (0.81-1.20)	107:77	0.027					
rs16906777	20,748,858	23	2.6	0.025	0.34	0.33	0.42	0.73	0.94 (0.79-1.12)	165:142	0.19	25	6.8	0.034	141:141	1.00
rs327028	20,748,995	24	0.1	0.237	0.08	0.08	0.78	0.84	0.98 (0.77-1.25)	52:44	0.41	26	0.1	0.255	50:47	0.76
rs1949523	20,750,221	25	1.2	0.929	0.27	0.25	0.100	0.075	0.83 (0.69-0.98)	130:125	0.75	27	1.2	0.930	149:110	0.015
rs8176785	20,761,862	26	11.6	0.994	0.27	0.25	0.12	0.039	0.82 (0.69-0.98)	140:126	0.39	28	11.6	1.000	136:109	0.085
rs2280363	20,761,911	27	0.0	0.353	0.27	0.25	0.12	0.075	0.83 (0.69-0.99)	138:125	0.42	29	0.0	0.016	135:108	0.083
rs17298537	20,765,554											30	3.6	0.236	61:48	0.21
rs7129413	20,769,224	28	7.3	0.311	0.13	0.13	0.70	0.76	0.95 (0.77-1.16)	85:76	0.48					
rs1158547	20,771,723	29	2.5	0.539	0.33	0.32	0.34	0.37	0.96 (0.81-1.15)	167:128	0.023	31	6.2	0.931	151:118	0.044
rs4923055	20,779,494											32	7.8	0.555	148:114	0.036
rs11601634	20,791,637	30	19.9	0.539	0.22	0.21	0.35	0.38	0.89 (0.75-1.07)	118:109	0.55	33	12.1	0.454	100:73	0.040
rs1519735	20,791,833	31	0.2	0.069	0.35	0.32	0.027	0.057	0.87 (0.73-1.04)	170:136	0.052	34	0.2	0.081	160:125	0.038
rs7109624	20,792,580	32	0.7	0.035	0.05	0.05	0.54	0.11	1.04 (0.77-1.40)	33:31	0.80	35	0.7	0.012	29:21	0.26
rs7130897	20,796,450	33	3.9	0.416	0.16	0.16	0.75	0.88	0.96 (0.79-1.16)	100:89	0.42	36	3.9	0.300	85:70	0.23
rs435001	20,800,510	34	4.1	0.285	0.12	0.13	0.25	0.50	1.12 (0.91-1.38)	82:78	0.75	37	4.1	0.018	76:67	0.45
rs17298565	20,806,806	35	6.3	0.004	0.04	0.04	0.14	0.16	1.3 (0.94-1.80)	31:29	0.80					
rs1914984	20,807,913	36	1.1	0.013	0.08	0.08	0.91	0.56	0.98 (0.77-1.25)	53:53	1.00					
rs2680989	20,809,773	37	1.9	0.511	0.12	0.12	0.62	0.49	0.97 (0.79-1.20)	82:67	0.22	38	9.3	0.450	69:63	0.60
rs919473	20,822,486	38	12.7	0.755	0.18	0.18	0.71	0.44	1 (0.83-1.21)	109:93	0.26	39	12.7	0.749	99:88	0.42
rs7114248	20,824,095	39	1.6	0.998	0.22	0.22	0.79	0.89	1.04 (0.86-1.24)	116:98	0.22	40	1.6	1.000	113:109	0.79
rs1429799	20,824,886	40	0.8	0.152	0.21	0.22	0.78	0.80	1.04 (0.87-1.25)	125:109	0.30	41	0.8	0.001	113:109	0.79
NELL1_03	20,825,826											42	0.9	0.001	4:3	0.71
rs1346690	20,827,819	41	2.9	0.031	0.19	0.18	0.80	0.72	1 (0.83-1.20)	125:105	0.19					
rs1367002	20,828,374	42	0.6	0.448	0.11	0.12	0.45	0.32	1.12 (0.91-1.39)	69:68	0.93	43	2.5	0.495	71:67	0.73
rs11025788	20,829,897	43	1.5	0.406	0.21	0.22	0.62	0.70	1.07 (0.89-1.28)	125:107	0.24	44	1.5	0.001	119:111	0.60
rs7121400	20,831,014	44	1.1	0.231	0.10	0.10	0.93	0.53	1.02 (0.82-1.27)	80:62	0.13					
rs12293297	20,832,922	45	1.9	0.102	0.02	0.03	0.00095	0.0061	1.92 (1.26-2.91)	20:19	0.87					
rs1429794	20,838,013	46	5.1	0.439	0.18	0.17	0.55	0.69	0.96 (0.80-1.16)	109:94	0.29	45	8.1	0.012	108:101	0.63
rs6483735	20,844,275	47	6.3	0.805	0.21	0.19	0.10	0.19	0.89 (0.74-1.06)	130:106	0.12					
rs10766733	20,844,335	48	0.1	0.212	0.24	0.23	0.33	0.25	0.96 (0.80-1.15)	148:118	0.066					
rs919476	20,845,201	49	0.9	0.012	0.07	0.06	0.19	0.19	0.87 (0.66-1.14)	53:34	0.042	46	7.2	0.151	33:26	0.36
rs10766735	20,846,648	50	1.4	0.444	0.17	0.15	0.068	0.16	0.86 (0.71-1.04)	93:84	0.50					
rs4923128	20,848,373	51	1.7	0.998	0.29	0.27	0.18	0.34	0.92 (0.77-1.09)	166:132	0.049					
rs7109004	20,849,339	52	1.0	0.167	0.29	0.27	0.21	0.43	0.91 (0.77-1.09)	162:131	0.070	47	4.1	0.251	130:127	0.85
rs1549717	20,867,190	53	17.9	0.477	0.14	0.14	0.80	0.96	0.98 (0.80-1.20)	102:80	0.10	48	17.9	0.586	88:81	0.59
rs4296038	20,878,420											49	11.2	0.883	118:111	0.64
rs2082080	20,891,024	54	23.8	0.135	0.25	0.24	0.85	0.55	1.02 (0.85-1.22)	155:130	0.14	50	12.6	0.078	119:115	0.79
rs2293241	20,905,691											51	14.7	0.889	136:130	0.71
rs1880088	20,915,761	55	24.7	0.141	0.25	0.27	0.14	0.029	1.05 (0.88-1.25)	155:143	0.49	52	10.1	0.153	130:126	0.80
rs8176786	20,915,970	56	0.2	0.009	0.05	0.05	0.63	0.27	1.02 (0.76-1.38)	39:31	0.34	53	0.2	0.013	37:30	0.39
rs1880084	20,927,365	57	11.4	0.060	0.27	0.26	0.71	0.63	0.94 (0.79-1.12)	146:117	0.074	54	11.4	0.039	138:135	0.86
rs952696	20,935,188											55	7.8	0.523	110:103	0.63
rs10833417	20,945,048	58	17.7	0.326	0.26	0.29	0.018	0.045	1.18 (0.99-1.41)	155:143	0.49	56	9.9	0.401	142:141	0.95
rs10500884	20,955,364	59	10.3	0.997	0.12	0.12	0.94	0.99	0.99 (0.81-1.23)	75:71	0.74	57	10.3	1.000	72:72	1.00
rs1400373	20,956,134	60	0.8	0.015	0.12	0.12	0.98	0.94	1.01 (0.82-1.24)	76:71	0.68	58	0.8	0.041	73:73	1.00
rs10766756	20,966,312											59	10.2	0.081	82:78	0.75
rs10500885	20,971,636	61	15.5	0.000	0.10	0.12	0.049	0.022	1.3 (1.05-1.60)	77:75	0.87					
rs2896623	20,973,973	62	2.3	0.565	0.16	0.15	0.33	0.19	0.87 (0.72-1.06)	93:81	0.36					
rs10500886	20,976,742	63	2.8	0.922	0.24	0.22	0.11	0.29	0.87 (0.73-1.04)	127:115	0.44	60	10.4	0.544	128:117	0.48
rs12791452	20,986,789											61	10.0	0.476	91:80	0.40
rs11025878	20,998,335	64	21.6	0.018	0.24	0.23	0.31	0.61	0.92 (0.77-1.10)	126:122	0.80	62	11.5	0.039	122:122	1.00
rs4922728	21,009,790	65	11.5	0.246	0.19	0.19	0.74	0.63	1 (0.83-1.20)	118:109	0.55	63	11.5	0.207	107:106	0.95
rs7937542	21,017,772	66	8.0	0.308	0.44	0.44	0.78	0.25	0.94 (0.78-1.14)	177:170	0.71	64	8.0	0.331	181:168	0.49
rs10833444	21,029,035	67	11.3	0.178	0.24	0.25	0.49	0.79	1.06 (0.89-1.27)	125:121	0.80	65	11.3	0.156	128:120	0.61
rs9666195	21,038,467	68	9.4	0.159	0.35	0.35	0.56	0.78	1.07 (0.89-1.27)	153:143	0.56	66	9.4	0.188	156:147	0.61
rs1543153	21,047,961	69	9.5	0.200	0.23	0.23	0.82	0.96	1.03 (0.86-1.23)	130:126	0.80	67	9.5	0.214	124:117	0.65
rs7932820	21,056,960	70	9.0	0.152	0.42	0.42	0.94	0.86	1.02 (0.85-1.23)	164:163	0.96	68	9.0	0.156	165:161	0.82
rs12417046	21,066,312	71	9.4	0.239	0.19	0.18	0.17	0.17	0.92 (0.76-1.11)	111:108	0.84	69	9.4	0.254	107:102	0.73

rs2403652	21,076,903	72	10.6	0.273	0.47	0.48	0.80	0.29	1.12 (0.92-1.36)	180:176	0.83	70	10.6	0.284	176:162	0.45
rs7933049	21,087,224	73	10.3	0.097	0.21	0.21	0.58	0.25	1 (0.84-1.20)	122:121	0.95	71	10.3	0.098	119:114	0.74
rs4922753	21,096,666	74	9.4	0.967	0.29	0.29	0.91	0.98	0.99 (0.83-1.17)	147:136	0.51	72	9.4	0.959	132:128	0.80
rs10766767	21,108,451	75	11.8	0.143	0.30	0.29	0.91	0.99	0.99 (0.83-1.19)	150:140	0.56	73	11.8	0.120	130:126	0.80
rs6483748	21,137,843	76	29.4	0.642	0.30	0.29	0.71	0.37	1.02 (0.86-1.22)	146:133	0.44	74	29.4	0.620	142:120	0.17
rs4475918	21,147,706	77	9.9	0.025	0.40	0.38	0.35	0.39	0.97 (0.81-1.17)	154:145	0.60	75	9.9	0.543	163:148	0.40
rs4923403	21,156,380											76	8.7	0.125	142:121	0.20
rs1453983	21,166,668	78	19.0	0.647	0.25	0.25	0.95	0.81	0.98 (0.82-1.17)	134:124	0.53	77	10.3	0.627	145:118	0.096
rs1454003	21,175,735	79	9.1	0.149	0.17	0.19	0.21	0.46	1.11 (0.92-1.34)	108:94	0.32	78	9.1	0.075	113:101	0.41
rs1823843	21,185,111	80	9.4	0.251	0.03	0.03	0.85	0.36	1.07 (0.75-1.53)	26:24	0.78	79	9.4	0.157	15:9	0.22
rs1945327	21,190,821	81	5.7	0.028	0.10	0.10	0.61	0.83	1.05 (0.84-1.31)	69:66	0.80	80	5.7	0.023	60:43	0.094
rs1453988	21,201,279	82	10.5	0.983	0.31	0.32	0.47	0.75	1.07 (0.90-1.28)	170:152	0.32	81	10.5	0.985	149:149	1.00
rs1670638	21,207,776	83	6.5	0.033	0.32	0.32	0.69	0.88	1.04 (0.88-1.25)	170:153	0.34	82	6.5	0.041	149:148	0.95
rs1670640	21,218,026	84	10.3	0.026	0.46	0.47	0.74	0.15	1.15 (0.94-1.39)	190:181	0.64	83	10.3	0.031	166:166	1.00
rs1454008	21,230,114	85	12.1	0.670	0.41	0.42	0.53	0.74	1.03 (0.85-1.24)	162:152	0.57	84	12.1	0.699	159:150	0.61
rs1791822	21,240,131	86	10.0	0.026	0.46	0.46	0.65	0.12	0.93 (0.77-1.13)	169:166	0.87	85	10.0	0.025	161:159	0.91
rs10500901	21,240,719	87	0.6	0.028	0.03	0.03	0.84	0.98	1.04 (0.72-1.50)	25:22	0.66	86	0.6	0.025	25:20	0.46
rs1453990	21,249,027	88	8.3	0.484	0.47	0.48	0.57	0.48	0.99 (0.81-1.20)	169:167	0.91	87	8.3	0.466	160:155	0.78
rs716577	21,262,140	89	13.1	0.957	0.37	0.36	0.46	0.62	0.97 (0.81-1.16)	179:164	0.42	88	13.1	0.922	166:159	0.70
rs6483756	21,267,196	90	5.1	0.752	0.38	0.37	0.24	0.46	0.93 (0.78-1.11)	175:160	0.41	89	5.1	0.766	166:158	0.66
rs10833498	21,277,881	***91***	***10.7***	***0.621***	***0.32***	***0.31***	***0.32***	***0.60***	***0.92 (0.77-1.10)***	***175:135***	***0.023***	90	10.7	0.607	161:151	0.57
rs4335544	21,286,758	92	8.9	0.756	0.41	0.41	0.83	0.86	1.01 (0.84-1.21)	177:165	0.52	91	8.9	0.771	176:165	0.55
rs1349818	21,292,747	93	6.0	0.001	0.49	0.47	0.28	0.37	0.96 (0.79-1.17)	171:165	0.74	92	6.0	0.000	178:161	0.36
rs4399327	21,302,941	94	10.2	0.001	0.48	0.49	0.52	0.093	1.19 (0.97-1.45)	176:176	1.00	93	10.2	0.026	182:165	0.36
rs2187522	21,313,688	95	10.7	0.160	0.49	0.49	0.91	0.73	0.94 (0.77-1.16)	174:153	0.25	94	10.7	0.121	193:171	0.25
rs11026036	21,323,317	96	9.6	0.132	0.36	0.34	0.44	0.73	0.93 (0.78-1.11)	163:160	0.87	95	9.6	0.149	173:154	0.29
rs7126959	21,333,727	97	10.4	0.015	0.29	0.28	0.32	0.59	0.93 (0.78-1.10)	142:141	0.95	96	10.4	0.017	154:130	0.15
rs1945404	21,343,840	98	10.1	0.031	0.37	0.38	0.81	0.13	0.93 (0.78-1.12)	175:175	1.00	97	10.1	0.030	158:147	0.53
rs4151056	21,349,063	99	5.2	0.006	0.05	0.06	0.068	0.18	1.29 (0.96-1.73)	39:32	0.41	98	5.2	0.007	31:29	0.80
rs10833520	21,352,936	100	3.9	0.009	0.20	0.20	0.79	0.47	0.94 (0.78-1.13)	123:103	0.18	99	3.9	0.008	125:120	0.75
rs1945443	21,365,895	101	13.0	0.009	0.34	0.35	0.58	0.25	0.98 (0.82-1.17)	157:148	0.61	100	13.0	0.007	154:143	0.52
rs4539321	21,375,322	***102***	***9.4***	***0.023***	***0.46***	***0.45***	***0.45***	***0.033***	***0.82 (0.68-1.00)***	***166:158***	***0.66***	101	9.4	0.019	178:160	0.33
rs11026072	21,385,901	103	10.6	0.169	0.33	0.32	0.47	0.58	0.92 (0.77-1.09)	177:144	0.066	102	10.6	0.124	165:155	0.58
rs7943922	21,394,268	104	8.4	0.081	0.50	0.52	0.085	0.22	1.18 (0.96-1.45)	190:166	0.20	103	8.4	0.132	177:171	0.75
rs11026079	21,406,963	105	12.7	0.065	0.25	0.26	0.59	0.50	1.08 (0.91-1.29)	125:125	1.00	104	12.7	0.054	145:141	0.81
rs7110569	21,418,064	106	11.1	0.030	0.17	0.17	0.88	0.97	1.01 (0.83-1.22)	97:89	0.56	105	11.1	0.026	77:68	0.45
rs1945408	21,428,394	107	10.3	0.125	0.14	0.14	0.88	0.62	0.96 (0.78-1.17)	91:79	0.36	106	10.3	0.139	90:86	0.76
rs7945802	21,438,700	108	10.3	0.445	0.43	0.42	0.66	0.48	0.91 (0.76-1.10)	166:162	0.83	107	10.3	0.132	172:159	0.47
rs4343021	21,446,511											108	7.8	0.113	89:85	0.76
rs10766821	21,458,271	109	19.6	0.097	0.38	0.39	0.72	0.91	1.02 (0.85-1.22)	169:158	0.54	109	11.8	0.096	159:150	0.61
rs7116826	21,472,159	110	13.9	0.027	0.15	0.15	0.44	0.60	1.06 (0.87-1.29)	103:91	0.39	110	13.9	0.040	98:94	0.77
rs10219188	21,479,105	111	6.9	0.465	0.47	0.48	0.48	0.74	1.08 (0.89-1.31)	170:167	0.87	111	6.9	0.444	185:175	0.60
rs6483774	21,491,019	112	11.9	0.244	0.49	0.50	0.55	0.81	1.07 (0.87-1.31)	181:180	0.96	112	11.9	0.227	189:171	0.34
rs10766829	21,499,146	113	8.1	0.836	0.41	0.43	0.45	0.41	1.12 (0.93-1.35)	173:171	0.91	113	8.1	0.058	171:166	0.79
rs6483779	21,509,362											114	10.2	0.067	48:34	0.12
rs7927068	21,516,670											115	7.3	0.899	168:163	0.78
rs7926887	21,523,755	114	24.6	0.599	0.39	0.40	0.53	0.54	1.1 (0.92-1.32)	171:170	0.96	116	7.1	0.527	178:168	0.59
rs4319515	21,534,943	115	11.2	0.202	0.49	0.52	0.14	0.34	1.14 (0.93-1.40)	174:173	0.96	117	11.2	0.202	180:148	0.077
rs8176789	21,538,381	116	3.4	0.426	0.17	0.17	0.86	0.50	0.98 (0.81-1.19)	105:104	0.94	***118***	***3.4***	***0.206***	***124:92***	***0.030***
rs4320947	21,544,279											119	5.9	0.100	177:147	0.096
rs4922850	21,557,214	117	18.8		0.07	0.08	0.30	0.097	1.09 (0.85-1.39)	62:56	0.58	120	12.9		72:60	0.30
