# Supplementary material for: Systematic Association Mapping Identifies NELL1 as a Novel IBD Disease Gene
Source: PLoS One. 2007 Aug 8;2(8):e691. doi: 10.1371/journal.pone.0000691 (PMC1933598; doi:10.1371/journal.pone.0000691)
Supplement: Table S4 — Summary of the mutation detection of NELL1. All 21 exons plus the promoter were resequenced in 47 unrelated Crohn disease patients. Five not yet annotated polymorphisms were identified, including the two nonsynonymous SNPs NELL1_02 and NELL1_03. Twenty-sex annotated SNPs were verified. (0.14 MB PDF) [file pone.0000691.s012.pdf]

Supplementary Table 4: Summary of the mutation detection of *NELL1*. All 21 exons plus the promoter were resequenced in 47 unrelated Crohn disease patients. Five not yet annotated polymorphisms were identified, including the two nonsynonymous SNPs *NELL1_02* and *NELL1_03*. Twenty-sex annotated SNPs were verified.

#	SNP ID	position (build 35)	SNP type	sequence
1	NELL1_01	20647024	Promoter	CTGCCCTGCAGAATGAGAAGGTTTGCAAATAGACTTCCCAAACCCCAACCAC AGCTCGCTCCGCCTCGAGGACCCCTTTTCTGCACCCCCACCTCAGCGC**[C/A]**C TCTTCCTGCACCCACAAAGAGAGTACTCAGTCATAGGGGTTCAACAGGAGAG AGGAGACAGAAGGTACAGGCGGTGAGCAGGGACTCAGCCATCATCCC
2	NELL1_02	20825777	nonsynonymous R→S	AAACAAATGTTTGTTCCTTTACATACAGCTATTTTGAACTGGAGAGCAGTGG CCTGAGGGATGAGATTCGGTATCACTACATACACAATGGGAAGCCAAG**[G/C]** ACAGAGGCACTTCCTTACCGCATGGCAGATGGACAATGGCACAAGGTTGCAC TGTCAGTTAGCGCCTCTCATCTCCTGCTCCATGTCGACTGTAACAGGT
3	NELL1_03	20825826	nonsynonymous A→T	TGGCCTGAGGGATGAGATTCGGTATCACTACATACACAATGGGAAGCCAAG GACAGAGGCACTTCCTTACCGCATGGCAGATGGACAATGGCACAAGGTT**[G/ A]**CACTGTCAGTTAGCGCCTCTCATCTCCTGCTCCATGTCGACTGTAACAGGT ATTTCTTTGTCTTTGAGTGTTGCTGATTCTGCCCTTGAAATCAAGCAAT
4	NELL1_04	21538300	Intronic	AGCACAAGGTAAATGGTCGATGATGTTAGTTAATGCTACTGCAAATGATGGT TGTTTGTTCTCTAAGTTCCCTTGGCTAGATTCTAAGCTTCTGTGCTTC**[C/A]**TA TATTGCAGACATTGATGAATGTGCCTTAAGAACTCACACCTGTTGGAACGAT TCTGCCTGCATCAACCTGGCAGGGGGCTTTGACTGTCTCTGCCCCT
5	NELL1_05	20647336	Promoter	ATACCCACCCGCGAGGGCTCGGCGGCTTTTCGACTCGGCGGGGATGAACTGT GGCAACTTCGGCAGCCCCCACCGCGGTGCGGAAGTAAAGAGGGCAACA**[T/C]** TGGCGACTGCGGCTCGGAGGGGCTGGAGCGCGTGAAGCCGTGGGGGCGCCG TGCGCCTCCCGCTCTCTCGTTTCGGCCGCAGGTCCTGGGACTCCGACTT
6	rs1715283	20646687	Promoter	-
7	rs3808993	20646953	Promoter	-
8	rs1793003	20655970	Intronic	-
9	rs2280362	20761694	Intronic	-
10	rs8176785	20761862	nonsynonymous R→Q	-
11	rs2280363	20761911	synonymous	-
12	rs3740874	20896231	Intronic	-
13	rs1429785	20897351	Intronic	-
14	rs2293241	20905691	Intronic	-

15	rs1880088	20915761	Intronic	-
16	rs8176786	20915970	nonsynonymous R→W	-
17	rs1880087	20916005	Intronic	-
18	rs8176791	20916041	Intronic	-
19	rs1880086	20916058	Intronic	-
20	rs8176796	20925428	Intronic	-
21	rs2280584	20938470	Intronic	-
22	rs3758810	21091662	Intronic	-
23	rs1670638	21207776	Intronic	-
24	rs8176792	21349039	synonymous	-
25	rs4151056	21349063	synonymous	-
26	rs7119525	21538074	Intronic	-
27	rs7119475	21538241	Intronic	-
28	rs8176789	21538381	synonymous	-
29	rs8176790	21538573	Intronic	-
30	rs4922847	21548804	Intronic	-
31	rs8176793	21549083	Intronic	-
