# Supplementary material for: Systematic Association Mapping Identifies NELL1 as a Novel IBD Disease Gene
Source: PLoS One. 2007 Aug 8;2(8):e691. doi: 10.1371/journal.pone.0000691 (PMC1933598; doi:10.1371/journal.pone.0000691)
Supplement: Table S5 — Primer sequences used for the mutation detection of NELL1. (0.13 MB DOC) [file pone.0000691.s013.doc]

**Supplementary Table 5:** Primer sequences used for the mutation detection of *NELL1*.

| Region | Primer | Sequence | Amplicon |
|---|---|---|---|
| Promoter | NEL_pro2_f | 5'-TGTTAGTAGGACAAATAGGAAGTGGGA-3' | |
| | NEL_pro2_r | 5'-GCCGAGTCGAAAAGCCG-3' | 673 bp |
| | NEL_pro1_f | 5'-TGACAGAGCGAATCCCGAGTAAT-3' | |
| | NEL_pro1_r | 5'-AAGCTAGGTGGAAGCAAATGAGC-3' | 663 bp |
| Exon 01 | NELL1_ex01_F | 5'-CGCAACAAGCCACAGTAGCC-3' | |
| (208 bp) | NELL1_ex01_R | 5'-CGAGCAGGGCAAAGAGATCC-3' | 517 bp |
| Add. Exon | NEL_ade_f | 5'-GGAGTGGTCTGGAGAACTGGTCT-3' | |
| (84 bp) | NEL_ade_r | 5'-GTGCCCTCAATCCAGAAAGTATTG-3' | 423 bp |
| Exon 02 | NELL1_ex02_F | 5'-AGCGGGGTAAGGGAGCAAAG-3' | |
| (129 bp) | NELL1_ex02_R | 5'-TGATCTCTAATGCCTCCCTCCTG-3' | 481 bp |
| Exon 03 | NELL1_ex03_F | 5'-GAAACTTGCAATCTGGATTCTTTGG-3' | |
| (151 bp) | NELL1_ex03_R | 5'-TGGTCCTTGGAGAGCAAACAAG-3' | 515 bp |
| Exon 04 | NELL1_ex04_F | 5'-TGCCACGAGGGTTCTCAGAC-3' | |
| (171 bp) | NELL1_ex04_R | 5'-GCATGCCTAAGGTCCCATTTG-3' | 515 bp |
| Exon 05 | NELL1_ex05_F | 5'-GCCCAGATGATGTGTCCAAG-3' | |
| (97 bp) | NELL1_ex05_R | 5'-AAACCAGTTTATTCATGTCAAGCAC-3' | 464 bp |
| Exon 06 | NELL1_ex06_F | 5'-GCCAGCTCACCCTTTGAATG-3' | |
| (73 bp) | NELL1_ex06_R | 5'-TCTTTCACTATCTCCAGCACCTCAG-3' | 331 bp |
| Exon 07 | NELL1_ex07_F | 5'-TCCAGACCTGAAATCCTCTGTG-3' | |
| (83 bp) | NELL1_ex07_R | 5'-GCATCAAAGACAGGAATGGTTATG-3' | 430 bp |
| Exon 08 | NELL1_ex08_F | 5'-TGTGGGCTTGAATGGAAAGC-3' | |
| (135 bp) | NELL1_ex08_R | 5'- TTGCTTCACAATTGCTCGTATGG-3' | 589 bp |
| Exon 09 | NELL1_ex09_F | 5'-GTGGTCCTGGAGTGGACTGG-3' | |
| (103 bp) | NELL1_ex09_R | 5'-CATGTTAAGTGTCTGCCACATTGC-3' | 433 bp |
| Exon 10 | NELL1_ex10_F | 5'-AGGTCTGCCTGGGAGATTTAAGG-3' | |

| | | | |
|---|---|---|---|
| (74 bp) | NELL1_ex10_R | 5'-ATCCAACCCACCGCAGAGAG-3' | 397 bp |
| Exon 11 | NELL1_ex11_F | 5'-TGTGGCATTATCGTTTTTAGTGGAATC-3' | |
| (100 bp) | NELL1_ex11_R | 5'-GGAGCAGCGCACAGAGTTTTG-3' | 367 bp |
| Exon 12 | NELL1_ex12_F | 5'-GGAAAGCCTCTTACGCCTTGG-3' | |
| (129 bp) | NELL1_ex12_R | 5'-TTCAAAGGTCTTGCTTTCTCATGC-3' | 526 bp |
| Exon 13 | NELL1_ex13_F | 5'-TCAGCTCAGGATGGGATCTAACG-3' | |
| (126 bp) | NELL1_ex13_R | 5'-TGGGAGTGGAAATCAATTATGCAG-3' | 500 bp |
| Exon 14 | NELL1_ex14_F | 5'-GCGCATAGTAAGGTACTGACAAGTGG-3' | |
| (123 bp) | NELL1_ex14_R | 5'-TTTCCCTGACGCACAGTACCC-3' | 408 bp |
| Exon 15 | NELL1_ex15_F | 5'-GCATGCCAGCCCTATGCTAAAC-3' | |
| (96 bp) | NELL1_ex15_R | 5'-AGCTCCATCCACCGTCACAAC-3' | 467 bp |
| Exon 16 | NELL1_ex16_F | 5'-TGGGATTTGCTTCTTCCAGTGAC-3' | |
| (141 bp) | NELL1_ex16_R | 5'-GCATGATCCCTGGCCTAATCC-3' | 461 bp |
| Exon 17 | NELL1_ex17_F | 5'-TCTCCCACACAGAGCAGAACTGAC-3' | |
| (194 bp) | NELL1_ex17_R | 5'-TCAAAGCAGGCTTCCCTACCC-3' | 551 bp |
| Exon 18 | NELL1_ex18_F | 5'-CTGCTCTGCAAGCAAAATCAGG-3' | |
| (177 bp) | NELL1_ex18_R | 5'-CTTAGCCACAGGCCCCACAG-3' | 492 bp |
| Exon 19 | NELL1_ex19_F | 5'-GATCAGCAAAAGCATTCTGAAAGAAG-3' | |
| (225 bp) | NELL1_ex19_R | 5'-GCCAGAGTTTCCACCATGTCC-3' | 537 bp |
| Exon 20 | NELL1_ex20_F | 5'-GGGTCTGTCAGTCCTTCCTTTC-3' | |
| (712 bp) | NELL1_ex20_R | 5'-TGCAAATGATCTGATAAGGGAAAC-3' | 534 bp |
