# Supplementary material for: Systematic Association Mapping Identifies NELL1 as a Novel IBD Disease Gene
Source: PLoS One. 2007 Aug 8;2(8):e691. doi: 10.1371/journal.pone.0000691 (PMC1933598; doi:10.1371/journal.pone.0000691)
Supplement: Table S6 — Fine mapping of the CD association signal at the 5p13.1 locus in replication panels B and D. The highlighting and the column headers are the same as described in Table Legend S3. (0.16 MB PDF) [file pone.0000691.s014.pdf]

**Supplementary Table 6:** Fine mapping of the CD association signal at the *5p13.1* locus in replication panels B and D. The highlighting and the column headers are the same as described in table legend S3.

| | | Panel B | | | | | | | | | | Panel D | | | | |
|---|---|---|---|---|---|---|---|---|---|---|---|---|---|---|---|---|
| **dbSNP ID** | **Position** | **#** | **Distance [kb]** | **LD [$r^2$]** | **$MAF_{co}$** | **$MAF_{ca}$** | **$p_{CCA}$** | **$p_{CCG}$** | **OR (95% CI)** | **T:U** | **$p_{TDT}$** | **#** | **Distance [kb]** | **LD [$r^2$]** | **T:U** | **$p_{TDT}$** |
| rs350031 | 40,223,211 | | | | | | | | | | | 1 | - | 0.004 | 128:118 | 0.52 |
| rs16869727 | 40,229,955 | | | | | | | | | | | 2 | 6.7 | 0.013 | 69:60 | 0.43 |
| rs353325 | 40,246,690 | | | | | | | | | | | 3 | 16.7 | 0.008 | 80:74 | 0.63 |
| rs183046 | 40,269,981 | | | | | | | | | | | 4 | 23.3 | 0.021 | 99:95 | 0.77 |
| rs17224401 | 40,275,446 | | | | | | | | | | | 5 | 5.5 | 0.001 | 119:109 | 0.51 |
| rs350052 | 40,290,787 | | | | | | | | | | | 6 | 15.3 | 0.002 | 112:104 | 0.59 |
| rs189814 | 40,292,083 | ***1*** | ***-*** | ***0.400*** | ***0.49*** | ***0.47*** | ***0.25*** | ***0.21*** | ***0.98 (0.80-1.20)*** | ***201:162*** | ***0.041*** | | | | | |
| rs238772 | 40,292,388 | | | | | | | | | | | 7 | 1.6 | 0.000 | 1:0 | 0.32 |
| rs2371680 | 40,294,274 | | | | | | | | | | | 8 | 1.9 | 0.000 | 163:131 | 0.062 |
| rs17823523 | 40,294,659 | | | | | | | | | | | 9 | 0.4 | 0.000 | 127:118 | 0.57 |
| rs348609 | 40,296,686 | | | | | | | | | | | 10 | 2.0 | 0.256 | 111:105 | 0.68 |
| rs6881572 | 40,299,361 | | | | | | | | | | | 11 | 2.7 | 0.434 | 188:170 | 0.34 |
| rs1348612 | 40,301,661 | | | | | | | | | | | 12 | 2.3 | 0.002 | 163:131 | 0.062 |
| rs1445010 | 40,304,133 | | | | | | | | | | | ***13*** | ***2.5*** | ***0.002*** | ***86:55*** | ***0.0090*** |
| rs755989 | 40,307,432 | | | | | | | | | | | 14 | 3.3 | 0.433 | 163:131 | 0.062 |
| rs2120857 | 40,310,215 | | | | | | | | | | | 15 | 2.8 | 0.434 | 190:170 | 0.29 |
| rs971212 | 40,311,451 | | | | | | | | | | | 16 | 1.2 | 1.000 | 163:131 | 0.062 |
| rs4957262 | 40,313,082 | | | | | | | | | | | 17 | 1.6 | 1.000 | 163:130 | 0.054 |
| rs2860001 | 40,314,606 | | | | | | | | | | | 18 | 1.5 | 0.009 | 163:131 | 0.062 |
| rs1445011 | 40,315,959 | ***2*** | ***23.9*** | ***0.000*** | ***0.28*** | ***0.25*** | ***0.052*** | ***0.078*** | ***0.88 (0.74-1.05)*** | ***163:120*** | ***0.011*** | 19 | 1.4 | 0.009 | 147:119 | 0.086 |
| rs1445012 | 40,317,863 | | | | | | | | | | | 20 | 1.9 | 1.000 | 162:131 | 0.070 |
| rs1373696 | 40,319,579 | | | | | | | | | | | 21 | 1.7 | 1.000 | 162:131 | 0.070 |
| rs17225890 | 40,321,140 | | | | | | | | | | | 22 | 1.6 | 0.003 | 162:131 | 0.070 |
| rs348620 | 40,322,578 | | | | | | | | | | | 23 | 1.4 | 0.029 | 63:45 | 0.083 |
| rs4245975 | 40,324,635 | | | | | | | | | | | 24 | 2.1 | 0.996 | 107:89 | 0.20 |
| rs12518811 | 40,327,354 | | | | | | | | | | | 25 | 2.7 | 0.010 | 107:88 | 0.17 |
| rs348584 | 40,329,072 | | | | | | | | | | | 26 | 1.7 | 0.003 | 139:135 | 0.81 |
| rs348589 | 40,331,377 | | | | | | | | | | | 27 | 2.3 | 0.350 | 90:79 | 0.40 |
| rs1564269 | 40,338,324 | | | | | | | | | | | 28 | 6.9 | 0.350 | 154:151 | 0.86 |
| rs348571 | 40,339,742 | | | | | | | | | | | 29 | 1.4 | 0.394 | 90:79 | 0.40 |
| rs180900 | 40,342,058 | | | | | | | | | | | 30 | 2.3 | 0.004 | 108:94 | 0.32 |
| rs10512731 | 40,344,547 | | | | | | | | | | | 31 | 2.5 | 0.050 | 124:110 | 0.36 |
| rs348560 | 40,348,617 | | | | | | | | | | | 32 | 4.1 | 0.005 | 90:79 | 0.40 |
| rs6871840 | 40,350,199 | | | | | | | | | | | 33 | 1.6 | 0.006 | 22:14 | 0.18 |
| rs4957127 | 40,351,767 | | | | | | | | | | | 34 | 1.6 | 0.257 | 77:65 | 0.31 |
| rs1445002 | 40,355,634 | ***3*** | ***39.7*** | ***0.048*** | ***0.14*** | ***0.19*** | ***3.94E-05*** | ***0.00021*** | ***1.45 (1.19-1.76)*** | ***109:81*** | ***0.042*** | | | | | |
| rs348601 | 40,355,763 | | | | | | | | | | | 35 | 4.0 | 0.400 | 179:157 | 0.23 |
| rs348599 | 40,356,915 | | | | | | | | | | | 36 | 1.2 | 0.394 | 120:102 | 0.23 |
| rs348595 | 40,359,471 | | | | | | | | | | | 37 | 2.6 | 0.062 | 176:158 | 0.32 |
| rs10043093 | 40,359,977 | | | | | | | | | | | 38 | 0.5 | 0.017 | 105:98 | 0.62 |
| rs348593 | 40,360,289 | | | | | | | | | | | 39 | 0.3 | 0.993 | 118:102 | 0.28 |
| rs2034185 | 40,361,415 | | | | | | | | | | | 40 | 1.1 | 1.000 | 117:100 | 0.25 |
| rs443583 | 40,368,840 | ***4*** | ***13.2*** | ***0.250*** | ***0.21*** | ***0.18*** | ***0.061*** | ***0.16*** | ***0.84 (0.70-1.01)*** | ***134:99*** | ***0.022*** | 41 | 7.4 | 0.993 | 118:101 | 0.25 |
| rs394213 | 40,369,508 | | | | | | | | | | | 42 | 0.7 | 0.990 | 118:102 | 0.28 |
| rs7726744 | 40,379,033 | | | | | | | | | | | 43 | 9.5 | 0.291 | 118:101 | 0.25 |
| rs12518245 | 40,381,478 | 5 | 12.6 | 0.181 | 0.06 | 0.05 | 0.50 | 0.70 | 0.92 (0.70-1.22) | 38:37 | 0.91 | 44 | 2.4 | 0.010 | 51:43 | 0.41 |
| rs16869833 | 40,383,331 | | | | | | | | | | | ***45*** | ***1.9*** | ***0.097*** | ***113:73*** | ***0.0034*** |
| rs4286720 | 40,385,373 | | | | | | | | | | | 46 | 2.0 | 0.087 | 99:94 | 0.72 |
| rs7725639 | 40,391,593 | | | | | | | | | | | 47 | 6.2 | 0.995 | 153:147 | 0.73 |
| rs6451489 | 40,393,420 | | | | | | | | | | | 48 | 1.8 | 0.090 | 154:147 | 0.69 |
| rs12517783 | 40,395,897 | | | | | | | | | | | 49 | 2.5 | 1.000 | 100:98 | 0.89 |
| rs4957278 | 40,397,577 | | | | | | | | | | | 50 | 1.7 | 1.000 | 98:97 | 0.94 |
| rs12514900 | 40,399,633 | | | | | | | | | | | 51 | 2.1 | 0.017 | 100:98 | 0.89 |
| rs6451491 | 40,401,673 | | | | | | | | | | | 52 | 2.0 | 0.017 | 49:42 | 0.46 |
| rs12514679 | 40,402,560 | | | | | | | | | | | 53 | 0.9 | 0.186 | 98:98 | 1.00 |
| rs10472332 | 40,404,130 | | | | | | | | | | | 54 | 1.6 | 0.001 | 191:176 | 0.43 |
| rs1020361 | 40,405,439 | | | | | | | | | | | 55 | 1.3 | 0.002 | 1:0 | 0.32 |
| rs7725523 | 40,407,980 | ***6*** | ***26.5*** | ***0.359*** | ***0.25*** | ***0.23*** | ***0.14*** | ***0.32*** | ***0.87 (0.73-1.04)*** | ***158:117*** | ***0.013*** | 56 | 2.5 | 0.128 | 178:163 | 0.42 |
| rs2120854 | 40,408,891 | | | | | | | | | | | 57 | 0.9 | 0.985 | 51:41 | 0.30 |
| rs10473185 | 40,410,924 | | | | | | | | | | | 58 | 2.0 | 0.017 | 51:40 | 0.25 |
| rs16869860 | 40,413,388 | | | | | | | | | | | 59 | 2.5 | 0.016 | 98:97 | 0.94 |
| rs4957279 | 40,415,107 | | | | | | | | | | | 60 | 1.7 | 0.226 | 49:42 | 0.46 |
| rs1031168 | 40,417,377 | | | | | | | | | | | 61 | 2.3 | 0.006 | 135:126 | 0.58 |
| rs895123 | 40,419,818 | | | | | | | | | | | 62 | 2.4 | 0.000 | 143:116 | 0.093 |
| rs1002922 | 40,422,312 | | | | | | | | | | | 63 | 2.5 | 0.020 | 155:144 | 0.52 |
| rs1550761 | 40,424,886 | | | | | | | | | | | 64 | 2.6 | 0.009 | 89:88 | 0.94 |
| rs2034182 | 40,426,689 | | | | | | | | | | | 65 | 1.8 | 0.015 | 51:39 | 0.21 |
| rs4613763 | 40,428,485 | | | | | | | | | | | 66 | 1.8 | 0.087 | 98:97 | 0.94 |
| rs1992662 | 40,429,609 | *7* | *21.6* | *0.741* | *0.32* | *0.26* | *7.59E-05* | *0.00017* | *0.76 (0.63-0.91)* | *173:118* | *0.0013* | 67 | 1.1 | 1.000 | 155:143 | 0.49 |
| rs10512736 | 40,431,143 | | | | | | | | | | | 68 | 1.5 | 0.171 | 155:143 | 0.49 |
| rs16869934 | 40,433,109 | | | | | | | | | | | 69 | 2.0 | 0.078 | 110:101 | 0.54 |
| rs10941505 | 40,435,003 | | | | | | | | | | | 70 | 1.9 | 0.022 | 192:176 | 0.40 |
| rs1445009 | 40,437,446 | | | | | | | | | | | 71 | 2.4 | 0.002 | 53:47 | 0.55 |
| rs1025969 | 40,438,670 | | | | | | | | | | | ***72*** | ***1.2*** | ***0.006*** | ***78:48*** | ***0.0075*** |
| rs7723981 | 40,440,669 | | | | | | | | | | | 73 | 2.0 | 0.973 | 59:50 | 0.39 |
| rs1445006 | 40,442,602 | | | | | | | | | | | 74 | 1.9 | 0.038 | 59:48 | 0.29 |
| rs10512737 | 40,445,800 | | | | | | | | | | | 75 | 3.2 | 0.010 | 143:141 | 0.91 |
| rs6451494 | 40,447,048 | | | | | | | | | | | 76 | 1.2 | 1.000 | 178:163 | 0.42 |
| rs10473191 | 40,448,701 | | | | | | | | | | | 77 | 1.7 | 1.000 | 174:161 | 0.48 |
| rs1992660 | 40,450,824 | *8* | *21.2* | *0.474* | *0.38* | *0.32* | *4.53E-05* | *0.00021* | *0.72 (0.60-0.86)* | *194:131* | *0.00050* | 78 | 2.1 | 0.658 | 178:163 | 0.42 |
| rs10071761 | 40,451,368 | | | | | | | | | | | 79 | 0.5 | 0.078 | 148:140 | 0.64 |
| rs4957288 | 40,459,597 | | | | | | | | | | | ***80*** | ***8.2*** | ***0.045*** | ***83:56*** | ***0.022*** |
| rs1899983 | 40,460,734 | | | | | | | | | | | 81 | 1.1 | 0.145 | 178:167 | 0.55 |
| rs1445003 | 40,462,533 | | | | | | | | | | | 82 | 1.8 | 0.008 | 59:51 | 0.45 |
| rs10512738 | 40,464,558 | | | | | | | | | | | 83 | 2.0 | 0.196 | 51:51 | 1.00 |
| rs1545334 | 40,465,875 | | | | | | | | | | | 84 | 1.3 | 0.983 | 148:143 | 0.77 |
| rs4957291 | 40,467,706 | | | | | | | | | | | 85 | 1.8 | 0.698 | 148:141 | 0.68 |
| rs6878213 | 40,469,333 | | | | | | | | | | | 86 | 1.6 | 0.698 | 173:166 | 0.70 |
| rs12514415 | 40,470,793 | | | | | | | | | | | 87 | 1.5 | 0.087 | 148:141 | 0.68 |
| rs7734434 | 40,472,455 | | | | | | | | | | | 88 | 1.7 | 0.125 | 100:99 | 0.94 |

| | | | | | | | | | | | | | | | | |
|---|---|---|---|---|---|---|---|---|---|---|---|---|---|---|---|---|
| rs9292777 | 40,473,705 | | | | | | | | | | | 89 | 1.3 | 0.511 | 174:166 | 0.66 |
| rs7722414 | 40,477,100 | | | | | | | | | | | 90 | 3.4 | 0.245 | 189:179 | 0.60 |
| rs9292778 | 40,479,053 | | | | | | | | | | | 91 | 2.0 | 1.000 | 158:148 | 0.57 |
| rs11957215 | 40,481,438 | | | | | | | | | | | 92 | 2.4 | 0.000 | 158:148 | 0.57 |
| rs11954069 | 40,487,990 | | | | | | | | | | | 93 | 6.6 | 0.000 | 126:125 | 0.95 |
| rs4498294 | 40,489,279 | | | | | | | | | | | 94 | 1.3 | 0.998 | 158:147 | 0.53 |
| rs12520532 | 40,491,202 | | | | | | | | | | | 95 | 1.9 | 1.000 | 158:149 | 0.61 |
| rs11955354 | 40,493,216 | | | | | | | | | | | 96 | 2.0 | 0.181 | 159:149 | 0.57 |
| rs6451507 | 40,494,445 | | | | | | | | | | | 97 | 1.2 | 0.181 | 150:137 | 0.44 |
| rs7725387 | 40,495,506 | | | | | | | | | | | 98 | 1.1 | 0.978 | 159:149 | 0.57 |
| rs10941509 | 40,498,115 | | | | | | | | | | | 99 | 2.6 | 0.978 | 159:147 | 0.49 |
| rs4957300 | 40,499,496 | 9 | 48.7 | 0.263 | 0.33 | 0.29 | 0.0033 | 0.0061 | 0.83 (0.70-0.99) | 191:124 | 0.00020 | 100 | 1.4 | 0.998 | 159:149 | 0.57 |
| rs4957303 | 40,502,034 | | | | | | | | | | | 101 | 2.5 | 0.998 | 159:148 | 0.53 |
| rs4532399 | 40,503,029 | | | | | | | | | | | 102 | 1.0 | 0.998 | 158:148 | 0.57 |
| rs4495224 | 40,513,272 | | | | | | | | | | | 103 | 10.2 | 0.988 | 159:149 | 0.57 |
| rs6871834 | 40,515,944 | | | | | | | | | | | 104 | 2.7 | 0.227 | 159:150 | 0.61 |
| rs6882351 | 40,517,411 | | | | | | | | | | | 105 | 1.5 | 0.226 | 59:56 | 0.78 |
| rs4422570 | 40,518,098 | 10 | 18.6 | 0.056 | 0.10 | 0.10 | 0.83 | 0.75 | 1.04 (0.83-1.31) | 85:59 | 0.030 | | | | | |
| rs10941515 | 40,518,283 | | | | | | | | | | | 106 | 0.9 | 0.000 | 155:148 | 0.69 |
| rs7725052 | 40,523,027 | 11 | 4.9 | 0.572 | 0.44 | 0.37 | 3.24E-06 | 1.95E-05 | 0.71 (0.59-0.86) | 194:137 | 0.0017 | 107 | 4.7 | 0.001 | 122:109 | 0.39 |
| rs10073277 | 40,524,443 | | | | | | | | | | | 108 | 1.4 | 0.749 | 160:139 | 0.22 |
| rs9283753 | 40,526,366 | | | | | | | | | | | 109 | 1.9 | 0.443 | 184:147 | 0.042 |
| rs10041894 | 40,528,991 | | | | | | | | | | | 110 | 2.6 | 0.924 | 136:91 | 0.0028 |
| rs12520522 | 40,530,435 | | | | | | | | | | | 111 | 1.4 | 0.766 | 135:90 | 0.0027 |
| rs1505992 | 40,534,334 | | | | | | | | | | | 112 | 3.9 | 0.917 | 152:109 | 0.0078 |
| rs1553575 | 40,538,689 | 12 | 15.7 | 0.482 | 0.35 | 0.28 | 1.68E-06 | 6.37E-06 | 0.65 (0.55-0.78) | 169:132 | 0.051 | 113 | 4.4 | 1.000 | 154:112 | 0.010 |
| rs1157160 | 40,540,177 | | | | | | | | | | | 114 | 1.5 | 0.984 | 149:109 | 0.013 |
| rs4957311 | 40,542,168 | | | | | | | | | | | 115 | 2.0 | 0.984 | 155:113 | 0.010 |
| rs7734013 | 40,543,000 | | | | | | | | | | | 116 | 0.8 | 1.000 | 154:112 | 0.010 |
| rs1553577 | 40,545,764 | | | | | | | | | | | 117 | 2.8 | 1.000 | 154:112 | 0.010 |
| rs7703539 | 40,549,071 | | | | | | | | | | | 118 | 3.3 | 1.000 | 154:111 | 0.0083 |
| rs4957313 | 40,551,279 | | | | | | | | | | | 119 | 2.2 | 1.000 | 153:112 | 0.012 |
| rs10068204 | 40,555,108 | | | | | | | | | | | 120 | 3.8 | 0.494 | 154:112 | 0.010 |
| rs1876143 | 40,557,405 | | | | | | | | | | | 121 | 2.3 | 0.002 | 103:70 | 0.012 |
| rs1876142 | 40,557,462 | | | | | | | | | | | 122 | 0.1 | 0.013 | 126:114 | 0.44 |
| rs10941516 | 40,557,969 | | | | | | | | | | | 123 | 0.5 | 0.393 | 194:149 | 0.015 |
| rs1876141 | 40,561,492 | | | | | | | | | | | 124 | 3.5 | 1.000 | 154:112 | 0.010 |
| rs6873494 | 40,563,883 | | | | | | | | | | | 125 | 2.4 | 0.896 | 154:110 | 0.0068 |
| rs7718309 | 40,564,656 | 13 | 26.0 | 0.912 | 0.19 | 0.16 | 0.023 | 0.013 | 0.77 (0.63-0.93) | 109:85 | 0.085 | | | | | |
| rs4512170 | 40,566,291 | | | | | | | | | | | 126 | 2.4 | 0.551 | 142:103 | 0.013 |
| rs4957319 | 40,568,372 | | | | | | | | | | | 127 | 2.1 | 0.551 | 103:72 | 0.019 |
| rs6451517 | 40,571,930 | | | | | | | | | | | 128 | 3.6 | 0.000 | 142:103 | 0.013 |
| rs6451519 | 40,576,626 | | | | | | | | | | | 129 | 4.7 | 0.000 | 105:104 | 0.94 |
| rs7730267 | 40,584,302 | | | | | | | | | | | 130 | 7.7 | 0.018 | 63:55 | 0.46 |
| rs969258 | 40,585,511 | | | | | | | | | | | 131 | 1.2 | 0.007 | 103:72 | 0.019 |
| rs7713972 | 40,588,231 | 14 | 23.6 | 0.553 | 0.16 | 0.15 | 0.26 | 0.15 | 0.86 (0.71-1.04) | 103:83 | 0.14 | 132 | 2.7 | 0.002 | 114:110 | 0.79 |
| rs6451525 | 40,590,026 | 15 | 1.8 | 0.498 | 0.30 | 0.24 | 1.64E-05 | 4.49E-05 | 0.67 (0.56-0.80) | 152:118 | 0.039 | 133 | 1.8 | 0.009 | 32:19 | 0.069 |
| rs12697413 | 40,592,084 | | | | | | | | | | | 134 | 2.1 | 0.340 | 134:95 | 0.010 |
| rs1024098 | 40,595,144 | | | | | | | | | | | 135 | 3.1 | 0.002 | 63:55 | 0.46 |
| rs6864749 | 40,604,350 | 16 | 14.3 | 0.414 | 0.19 | 0.16 | 0.029 | 0.014 | 0.77 (0.64-0.93) | 109:87 | 0.12 | 136 | 9.2 | 0.002 | 95:87 | 0.55 |
| rs10055860 | 40,605,710 | | | | | | | | | | | 137 | 1.4 | 0.997 | 140:103 | 0.018 |
| rs11742349 | 40,608,200 | | | | | | | | | | | 138 | 2.5 | 0.250 | 139:102 | 0.017 |
| rs2055646 | 40,610,699 | | | | | | | | | | | 139 | 2.5 | 0.031 | 52:38 | 0.14 |
| rs6451529 | 40,611,106 | 17 | 6.8 | 0.712 | 0.08 | 0.08 | 0.66 | 0.68 | 0.97 (0.75-1.24) | 56:45 | 0.27 | 140 | 0.4 | 0.005 | 65:58 | 0.53 |
| rs1948902 | 40,613,715 | | | | | | | | | | | 141 | 2.6 | 0.994 | 139:103 | 0.021 |
| rs7708395 | 40,615,628 | | | | | | | | | | | 142 | 1.9 | 0.330 | 139:103 | 0.021 |
| rs4342367 | 40,619,398 | | | | | | | | | | | 143 | 3.8 | 0.988 | 68:53 | 0.17 |
| rs7705019 | 40,623,561 | | | | | | | | | | | 144 | 4.2 | 0.752 | 68:53 | 0.17 |
| rs906064 | 40,625,774 | | | | | | | | | | | 145 | 2.2 | 0.752 | 50:38 | 0.20 |
| rs7711611 | 40,628,525 | | | | | | | | | | | 146 | 2.8 | 0.964 | 68:53 | 0.17 |
| rs6878946 | 40,631,161 | | | | | | | | | | | 147 | 2.6 | 0.017 | 69:54 | 0.18 |
| rs6869535 | 40,633,375 | | | | | | | | | | | 148 | 2.2 | 0.486 | 82:68 | 0.25 |
| rs6897467 | 40,635,001 | | | | | | | | | | | 149 | 1.6 | 0.963 | 137:104 | 0.034 |
| rs906062 | 40,636,914 | | | | | | | | | | | 150 | 1.9 | 0.200 | 140:106 | 0.030 |
| rs4409138 | 40,639,362 | 18 | 28.3 | 1.000 | 0.12 | 0.10 | 0.047 | 0.14 | 0.8 (0.64-1.00) | 76:65 | 0.35 | 151 | 2.4 | 0.204 | 57:50 | 0.50 |
| rs6879632 | 40,640,517 | | | | | | | | | | | 152 | 1.2 | 0.375 | 137:105 | 0.040 |
| rs6890899 | 40,642,798 | | | | | | | | | | | 153 | 2.3 | 0.005 | 76:58 | 0.12 |
| rs16870171 | 40,643,917 | | | | | | | | | | | 154 | 1.1 | 0.005 | 29:21 | 0.26 |
| rs4957324 | 40,647,732 | | | | | | | | | | | 155 | 3.8 | 0.014 | 76:58 | 0.12 |
| rs924967 | 40,650,879 | 19 | 11.5 | 0.014 | 0.12 | 0.10 | 0.055 | 0.16 | 0.81 (0.65-1.01) | 75:65 | 0.40 | 156 | 3.1 | 0.060 | 128:121 | 0.66 |
| rs1979937 | 40,653,159 | | | | | | | | | | | 157 | 2.3 | 0.378 | 141:109 | 0.043 |
| rs10941519 | 40,655,512 | | | | | | | | | | | 158 | 2.4 | 0.140 | 191:143 | 0.0086 |
| rs4957137 | 40,656,550 | | | | | | | | | | | 159 | 1.0 | 0.004 | 78:58 | 0.086 |
| rs17834885 | 40,661,879 | | | | | | | | | | | 160 | 5.3 | 0.004 | 107:100 | 0.63 |
| rs17238368 | 40,662,946 | | | | | | | | | | | 161 | 1.1 | 0.009 | 62:45 | 0.10 |
| rs11739531 | 40,665,104 | | | | | | | | | | | 162 | 2.2 | 0.135 | 150:148 | 0.91 |
| rs6892418 | 40,667,478 | | | | | | | | | | | 163 | 2.4 | 0.223 | 125:115 | 0.52 |
| rs1395082 | 40,668,032 | | | | | | | | | | | 164 | 0.6 | 0.005 | 175:170 | 0.79 |
| rs7714305 | 40,677,005 | | | | | | | | | | | 165 | 9.0 | 0.000 | 95:83 | 0.37 |
| rs6877753 | 40,677,032 | 20 | 26.2 | 0.001 | 0.11 | 0.12 | 0.50 | 0.56 | 1.05 (0.85-1.30) | 82:74 | 0.52 | | | | | |
| rs7716982 | 40,682,317 | | | | | | | | | | | 166 | 5.3 | 0.409 | 149:148 | 0.95 |
| rs10941521 | 40,683,897 | | | | | | | | | | | 167 | 1.6 | 1.000 | 175:170 | 0.79 |
| rs10068069 | 40,687,372 | | | | | | | | | | | 168 | 3.5 | 1.000 | 175:170 | 0.79 |
| rs10071870 | 40,688,984 | | | | | | | | | | | 169 | 1.6 | 0.998 | 175:170 | 0.79 |
| rs4957334 | 40,692,609 | | | | | | | | | | | 170 | 3.6 | 0.998 | 171:167 | 0.83 |
| rs11744569 | 40,693,406 | | | | | | | | | | | 171 | 0.8 | 0.409 | 175:170 | 0.79 |
| rs2888173 | 40,695,209 | | | | | | | | | | | 172 | 1.8 | 0.001 | 149:149 | 1.00 |
| rs7717878 | 40,698,014 | | | | | | | | | | | 173 | 2.8 | 0.000 | 1:0 | 0.32 |
| rs1395085 | 40,701,679 | | | | | | | | | | | 174 | 3.7 | 0.147 | 95:89 | 0.66 |
| rs10473214 | 40,703,792 | | | | | | | | | | | 175 | 2.1 | 0.007 | 177:169 | 0.67 |
| rs1553579 | 40,704,550 | | | | | | | | | | | 176 | 0.8 | 0.007 | 92:80 | 0.36 |

| | | | | | | | | | | | | | | | | |
|---|---|---|---|---|---|---|---|---|---|---|---|---|---|---|---|---|
| rs1553578 | 40,704,941 | | | | | | | | | | | 177 | 0.4 | 0.006 | 176:170 | 0.75 |
| rs4432939 | 40,706,856 | | | | | | | | | | | 178 | 1.9 | 0.003 | 116:108 | 0.59 |
| rs4607392 | 40,709,272 | | | | | | | | | | | 179 | 2.4 | 0.476 | 164:161 | 0.87 |
| rs10066459 | 40,711,291 | | | | | | | | | | | 180 | 2.0 | 0.472 | 179:171 | 0.67 |
| rs4434423 | 40,713,439 | | | | | | | | | | | 181 | 2.1 | 0.995 | 164:160 | 0.82 |
| rs4133101 | 40,715,324 | | | | | | | | | | | 182 | 1.9 | 0.039 | 164:159 | 0.78 |
| rs2228058 | 40,717,011 | | | | | | | | | | | 183 | 1.7 | 0.043 | 41:39 | 0.82 |
| rs4546432 | 40,718,307 | 21 | 41.3 | 0.742 | 0.38 | 0.39 | 0.43 | 0.20 | 1.15 (0.96-1.38) | 187:170 | 0.37 | 184 | 1.3 | 0.678 | 173:166 | 0.70 |
| rs11957406 | 40,719,324 | | | | | | | | | | | 185 | 1.0 | 0.478 | 178:170 | 0.67 |
| rs6451535 | 40,723,788 | 22 | 5.5 | 0.349 | 0.32 | 0.32 | 0.98 | 0.27 | 1.07 (0.89-1.27) | 170:148 | 0.22 | 186 | 4.5 | 0.137 | 154:145 | 0.60 |
| rs17835256 | 40,726,418 | | | | | | | | | | | 187 | 2.6 | 0.046 | 132:127 | 0.76 |
| rs16870224 | 40,728,697 | | | | | | | | | | | 188 | 2.3 | 0.073 | 92:83 | 0.50 |
| rs4277957 | 40,735,275 | | | | | | | | | | | 189 | 6.6 | 0.767 | 156:147 | 0.61 |
| rs1057631 | 40,748,001 | | | | | | | | | | | 190 | 12.7 | 1.000 | 134:127 | 0.66 |
| rs10737963 | 40,751,910 | | | | | | | | | | | 191 | 3.9 | 0.017 | 134:127 | 0.66 |
| rs249413 | 40,757,195 | | | | | | | | | | | 192 | 5.3 | 0.031 | 79:77 | 0.87 |
| rs447810 | 40,762,558 | | | | | | | | | | | 193 | 5.4 | 0.138 | 156:149 | 0.69 |
| rs3805499 | 40,764,958 | | | | | | | | | | | 194 | 2.4 | 0.138 | 132:127 | 0.76 |
| rs249414 | 40,769,451 | | | | | | | | | | | 195 | 4.5 | 0.335 | 156:149 | 0.69 |
| rs10941525 | 40,771,683 | | | | | | | | | | | 196 | 2.2 | 0.991 | 181:168 | 0.49 |
| rs7721093 | 40,774,697 | | | | | | | | | | | 197 | 3.0 | 0.335 | 181:168 | 0.49 |
| rs171605 | 40,776,880 | | | | | | | | | | | 198 | 2.2 | 1.000 | 155:146 | 0.60 |
| rs249422 | 40,778,299 | | | | | | | | | | | 199 | 1.4 | 0.334 | 155:146 | 0.60 |
| rs7719692 | 40,779,729 | | | | | | | | | | | 200 | 1.4 | 1.000 | 182:169 | 0.49 |
| rs3792827 | 40,781,090 | | | | | | | | | | | 201 | 1.4 | 0.260 | 182:169 | 0.49 |
| rs3805497 | 40,782,642 | | | | | | | | | | | 202 | 1.6 | 0.158 | 138:130 | 0.63 |
| rs2329353 | 40,784,025 | | | | | | | | | | | 203 | 1.4 | 0.985 | 130:125 | 0.75 |
| rs249425 | 40,788,522 | | | | | | | | | | | 204 | 4.5 | 0.010 | 155:147 | 0.65 |
| rs29743 | 40,791,037 | | | | | | | | | | | 205 | 2.5 | 0.004 | 138:118 | 0.21 |
| rs257009 | 40,806,973 | | | | | | | | | | | 206 | 15.9 | 0.000 | 76:64 | 0.31 |
| rs3805492 | 40,819,020 | | | | | | | | | | | 207 | 12.0 | - | 142:121 | 0.20 |
| rs10053664 | 40,821,568 | 23 | 97.8 | - | 0.43 | 0.44 | 0.43 | 0.48 | 1.12 (0.92-1.35) | 185:171 | 0.46 | | | | | |
