# Supplementary material for: Systematic Association Mapping Identifies NELL1 as a Novel IBD Disease Gene
Source: PLoS One. 2007 Aug 8;2(8):e691. doi: 10.1371/journal.pone.0000691 (PMC1933598; doi:10.1371/journal.pone.0000691)
Supplement: Table S7 — Results of gender-stratified analysis for 5p13.1 SNPs. Only markers that showed a significant p-value in the single-point analysis of the two subgroups are shown. 378 controls and 343 CD cases were randomly drawn from each subgroup and odds ratios (OR) for carriership of the common allele are shown. (0.18 MB PDF) [file pone.0000691.s015.pdf]

Supplementary Table 7: Results of gender-stratified analysis for *5p13.1* SNPs. Only markers that showed a significant p-value in the single-point analysis of the two subgroups are shown. 378 controls and 343 CD cases were randomly drawn from each subgroup and odds ratios (OR) for carriership of the common allele are shown.

			Females						Males					
#	Name	Position	p_{CCA}	p_{CCG}	OR	95% CI	$MAF_{Controls}$	MAF_{Cases}	p_{CCA}	p_{CCG}	OR	95% CI	$MAF_{Controls}$	MAF_{Cases}
7	rs1992662	40,429,609	***0.0035***	***0.0069***	2.14	1.25-3.66	0.35	0.28	0.13	0.35	1.33	0.80-2.21	0.29	0.26
8	rs1992660	40,450,824	***0.0057***	***0.019***	1.73	1.11-2.69	0.40	0.33	0.10	0.29	1.32	0.84-2.05	0.36	0.32
11	rs7725052	40,523,027	***0.0016***	***0.0013***	2.12	1.39-3.23	0.46	0.38	0.16	0.31	1.34	0.91-1.99	0.42	0.38
12	rs1553575	40,538,689	***0.00021***	***0.00048***	1.74	1.03-2.97	0.37	0.28	0.26	0.26	1.55	0.92-2.60	0.32	0.29
15	rs6451525	40,590,026	***0.0011***	***0.0019***	1.44	0.78-2.66	0.31	0.23	0.20	0.25	1.63	0.91-2.92	0.28	0.25
18	rs4409138	40,639,362	***0.032***	0.10	1.47	0.48-4.55	0.12	0.09	1.00	0.59	0.45	0.08-2.48	0.10	0.10
19	rs924967	40,650,879	***0.033***	0.11	1.47	0.48-4.54	0.12	0.09	0.94	0.57	0.45	0.08-2.47	0.10	0.10

			Females		Males		Females		Males		Females		Males	
#	Name	Position	$f11_{Controls}$	$f11_{Cases}$	$f11_{Controls}$	$f11_{Cases}$	$f12_{Controls}$	$f12_{Cases}$	$f12_{Controls}$	$f12_{Cases}$	$f22_{Controls}$	$f22_{Cases}$	$f22_{Controls}$	$f22_{Cases}$
7	rs1992662	40,429,609	0.426	0.509	0.517	0.564	0.451	0.430	0.377	0.354	0.123	0.061	0.106	0.082
8	rs1992660	40,450,824	0.362	0.440	0.430	0.482	0.472	0.456	0.426	0.405	0.166	0.104	0.144	0.113
11	rs7725052	40,523,027	0.283	0.351	0.362	0.393	0.509	0.539	0.443	0.455	0.208	0.110	0.195	0.152
12	rs1553575	40,538,689	0.372	0.512	0.475	0.494	0.516	0.421	0.416	0.432	0.112	0.067	0.109	0.074
15	rs6451525	40,590,026	0.458	0.589	0.537	0.564	0.468	0.358	0.376	0.380	0.074	0.053	0.087	0.056
18	rs4409138	40,639,362	0.773	0.836	0.806	0.812	0.206	0.149	0.189	0.176	0.021	0.015	0.005	0.012
19	rs924967	40,650,879	0.774	0.836	0.807	0.815	0.205	0.149	0.188	0.173	0.021	0.015	0.005	0.012
