# Supplementary material for: Systematic Association Mapping Identifies NELL1 as a Novel IBD Disease Gene
Source: PLoS One. 2007 Aug 8;2(8):e691. doi: 10.1371/journal.pone.0000691 (PMC1933598; doi:10.1371/journal.pone.0000691)
Supplement: Table S8 — SNP coverage of 100k Array for known IBD susceptibility loci. Variants of Table 4 were included (highlighted by grey shading) to calculate distances. Nucleotide positions refer to NCBI build 35 and the following gene regions were used: IL23R: 67002085-67095564, ATG16L1: 234447031-234491134, DLG5: 78895154-79030951, NOD2: 50509083-50545020. (0.16 MB PDF) [file pone.0000691.s016.pdf]

Supplementary Table 8: SNP coverage of 100k Array for known IBD susceptibility loci. Variants of Table 4 were included (highlighted by grey shading) to calculate distances. Nucleotide positions refer to NCBI build 35 and the following gene regions were used:

IL23R: 67002085-67095564, *ATG16L1*: 234447031-234491134, *DLG5*: 78895154-79030951, *NOD2*: 50509083-50545020

Gene	Name	rs_number	built35	distance [kb]	CR	MAF	HWE	p_{CCA}	p_{CCG}
NOD2	**SNP 8/Arg702Trp**	rs2066844	49,303,427						
NOD2		rs10521209	49,313,210	9.78	0.99	0.41	0.875429	1.26E-03	3.12E-03
NOD2	**SNP 12/Gly908Arg**	rs2066845	49,314,041	0.83					
NOD2		rs2076756	49,314,382	0.34	1.00	0.26	0.529660	1.93E-13	2.04E-12
NOD2	**SNP 13/Leu1007fs**	rs2066847	49,321,279	6.90					
IL23R		rs2863202	67,296,356		1.00	0.19	0.877133	3.92E-01	1.63E-01
IL23R		rs10489631	67,313,136	16.78	1.00	0.44	0.780629	5.81E-01	7.08E-01
IL23R		rs1321156	67,316,723	3.59	0.96	0.14	0.101553	3.73E-01	6.84E-01
IL23R		rs10489630	67,374,643	57.92	0.98	0.38	0.724107	1.39E-01	1.09E-01
IL23R		rs2863212	67,397,137	22.49	0.95	0.12	0.189607	3.38E-01	4.78E-01
IL23R		rs10489629	67,400,370	3.23	1.00	0.43	0.558801	9.62E-02	2.30E-01
IL23R		rs10489628	67,416,128	15.76	0.95	0.37	0.749220	5.27E-01	3.89E-01
IL23R	**Arg381Gln**	rs11209026	67,417,979	1.85					
IL23R		rs4319296	67,489,611	71.63	1.00	0.03	0.232389	3.03E-01	2.46E-01
IL23R		rs10489627	67,491,697	2.09	0.99	0.33	0.801662	3.44E-01	2.71E-01
DLG5		rs1344967	79,197,624		1.00	0.28	0.459024	7.95E-01	3.79E-01
DLG5		rs1561438	79,198,099	0.48	0.95	0.27	0.248734	6.30E-01	2.94E-01
DLG5	**DLG5_e26**	-	79,236,487	38.39					
DLG5	**Pro1371Gln**	rs2289310	79,240,879	4.39					
DLG5		rs1866435	79,246,939	6.06	0.97	0.02	0.074601	6.64E-01	9.03E-01
DLG5		rs1248630	79,253,862	6.92	0.93	0.27	0.993150	3.14E-01	1.16E-01
DLG5		rs1248671	79,273,832	19.97	0.99	0.35	0.565555	2.98E-01	4.21E-01
DLG5	**Arg30Gln**	rs1248696	79,286,611	12.78					
DLG5		rs7895188	79,293,384	6.77	0.98	0.32	0.838004	7.79E-02	1.60E-01
DLG5		rs10509396	79,412,406	119.02	1.00	0.00	0.980005	3.21E-01	-1.00E+00
ATG16L1	**Thr300Ala**	rs2241880	233,965,368						
ATG16L1		-	-		-		-	-	-
